# Deep interpretable radiogenomic workflow deciphers tumor microenvironment from breast MRI and identifies clinician-interpretable biomarkers

**DOI:** 10.1038/s41698-026-01458-2

**Published:** 2026-05-06

**Authors:** Huijun Li, Qiuxia Yang, Rui Zhang, Yize Mao, Xiaoli Li, Zequn Zhang, Yuxi Chen, Feng Zou, Chon Lok Lei, Peng Wang, Hongyan Wu

**Affiliations:** 1 Faculty of Health Sciences, University of Macau, Macao SAR, China; 2 Shenzhen Institute of Advanced Technology, Chinese Academy of Sciences, Shenzhen, China; 3 State Key Laboratory of Oncology in South China, Guangdong Provincial Clinical Research Center for Cancer, Sun Yat-sen University Cancer Center, Guangzhou, China; 4 Faculty of Science and Technology, University of Macau, Macau SAR, China; 5 University of Chinese Academy of Sciences, Beijing, China

**Keywords:** Biomarkers, Cancer, Computational biology and bioinformatics, Oncology

## Abstract

The tumor microenvironment (TME) influences tumor prognosis and response to immunotherapy. However, current TME assessments primarily rely on invasive pathology slices. Moreover, tumor heterogeneity poses challenges in identifying reliable biomarkers for accurate assessments of TME. We present a general interpretable workflow that deeply correlates magnetic resonance imaging (MRI) and TME. This workflow deconvolutes bulk data to infer a reliable TME profile, enables unsupervised lesion annotation with incorporated TME information, and identifies cancer imaging biomarkers and subtypes using interpretable radiomic features that are readily understandable by clinicians. Interpretable modules of gene and image data improve biomarker discovery and clinical application. The customized deconvolution outperforms existing baselines across multiple datasets, and it initially revealed an inverse relationship between the proportion of cancer-associated fibroblasts (CAFs) and T-cell infiltration in triple-negative breast cancer (TNBC). The radiogenomics model achieved an accuracy of 0.87 in predicting the proportion of CAFs and identified novel, robust microenvironment imaging biomarkers, specifically associated with CAFs. The radiomic features we identified for subtyping exhibited consistent distributions across breast cancer patients and obtained an average accuracy of more than 0.8 in five multicenter validations.

## Introduction

Cancer development, growth and metastasis are closely related to the internal and external environments in which tumor cells reside^1^. The tumor microenvironment (TME) involves various immune cell types, cancer-associated fibroblasts (CAFs), endothelial cells, pericytes, and other tissue-resident cell types. According to the characteristics and changes of the TME, personalized treatment programs can be formulated to improve the therapeutic effect and the survival rate of patients^2–4^. Nevertheless, analyzing TME typically necessitates invasive surgery or biopsies with significant risks to acquire tumor tissue, and often leads to an information lag. A preoperative, non-invasive microenvironment analysis method may improve the accuracy of early cancer diagnosis and immunotherapy response prediction and provide a valuable reference to guide clinical care.

Radiogenomics aims to achieve non-invasive detection by correlating paired genomics and images to identify imaging features of genomic signatures^5–10^. Zhou et al.^11^ built a rough radiogenomic map to associate radiomic features with metagenes in non-small cell lung cancer using t-statistics and Spearman’s correlation metrics. However, the research did not delve into the analysis of the tumor microenvironment. Su et al.^6^ established radiomic signatures that can predict the level of tumor-infiltrating lymphocytes (TILs) in TNBC, in which TIL densities were evaluated on hematoxylin and eosin staining (H&E) slides. Deconvolution is proposed to estimate TME using bulk gene expression data, enabling the reuse of paired bulk genomic and image data while overcoming limitations from scarce paired single-cell and image data. Some studies have introduced unsupervised deconvolution approaches^8^. Li et al.^5^ identified prognostic subclones in breast cancer using an unsupervised method. This unsupervised approach makes it difficult to reveal TME involving fine-grained cell types. Other current deconvolution methods rely on predetermined reference expression to guide the deconvolution process, such as CIBERSORT^12^, or established gene signatures like xCell^13^. The previously published expression matrix and fixed reference may apply to one group but fail in another due to tumor heterogeneity.

Despite these initial efforts, a robust and interpretable approach deeply correlating imaging biomarkers with TME is urgently needed and faces significant challenges due to tumor heterogeneity. We proposed a workflow of the deep interpretable radiogenomic framework (DiRG), obtained TME components from bulk data, and correlated the medical images with the TME to obtain TME-related image markers, resulting in noninvasive subtyping for breast. Our study explores an innovative non-invasive cancer diagnosis workflow that can fully reflect TME, adapt to tumor heterogeneity, and be interpretable. Triple-negative breast cancer (TNBC) accounts for 12−17% of all pathological types of breast cancer, has specific biological behavior and clinicopathological features, and has a poorer prognosis than other types^14^. We validated our entire workflow using multicenter cohorts of breast cancer patients, including TNBC and non-TNBC cases.

Our workflow first proposes a deconvolution algorithm, iBayesPrism, which leverages BayesPrism^15^ in conjunction with a customized and iteratively updated reference matrix to infer TME. We then built a suite of deep radiogenomic models to investigate the association of images with TME and subtypes. By pinpointing the crucial image features and regions most relevant to TME, we successfully identified imaging biomarkers and developed a general unsupervised lesion annotation method incorporating immune microenvironment information. Based on the identified imaging markers, we developed an interpretable model for non-invasive subtyping using imaging data alone. In contrast to conventional approaches, our subtyping model incorporated microenvironment information through the utilization of CAF-specific markers. Moreover, we introduce gene and image feature importance modules designed to enhance the interpretability of the framework, facilitating advanced marker gene discovery and clinical practice.

The inferred TME reveals differences in the infiltrability of TNBC and non-TNBC groups, and the differentially expressed genes (DEGs) and enrichment analysis on the gene expression reveal meaningful biological functions. The comparison between the two groups reveals a novel finding: an inverse relationship between the proportion of cancer-associated fibroblasts (CAFs) and the level of T-cell infiltration. The identified CAF-related radiomic imaging biomarkers exhibit a high degree of stability and reproducibility in subtyping TNBC and non-TNBC. In conjunction with the interplay between CAFs and tumor-infiltrating T cells, these biomarkers provide promising support for non-invasive tumor subtyping as well as immunotherapy response prediction, based solely on easily accessible MRI data.

This Workflow allows us to gain insights into the biological properties and characteristics of tumors without the need for surgical intervention, thereby facilitating early detection and personalized treatment plans.

## Results

### Workflow

**Fig. 1:**
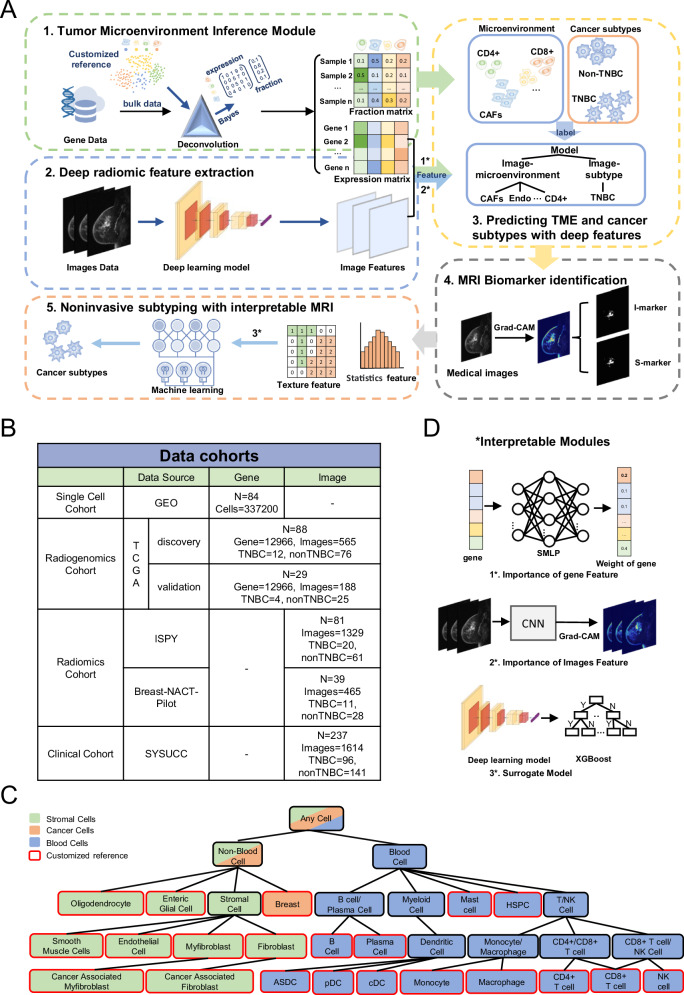
Overview of the deep interpretable radiogenomic framework and datasets. **A** Workflow: Cell type proportion and expression were obtained through deconvolution; Gene expression was combined with image features extracted by the UNet module to predict TME and cancer subtypes, constructing image-microenvironment and image-cancer subtype associations; Use attention mechanism to extract related biomarkers for non-invasive cancer typing based on an interpretable Grad-CAM model; Establish non-invasive subtyping models. **B** Description for data cohorts. **C** Customized reference cell types for deconvolution (More fine-grained reference cells can be customized with the whole cellular hierarchy, as shown in Supplementary Fig. S1). **D** Interpretable modules, including Grad-CAM, SMLP and XGBoost surrogate model, corresponding to the three “*” marks in panel (**A**), respectively.

The entire workflow of the deep interpretable radiogenomic framework, hereafter referred to as DiRG, consists of five steps (Fig. 1A). First, we constructed a TME inference (TMI) module, based on a deconvolution method, inferring the proportion and expression of the specific cell types of bulk data. Next, we employed a deep learning model to extract the deep features in medical images. Then, we developed models to predict TME and cancer subtypes with radiogenomic information obtained in the first two steps. Furthermore, we identified the significant MRI features based on the attention method, resulting in image markers. Finally, based on image markers, we constructed a noninvasive interpretable subtyping module.

We enrolled 479 breast cancer patients and 79 lung cancer patients from five cohorts (Fig. 1B). The multicenter validation included a single-cell cohort from the Gene Expression Omnibus (GEO), a radiogenomics cohort from The Cancer Genome Atlas Program (TCGA), radiomics cohorts from a therapeutic response study (ISPY1) and a neoadjuvant chemotherapy study (Breast-MRI-NACT-Pilot), as well as a clinical cohort from Sun Yat-Sen University Cancer Center (SYSUCC), demonstrating the generality and robustness of our framework. The single-cell cohort was used to validate our deconvolution method. The radiogenomics discovery cohort was used to construct our radiogenomic models and to identify imaging markers. The identified markers will be validated in the radiogenomics validation cohort and subsequently utilized in the radiomics and clinical cohorts.

The first step is to construct the TME inference (TMI) module. To adapt to tumor heterogeneity, we enhanced BayesPrism^15^ by introducing a customizable approach that iteratively updates the reference matrix. This new method, named iBayesPrism, deconvolutes cell proportions and gene expressions for each cell type from bulk RNA sequencing data.

Bayesprism adjusts gene expression matrix to fit the bulk expression iteratively, which makes the matrix adapt to diverse tumor samples. The joint posterior distribution *P*(*μ*_*n*_, *U*_*n*_∣*φ*, *X*_*n*_) of the proportion of cell states *μ*_*n*_ and gene expression *U*_*n*_ of the *n*th bulk sample is iteratively sampled and updated to fit the bulk expression *X*_*n*_, based on the single-cell reference matrix *φ* as the prior distribution. Instead of using a fixed reference matrix produced by a certain group, Bayesprism adjusts the gene expression matrix to fit the bulk expression in each iteration, which makes the matrix adapt to tumor heterogeneity.

We further customized a reference matrix by introducing more interesting cell groups instead of using the given expression matrix, as the current deconvolution methods have done. Considering that one of our main goals is to correlate MRI with TME, we note that some rare cell types are unnecessary and could induce noise. To reduce the noise and obtain a reliable gene expression reference matrix, we utilize scATOMIC^16^, a single-cell annotation tool of TMEs, to customize the reference matrix. The scATOMIC structured a pan-cancer TME cellular hierarchy where each parent node represents a group of related cells, and each terminal node represents a single-cell class. We focus exclusively on cell types highly associated with breast cancer. We selected 20 groups of non-cancer cells and breast cancer cells to deconvolute the bulk data with iBayesprism, as shown in Fig. 1C. We chose the cellular components that accounted for a larger portion of the initial deconvolution as the final reference matrix.

We finally obtained the gene expression and proportion of 8 cell types (B cells, CD4+ T cells, CD8+ T cells, CAFs, fibroblasts, breast cancer, macrophages and endothelial cells), of which breast cancer is the purified breast cancer cells. We categorize the TME cell proportions in the fraction matrix into TME labels–high and low groups.

The second step is deep radiomic feature extraction. To extract different image features of cancer subtypes and microenvironments, we employed a deep learning model, UNet^17^, to perform feature extraction under the supervision of the cancer subtype. UNet allows the network to capture multi-scale feature information during encoding and decoding. Moreover, UNet is suitable for different types of image segmentation tasks, and it can be trained end-to-end with a limited number of images.

The third step is to predict TME and cancer subtypes with radiogenomic information. We constructed a suite of deep radiogenomic models to predict TME and cancer subtypes. These models utilized paired imaging data and gene expression profiles as inputs, with outputs consisting of microenvironment labels (low and high) and subtype labels. Specifically, in our models, we employed only the expression matrix derived from the first step as the genomic features, while the fraction matrix served as the output—the criterion for grouping TME into high and low categories. The SMLP module is utilized for the feature embedding of gene expression data, which enhances model interpretability and reveals the impact of input features on prediction results by adding a pre-single connection layer before the Multilayer Perceptron (MLP) input layer^18^, as shown in Fig. 1C. We combined the gene feature vector and image vector obtained from the UNet module, and fed them into a three-layer fully connected neural network to perform the final classification.

We built prediction models separately for each specific association (image-TNBC, image-CAF, image-CD4^+^ T cell, image-Endothelial, image-Macrophage). These models are designed to capture key radiogenomic information related to tumor subtype or immune infiltration, thus providing possible support for tumor classification and grading as well as immunotherapy response prediction.

The fourth step is to obtain the MRI biomarker and lesion annotation. After getting the five trained models, we identify the crucial image features and regions most relevant to the subtypes or TME with Gradient-weighted Class Activation Mapping (Grad-CAM)^19^. Using gradient information to generate heat maps showing the regions of interest of the network, namely, the corresponding markers. Image annotations can be obtained from the extraction of weighting parameters. The upper 60% portion of the attention score is extracted and converted into a lesion annotation.

Our models effectively integrate genomic features with imaging features. Incorporating Grad-CAM, the well-trained architecture facilitates unsupervised segmentation of subtype or microenvironment markers directly from image data, eliminating the need for additional genomic input. We successfully identified robust immune microenvironment imaging markers (I-markers), specifically the CAF-I-markers. Different from the current annotation methods, our annotation is an unsupervised lesion annotation method that incorporates immune microenvironment information. The approach will be further validated and utilized in the subsequent analyses for noninvasive subtyping in the multicenter study, ensuring robustness and generalizability across diverse datasets.

The fifth step is to perform noninvasive subtyping with interpretable MRI features. Based on the I-markers we obtained in the fourth step, we developed an interpretable model for noninvasive subtyping with only image data. We used radiomic features as the model feature to make the model more interpretable by clinicians.

First, we extract the radiomic features from I-markers, respectively, including CAF-I-markers, CD4^+^-I-markers, Endo-I-markers, Macro-I-markers. We extract 36 radiomic features, including 10 first-order features, 10 Shape2D features, and 16 Gray Level Co-occurrence Matrix (GLCM) features (the details of radiomic feature extraction are provided in the section “Data processing and radiomic feature extraction”). Using the T-test, we analyzed the differences in radiomic features between the TNBC and non-TNBC groups, identifying significant feature distribution between these two groups. Similarly, we extracted radiomic features from the radiogenomics discovery cohort and analyzed their distribution as the ground truth. Next, we compared the feature distributions extracted from other cohorts with the ground truth distribution. We found that the radiomic features associated with the CAF-I markers exhibit a high degree of similarity to the ground truth across all datasets. Finally, we constructed a surrogate subtyping model based on the eXtreme Gradient Boosting (XGBoost) method^20^, which utilized the radiomic features extracted from CAF-I-markers.

### Performance of deconvolution outperforms existing baselines across multiple datasets

We performed deconvolution of the single cell cohort based on our iBayesPrism method and the customized reference matrix, as shown in the first step of the workflow. In the absence of paired bulk and single-cell data, following the study^21^, we generated pseudo-bulk data from single-cell datasets (Wu et al.^22^ and GSE136831^23^) by summing all single-cell data of each cell type, which were used as ground truth to validate the reference matrix and deconvolution method.

**Fig. 2:**
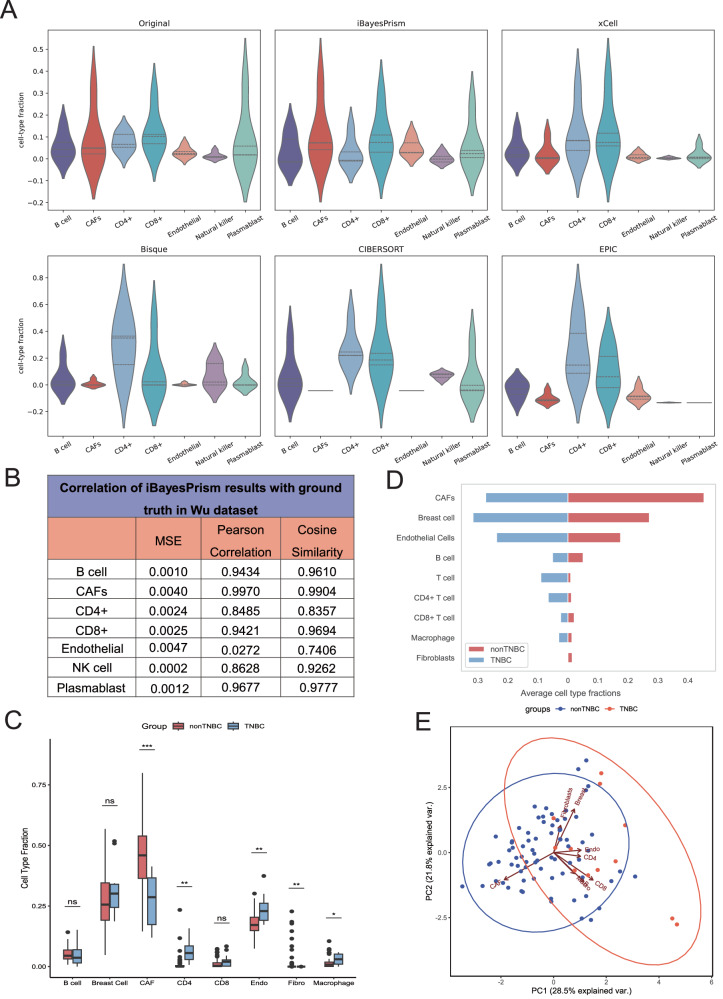
Deconvolution results and statistical analysis. **A** Distribution of cell type proportions for the results of our deconvolution and comparison methods in the Wu dataset. **B** The correlation between the cell type fraction predicted by our methods and the ground truth in the Wu dataset. **C** T-test results and cell type fraction distribution in TNBC and non-TNBC groups. The symbols indicate significance levels as follows: “***” for *p* ≤ 0.001, “**” for *p* ≤ 0.01, “*” for *p* ≤ 0.05, and ‘ns’ for not significant (*p* > 0.05). **D** The average fractions of cell types in TNBC and non-TNBC. T cell is the sum of CD4^+^ T cell and CD8^+^ T cell. **E** PCA results and the vectors indicate the cell type effect in TNBC and non-TNBC groups.

We compared our deconvolution method with current deconvolution methods, including the reference-based methods (Bisque^21^, CIBERSORT^12^, quanTIseq^24^, EPIC^25^), and a gene signatures-based enrichment method (xCell^13^). Since some of the deconvolution methods are based on fixed reference data or given marker genes, these methods may miss out some cell types, as shown in Fig. 2A (The result of quanTIseq is shown in Supplementary Fig. S3F for space limitations). The results show that compared to Bisque^21^, CIBERSORT^12^, xcell^13^, quanTIsseq^24^, and EPIC^25^, the cell proportions obtained after iBayesPrism deconvolution are much closer to the ground truth distribution. We compared the cell types that overlapped with the ground truth and found that our deconvolution method produces cell type proportions with Pearson and cosine similarities greater than 0.9 (Fig. 2B) in the Wu dataset.

We further validated the generalization of our method in lung cancer (GSE136831, results shown in Supplementary Figs. S2–4 and Supplementary Table S1). Our results demonstrated the superiority of our approach in dealing with tumor heterogeneity.

### Deconvoluted cell proportions highlight differential infiltrability across cancer subtypes

We deconvoluted the 88 bulk data in the radiogenomics discovery cohort to measure the different infiltrability between TNBC and non-TNBC by the proportion of cell type. We divided each cell type into two groups according to TNBC and non-TNBC labels, and calculated the proportion differences between the two groups based on the *t*-test and Wilcoxon-test, respectively. Of the eight cell types, five (CD4^+^ T cell, CAFs, fibroblasts, macrophages, and endothelial cells) were significantly different (*p* < 0.05) between the two groups in the t-test, as shown in Fig. 2C. The result shows that the TNBC group has a lower proportion of CAF, higher proportion of CD4^+^ T cell, macrophages, and endothelial cells. The distribution of fibroblasts was more similar between the TNBC and non-TNBC groups, with differences primarily attributed to discrete points. Wilcoxon test results showed *p*-values greater than 0.05 for fibroblast groups. The detailed results of the Wilcoxon test are presented in supplementary Fig. S5.

Figure 2D shows the mean distribution of each cell type in the TNBC and non-TNBC groups, which shows a significant difference in the immune microenvironment between the two groups. Figure 2E shows the principal component analysis (PCA) results of cell type fractions, with the length of the vectors indicating the influence on the results and the angle between the vectors indicating the correlation between the dimensions. It shows that CAFs have the biggest effect on the non-TNBC and TNBC groups.

We further validated our results using the radiogenomics validation cohort. We obtained consistent results indicating that CAFs have a lower distribution, while CD4^+^ T cells, endothelial cells, and macrophages have a higher distribution in the TNBC group, similar to what was observed in the radiogenomics discovery cohort. The detailed results are shown in supplementary Fig. S6.

The results in Fig. 2C show that the TNBC group has a higher proportion of CD4^+^ T cells, macrophages, and endothelial cell, which is consistent with the current research. Research^26^ presents that, for the TME in TNBC, there is a high degree of infiltration of T cells (including CD4^+^, CD8^+^, and Tregs) compared to other subtypes. Compared to the non-TNBC, TNBC has a higher proportion of macrophage^27,28^. Endothelial colony-forming cells (ECFCs) mediate neovascularization, which is a crucial contributor to cancer growth and metastasis. Lee et al. showed that the angiogenic potential of ECFCs was enhanced by TNBC through both direct and indirect mechanisms, and had a higher gene expression profile of pro-angiogenic factors in TNBC compared to non-TNBC^29^.

Interestingly, in the present study, there is no definitive finding regarding the comparison of CAFs between the TNBC and non-TNBC groups. Our deconvolution results—higher CD4^+^ and CD8^+^ and lower CAFs in TNBC compared to non-TNBC— reveal an inverse relationship between CAF proportion and T-cell infiltration. Some research suggests that CAF cells produce a physical barrier and immunosuppressive environment that restricts or traps T cells and prevents them from entering the tumor^30^. Wu et al.^31^ showed that blocking fibroblast growth factor receptor (FGFR) signaling pathways markedly suppressed tumor growth with increased T cell infiltration in immunocompetent mouse models of TNBC. These studies indirectly demonstrate the reasonableness of our results.

Our novel finding elucidates the possible interplay between CAFs and tumor-infiltrating T cells, highlighting mechanisms that may influence immune evasion and therapeutic resistance in breast cancer. Considering the inherent tumor heterogeneity, the finding necessitates further validation in expanded, multicenter cohorts.

### DEGs and GSEA of deconvoluted cell expression reveal the biological function in TNBC and non-TNBC

After obtaining the gene expression of each cell type using our deconvolution method, we then analyzed the gene expression of the breast cancer cells in the TNBC and non-TNBC groups to identify their relevant biological functions. We first analyzed differentially expressed genes (DEGs) between the two groups using *limma*^32^. To better understand the functional significance of DEGs and their biological processes, we performed gene set enrichment analysis (GSEA). Using Clusterprofiler^33^, we performed enrichment analysis on the Kyoto Encyclopedia of Genes and Genomes (KEGG) database. We set *p*≤0.05 as the significance level to select statistically significant enrichment items, and we also considered the number of genes included in the enrichment items and the Normalized Enrichment Score (NES) to evaluate the reliability of the enrichment results comprehensively.

**Fig. 3:**
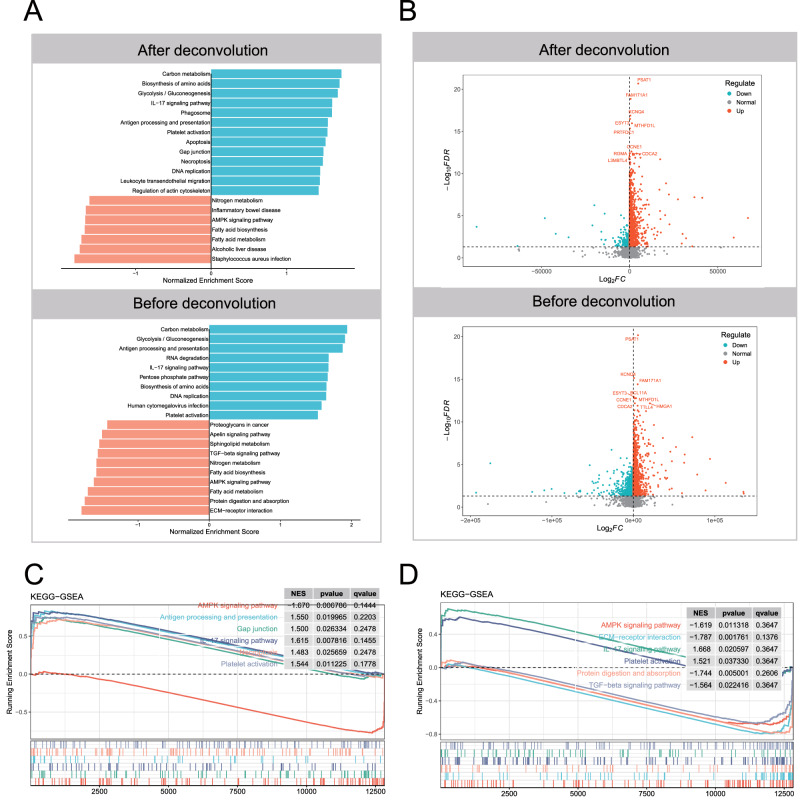
Gene set enrichment analysis results. **A** Enrichment pathways after deconvolution and in bulk data (before deconvolution). This breast cancer gene expression profile is enriched in pathways with an absolute NES greater than 1 and a *p*-value less than 0.05. **B** The DEGs results after deconvolution and in bulk data. **C** Details of some enrichment pathway results after deconvolution. **D** Details of some enrichment pathway results of bulk data.

Figure 3A, B show DEG analysis and enrichment pathways after and before deconvolution, respectively. After deconvolution, most of the genes (PSAT1, FAM171A1, CCNE1, RGMA) (Fig. 3B) were significantly up-regulated and also found to be associated with TNBC^34–37^. The descriptions of the top 10 genes are shown in Supplementary Tables S2 and S3. As shown in Fig. 3C, the enrichment analysis showed that the DEGs were significantly enriched in biological pathways of the interleukin 17 (IL-17) signaling pathway, AMP-activated protein kinase (AMPK) signaling pathway, antigen processing and presentation pathways, and gap junction pathway. IL-17 promotes inflammation and acts synergistically with tumor necrosis factor and interleukin-1. Activation of IL-17 signaling is frequently observed in the pathogenesis of various autoimmune diseases, and IL-17A is considered to be tumor-promoting in TNBC^38^. Cao et al.^39^ summarized the possible role of the AMPK signaling pathway in the regulation of TNBC growth and survival and in the development of drug resistance. Antigen processing and presentation are cornerstones of adaptive immunity, and several studies have confirmed that downregulation of proteins involved in antigen presentation pathways is associated with poor outcomes in TNBC patients^40^. The gap junction communication channels formed by connexins have been reported to exhibit tumor-suppressive functions, including in TNBC; Fu et al.^41^ demonstrated that overexpression of connexin 43 targets *β*-microtubulin to modulate paclitaxel resistance in TNBC cells.

Figure 3B shows the corresponding DEG analysis of the bulk data. DEGs overlap before and after deconvolution, including PSAT1, FAM171A1, KCNQ4, ESYT3, MTHFD1L, CCNE1, and CDCA2. Figure 3C shows that there are some new pathways after deconvolution, such as the gap junction pathway, indicating that the deconvoluted data may reveal new and relevant information while preserving the original functionality.

### Integrated radiogenomic features enable precise microenvironment and subtype prediction

Based on the radiogenomics discovery cohort, we extracted deep radiomic features (the second step in Fig. 1A) and developed five submodels to correlate the tumor microenvironment and cancer subtypes with medical images (the third step in Fig. 1A). The image-TNBC model shows whether a patient belongs to TNBC or non-TNBC, while image-CAF indicates the status of the CAF being high or low. Image-CD4^+^ T cell, image-Endo, and image-Macro indicate the status of CD4^+^, endothelial, and macrophage, respectively. Methods section “Models predicting TME and cancer subtypes” and Supplementary Fig. S7 present the details. We identified the corresponding image markers with gradient-weighted class activation mapping (Grad-CAM, the fourth step in Fig. 1A).

**Fig. 4:**
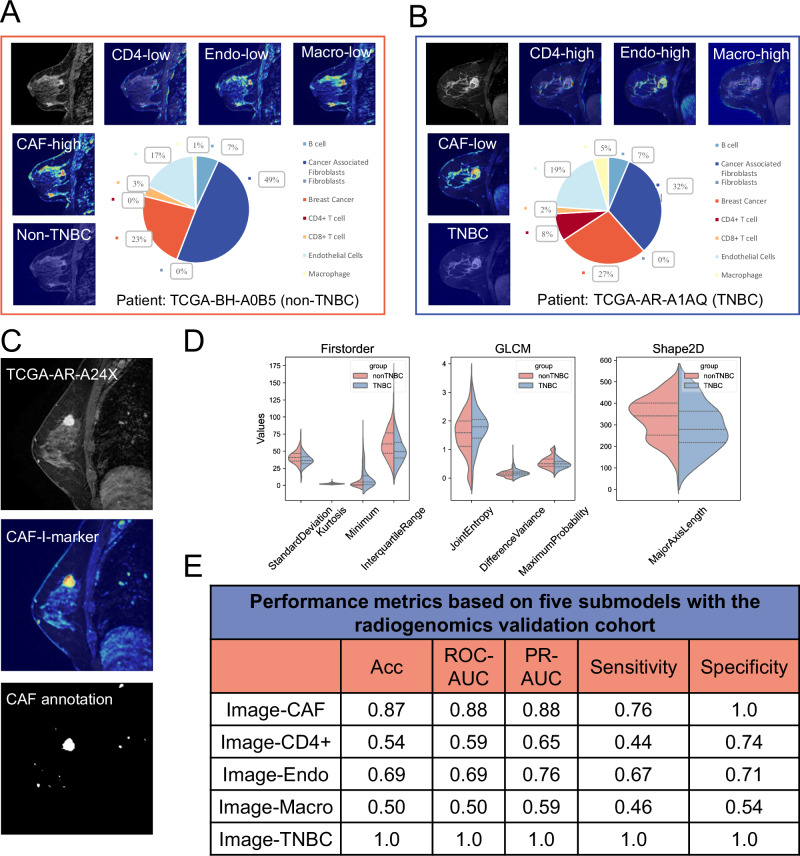
Performance in radiogenomics discovery and independent validation cohorts. **A** The image markers in microenvironment and non-TNBC groups from patient ID TCGA-BH-A0B5. **B** The image markers in microenvironment and TNBC groups from patient ID TCGA-AR-A1AQ. **C** Obtaining CAF-related annotation from CAF-I-marker. **D** The distribution of significant features of CAF-related radiomic features from CAF annotation. **E** The assessment metric results of the five submodels (image-CAF, image-CD4, image-Endo, image-Macro and image-TNBC) with the radiogenomics validation cohort.

Figure 4A, B shows the distributions of the microenvironmental proportions of two patients. Figure 4A (patient TCGA-BH-A0B5) shows the crucial regions in the predictions of CAF-high, CD4^+^-low and endothelial-low (Endo-low), macrophage-low (Macro-low), non-TNBC, respectively. Figure 4B (patient TCGA-AR-A1AQ) belongs to the CAF-low, CD4^+^-high, Endo-high, Macro-high and TNBC group. There were larger attentional areas in the CAF-high group than in those of the CAF-low group, revealing a strong correlation between the microenvironment and the obtained image features. Moreover, it can be observed that both CAF-high and CAF-low groups show the surrounding area of the tumor region with high attention scores. Similarly, the endothelial high and low groups showed closer attention in the tumor surrounding area, while the CD4^+^ T cell and macrophage groups performed less effectively. However, due to data imbalance between the TNBC and non-TNBC groups (with a ratio of 12:76), we were unable to obtain a stable attention area surrounding the tumor in both groups. Consequently, we will instead analyze TNBC using microenvironment-related markers, such as CAF-I-marker and Endo-I-marker, in the subsequent analyses.

Assessed by five-fold cross-validation on the radiogenomics discovery cohort, the image-TME and image-subtype models demonstrated perfect accuracy (1.0). We further validated our five submodels with an independent radiogenomics validation cohort (*N* = 29) by predicting their tumor microenvironment and cancer subtypes. The accuracy and other metrics of these submodels are shown in Fig. 4E. As for the image-TNBC model, the radiogenomics validation cohort achieved a *prediction* accuracy of 1 for the independent validation cohort. Our image-CAF model predicted the CAF-high and low groups with an accuracy of 0.87. Whilst the other submodels slightly suffered from the prediction accuracies with 0.54, 0.69, and 0.50 for the image-CD4^+^, image-Endo, and image-Macro models, respectively, the reason for the different prediction performances of these submodels can be observed in Fig. 4C, where CD4^+^ T cells and macrophages overlapped between the TNBC and non-TNBC groups. Collectively, the high prediction performance achieved by the CAF and TNBC submodels on the independent validation cohort underscored their robustness and reliability when applied to new, unseen datasets.

To further investigate whether gene expression information has been adequately integrated, we also rebuilt these submodels without using genetic data. Supplementary Fig. S8 and Supplementary Table S4 show the Grad-CAM results and metric evaluations for the image-subtype, image-microenvironment submodels. Compared to submodels without genomic data, our submodels exhibit more distinctive immune markers in images and achieve a significant improvement in prediction accuracy after incorporating genomic features. The results demonstrated that by successfully and efficiently associating genomic features with imaging features, our submodels achieved deep integration and complementarity between gene expression and MRI characteristics. This approach enables the identification of genomic properties through medical images.

### Correlating radiomic features with TME to benefit clinical diagnosis

We further investigated the potential of using radiomic features to reflect the status of TME, thereby enhancing the interpretability of TME and providing clinically relevant, actionable insights derived from these medical images. We extracted radiomic features annotated by microenvironment markers as presented in the fifth step of the workflow (The description of all 36 radiomic features is shown in Supplementary Table S5). We used a T-test to analyze the difference in radiomic features between the TNBC and non-TNBC groups.

Figure 4C shows the CAF-I-marker and its annotation in the radiogenomics discovery cohort. Figure 4D illustrates all the CAF-related significant features in the TNBC and non-TNBC groups (The T-test results shown in Supplementary Table S6); the distributions of all 36 CAF-related radiomic features are shown in Supplementary Fig. S9.

Among the significant features (*p* < 0.05), the TNBC group has higher values in Minimum, JointEntropy, and DifferenceVariance of marker region values than the non-TNBC group, while having lower values in StandardDeviation, Kurtosis, InterquartileRange, MaximumProbability, and MajorAxisLength of marker region values than the non-TNBC group. In these features, kurtosis is a measure of the ‘peakedness’ of the distribution of values in the image region of interest (ROI). The minimum is the minimum gray level intensity within the ROI. Interquartile range = P75–P25, where P25 and P75 are the 25th and 75th percentiles of the gray level intensity within the ROI, respectively. Standard Deviation measures the amount of variation or dispersion from the Mean Value. MajorAxisLength is the largest axis length of the ROI enclosing ellipsoid. Difference Variance is a measure of heterogeneity that places higher weights on differing intensity level pairs that deviate more from the mean. Maximum Probability is occurrences of the most predominant pair of neighboring intensity values, while joint entropy is a measure of the randomness/variability in neighborhood intensity values. These findings suggest that we can obtain significant differences in radiomic features between the TNBC and non-TNBC groups.

Moreover, we divided the CAF-related radiomic features into CAF-high and low groups to reveal the correlation of CAF proportion and radiomic features (Supplementary Table S7). There are 20 significant features (*p* < 0.05) in CAF-high and -low groups. Notably, these radiomic features reflect underlying tumor biology in a manner consistent with CAF-related histopathological modeling. The reduced Sphericity and increased MaximumDiameter indicate more irregular and elongated tumor morphology, which aligns with CAF-driven stromal contraction and invasive growth patterns. Higher Entropy and StandardDeviation suggest greater intratumoral heterogeneity, likely arising from CAF-induced heterogeneous extracellular matrix deposition and cellular density. Elevated Imc1 (information measure of correlation) and SumAverage capture increased local texture complexity, corresponding to disorganized collagen fibers and chaotic microarchitecture typical of CAF-rich tumors. Moreover, recent radiogenomic studies have established associations between radiomic features and CAF abundance^42,43^. Our results align with recent radiogenomic studies demonstrating that radiomic features can capture CAF-mediated stromal remodeling. Importantly, our findings extend prior work by linking CAF-associated radiomic features to TNBC subtype, suggesting that these imaging markers reflect not only CAF abundance but also tumor aggressiveness.

Also, the results of endo-related radiomic features (Supplementary Fig. S10 and Supplementary Table S8) show that there are six features with significant differences in TNBC and non-TNBC groups. It shows a similar distribution with CAF-related radiomic features, indicating they both can obtain TNBC-related information.

Further validation was performed on the radiogenomics validation cohort. There are 17 out of 36 features that show the same distribution as in the radiogenomics discovery cohort (Supplementary Fig. S11). Our multicenter independent validation demonstrates that CAF-related radiomic features exhibit a high degree of stability and reproducibility in subtyping TNBC and non-TNBC.

### Non-invasive detection in the multicenter validation study

We developed a subtyping model in the multicenter validation study comprising five independent datasets, relying solely on image-derived features and excluding genomic data. From our previous observations, we noted that radiomic features extracted using CAF-I markers exhibit superior performance compared to other TME-related markers. The Grad-CAM methods used gradient information to generate heatmaps highlighting the network’s regions of interest. We evaluated the best heatmap attention-area threshold (0.5, 0.6, and 0.7–corresponding to the upper 50%, 60%, and 70% of attention areas) in our results in Table 1. The threshold of 0.6 improves sensitivity while increasing ROC-AUC (0.85) and PR-AUC (0.79), reflecting better overall model performance.

**Table 1:** The results of different thresholds of heatmap attention scores in the radiogenomic discovery cohort

Threshold	Accuracy	ROC-AUC	PR-AUC	Sensitivity	Specificity
0.5	0.82	0.82	0.75	0.92	0.80
0.6	0.77	0.85	0.79	1.0	0.74
0.7	0.77	0.83	0.74	0.92	0.75

Therefore, we adopted CAF annotations for radiomic feature extraction with a Grad-CAM heatmap threshold of 0.6. We trained and tested the XGBoost model based on each cohort due to the heterogeneity of the data caused by different devices and other factors. We then perform a consensus analysis on the XGBoost results.

**Fig. 5:**
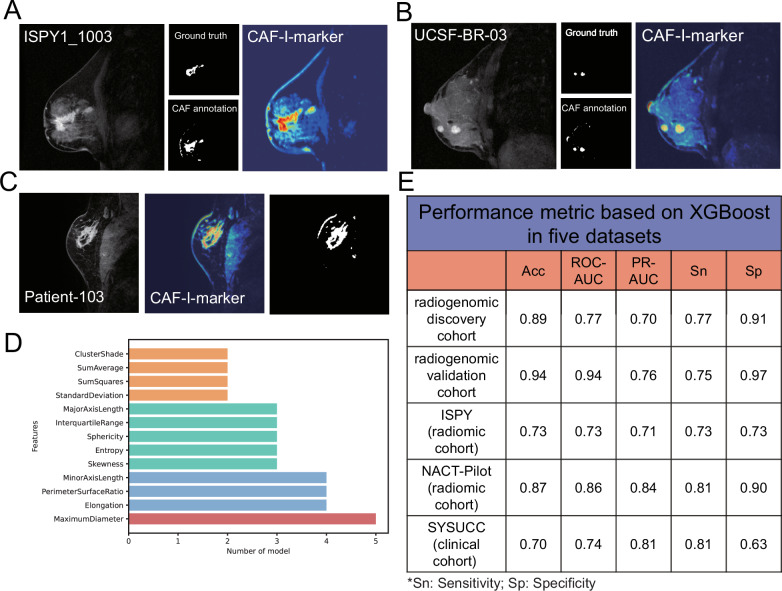
Performance in the radiomics cohort and clinical cohort, and results of the surrogate model. **A**, **B** The CAF-I-markers and related CAF annotation in the radiomics cohort of ISPY and NACT-Pilot, respectively, compared with ground truth. **C** The CAF-I-markers and related CAF annotation in the clinical cohort. **D** Number of models that overlap in important radiomic features (the x-axis is the number of overlap models of the features). **E** The assessment metric results of XGB in the radiogenomics discovery cohort, radiogenomics validation cohort, ISPY of the radiomics cohort, NACT-Pilot of the radiomics cohort, and clinical cohort.

Figure 5A–C illustrates the CAF-I-marker and its corresponding CAF annotation obtained on the samples from ISPY, NACT-Pilot, and the clinical cohort, respectively, using our image-CAF model. Figure 5A, B also present the ground truth annotations of the tumor images. The results of our XGBoost model are shown in Fig. 5E, with prediction accuracies of 0.89 for the radiogenomics discovery cohort, 0.94 for the radiogenomics validation cohort, 0.73 for the ISPY1 dataset, 0.87 for the NACT-Pilot dataset, and 0.70 for the SYSUCC dataset.

Across all five validation datasets, XGBoost demonstrated superior or consistently competitive performance in key metrics, particularly in PR-AUC and sensitivity relative to other machine learning approaches (as detailed in Fig. 5E and Table 2 for RF and SVM). This indicates its strong capability to handle potential class imbalance and deliver robust classification.

**Table 2:** Test results of RandomForest and SVM in five datasets

	Accuracy		ROC-AUC		PR-AUC		Sensitivity		Specificity	
	RF	SVM	RF	SVM	RF	SVM	RF	SVM	RF	SVM
Radiogenomic discovery cohort	0.88	0.88	0.71	0.66	0.50	0.50	0.0	0.0	1.0	1.0
Radiogenomic validation cohort	0.89	0.89	0.84	0.59	0.50	0.50	0.0	0.0	1.0	1.0
ISPY (radiomic cohort)	0.74	0.75	0.64	0.62	0.29	0.52	0.09	0.03	0.96	1.0
NACT-Pilot (radiomic cohort)	0.71	0.65	0.78	0.66	0.53	0.38	0.35	0.19	0.90	0.90
SYSUCC (clinical cohort)	0.69	0.66	0.69	0.64	0.62	0.52	0.49	0.27	0.82	0.93

To validate the effectiveness of our proposed workflow, specifically the effectiveness of TME annotation for noninvasive cancer subtyping, we compared it against a direct image feature extraction and classification approach. We implemented a UNet-based model (UNetFC) that performs image feature extraction followed by classification via fully connected layers for cancer subtyping (detailed in section Methods: “Models predicting TME and cancer subtypes”). As shown in Table 3, XGBoost substantially outperforms UNetFC across all five datasets, achieving accuracies of (0.89, 0.94, 0.73, 0.87, 0.70) compared to (0.13, 0.88, 0.26, 0.34, 0.41) for UNetFC, even on imbalanced data. This demonstrates the superiority of our annotation-guided radiomic features over direct end-to-end image classification for this task.

**Table 3:** Test results of XGBoost and UNetFC in five datasets

	Accuracy		ROC-AUC		PR-AUC		Sensitivity		Specificity	
	XGB	UNet	XGB	UNet	XGB	UNet	XGB	UNet	XGB	UNet
Radiogenomic discovery cohort	0.89	0.13	0.77	0.50	0.70	1.0	0.77	1.0	0.91	0.0
Radiogenomic validation cohort	0.94	0.88	0.94	1.0	0.76	0.50	0.75	0.0	0.97	1.0
ISPY (radiomic cohort)	0.73	0.26	0.73	0.50	0.71	1.0	0.73	1.0	0.73	0.0
NACT-Pilot (radiomic cohort)	0.87	0.34	0.86	0.50	0.84	1.0	0.81	1.0	0.90	0.0
SYSUCC (clinical cohort)	0.70	0.41	0.74	0.50	0.81	1.0	0.81	1.0	0.63	0.0

We then performed a consensus analysis on the five XGBoost models trained on different datasets. First, feature importance analysis was conducted, and the top 10 important features from each model were selected. This process identified 13 overlapping features that appeared in at least two of the models, as shown in Fig. 5D. We found that 6 out of the 13 features—MaximumDiameter, Skewness, InterquartileRange, Entropy, Sphericity and SumAverage of marker region values—exhibit nearly identical distributions across all five datasets.

The distributions of CAF-related radiomic features in TNBC and non-TNBC groups of all datasets are shown in Supplementary Figs. S9, S11–14, respectively. We found that 18 radiomic features in NACT-Pilot shared the same distribution as in the radiogenomics discovery cohort, 20 in ISPY, and 25 in SYSUCC. As shown in Supplementary Fig. S15, the features SumAverage and Sphericity of the marker region show higher values in the TNBC group, while MaximumDiameter, Skewness, InterquartileRange, and Entropy of the marker region values show lower values in the TNBC group.

The consistency of these feature distributions across multiple independent datasets indicated a strong association between these radiomic features and cancer subtypes or specific biological processes. Our results indicated that these features play a crucial role in the non-invasive detection of TNBC, and the uniqueness and prominence they exhibit make them promising to be effective and reliable biomarkers or characterization indicators for accurate identification of TNBC.

For clinical decision-making, the Breast Imaging Reporting and Data System (BI-RADS) assessment is a clinical decision-making process that integrates radiologists’ expertise within a structured framework^44^; however, it lacks fixed, universal quantitative thresholds. To link quantitative radiomic features to established clinical lexicons like BI-RADS is crucial for clinical translation and interpretability. We found that there are some studies to correlate the radiomic features with BI-RADS^45–47^. These features can provide a quantitative basis for semantic descriptors used in BI-RADS. Hu et al. employed statistical methods to prove that the skewness and kurtosis were significantly higher in the malignant group of BI-RADS category 4 than in the benign group (*P* < 0.05)^46^. Luo et al. construct a radiomic score to correlate radiomic features with BI-RADS category^45^. They employed a multivariate regression analysis to obtain 9 selected radiomics features to calculate the radiomics score, and then related the radiomics score to the BI-RADS category. Zhang et al. developed a radiomics model based on MRI images to predict the malignancy of BI-RADS 4 breast lesions^47^. Based on the least absolute shrinkage and selection operator (LASSO) and logistic regression, they obtained 12 important radiomic features, and these features were used to build a radiomics model to predict benign or malignant lesion labels of BI-RADS. Kurtosis and Skewness quantify the peakedness and asymmetry of signal intensity distribution. Moreover, some radiomic features also exhibit associations with the BI-RADS terms in their descriptive aspects. Entropy measures the randomness or disorder of a texture. Higher entropy in malignant lesions correlates with a more disorganized tissue architecture, which may underpin descriptors such as “irregular margins” or “non-mass enhancement” in BI-RADS terms. TNBCs often exhibit larger necrotic areas compared to non-TNBCs, which typically appear as darker regions on imaging^48^. The Minimum feature, representing the lowest grayscale intensity, is thus a potential quantitative indicator corresponding to these necrotic zones.

### DiRG workflow advances marker gene discovery

To analyze the significant genes that cause TNBC and CAF, we interpreted the image-TNBC and image-CAF prediction models with SMLP^18^. SMLP enhances model interpretability and reveals the impact of input features on prediction results by adding a pre-single connection layer before the MLP input layer. Of the top 10 important genes in the image-TNBC model, 7 of them (KRT8, KRT18, CCT6A, ACTB, ENO1, TUBB, and SLC7A5) have already been confirmed as related to the TNBC subtype (Table 4), and some were also shown significant differences in the DEG results in section “DEGs and GSEA of deconvoluted cell expression reveal the biological function in TNBC and non-TNBC,” with CCT6A, ENO1, S100A11, LSR, TUBB, and SLC7A5 significantly upregulated and KRT18 significantly downregulated (Supplementary Table S9).

**Table 4:** The gene name and description of the top 10 important genes in the image-TNBC model

Gene name	Description	Reference
KRT8	The KRT8 is a member of the type II keratin family clustered on the long arm of chromosome 12. Jade Schroeder et al. showed experimentally that chemical exposure may influence the phenotypic transformation of breast cells by affecting the expression of markers such as KRT8 and thus influencing the development and progression of TNBC.	Verified^66^
CCT6A	Yang et al. investigated the expression profile and clinical relevance of chaperonin-containing TCP1 subunit 6A (CCT6A) in TNBC, proving that CCT6A expression in TNBC patients was associated with an adverse prognosis and lymph node metastasis.	Verified^50^
KRT18	The KRT18 encodes the type I intermediate filament chain keratin 18. Mansoor Saleh et al. identified 45 Kenyan women TNBC-specific genes by differential expression analysis, including KRT18.	Verified^49^
CRABP2	Cellular retinoic acid binding protein 2 (CRABP2) belongs to the fatty acid binding protein (FABP) family, which binds with all-trans retinoic acid (RA). Feng et al. suggested that CRABP2 can suppress the invasion and metastasis of ER+ breast cancer and promote the invasion and metastasis of ER- breast cancer by regulating the stability of Lats1 in vitro and in vivo.	Unverified^60^
ACTB	Actin Beta (ACTB) encodes one of six different actin proteins. Md Shahin Alam et al. identified differentially expressed genes (DEGs) between TNBC and control samples, obtained six key genes (TOP2A, BIRC5, AURKB, ACTB, ASPM, and BUB1B) that were upregulated, and one (EGFR) was downregulated.	Verified^51^
ENO1	Enolase 1 (ENO1) encodes alpha-enolase, one of three enolase isoenzymes found in mammals. Radhakrishnan Vishnubalaji et al. proposed that targeted depletion of ENO1 and FDPS halted TNBC cell proliferation, colony formation, and organoid tumor growth under 3-dimensional conditions and increased cell death, suggesting their potential use as novel therapeutic targets for TNBC.	Verified^52^
S100A11	S100 Calcium Binding Protein A11 (S100A11) encodes the protein of the S100 family of proteins containing 2 EF-hand calcium-binding motifs. Lee et al. identified that S100A11, a previously unexplored factor in macrophage-cancer crosstalk, predicts high macrophage density and poor outcomes in ER+ tumors.	Unverified^67^
LSR	Lipolysis Stimulated Lipoprotein Receptor (LSR) is involved in protein localization to the tricellular tight junction and tricellular tight junction assembly. Denise K. Reaves et al. sought to define the role of LSR in breast cancer, proved that LSR may promote collective cell migration, and overexpression of LSR altered gene expression of pathways involved in transformation and tumorigenesis, as well as enhanced proliferation and survival in anchorage-independent conditions.	Unverified^53^
TUBB	Wang et al. investigate the effect of Tubulin beta class I gene (TUBB) on TNBC from the molecular perspective, proving that downregulated TUBB can dampen proliferation, migration and EMT, while facilitating apoptosis of TNBC cells, which might provide new insight for treating TNBC by targeting TUBB.	Verified^54^
SLC7A5	Huang et al. found that solute carrier family 7 member 5 (SLC7A5), an amino acid transporter, was the most important gene and plays a vital role in glutamine metabolism reprogramming in TNBC cells.	Verified^55^

Saleh et al.^49^ identified 45 Kenyan TNBC-specific genes, including KRT18, based on differential expression analysis. Yang et al.^50^ proved that chaperonin-containing TCP1 subunit 6A (CCT6A) expression in TNBC patients was associated with an adverse prognosis and lymph node metastasis. Alam et al.^51^ identified differentially expressed genes (DEGs) between TNBC samples and control samples, in which ACTB was significantly upregulated as a key gene. Vishnubalaji et al.^52^ proposed that targeted depletion of ENO1 halted TNBC cell proliferation, colony formation, and organoid tumor growth under 3-dimensional conditions and increased cell death, suggesting their potential use as novel therapeutic targets for TNBC. Reaves et al.^53^ demonstrated that Lipolysis Stimulated Lipoprotein Receptor (LSR) promotes collective cell migration and that overexpression of LSR alters the expression of genes involved in pathways involved in transformation and tumorigenesis. Wang et al.^54^ investigate the effect of the Tubulin beta class I gene (TUBB) on TNBC from the molecular perspective, proving that downregulated TUBB can dampen proliferation, migration and EMT, while facilitating apoptosis of TNBC cells. Huang et al.^55^ found that solute carrier family 7 member 5 (SLC7A5) plays a vital role in glutamine metabolism reprogramming in TNBC cells.

The top 10 important genes and their statistical information in the image-CAF model are presented in Supplementary Table S10, and their descriptions are provided in Supplementary Table S11. In the top 10 important genes in the image-CAF model, PRLR, IGF1R, ERBB3, and RPN2 have been confirmed to be related to TNBC^56–59^. ERBB3 and PRLR were significantly downregulated in the TNBC group, while TPM4 was significantly upregulated in the TNBC group in our DEGs results. The top 10 genes in the CAF and TNBC groups have one overlapping gene, CRABP2, which relates to breast cancer^60^, and all the other identified important genes are also shown to be related to breast cancer^49–59,61–67^. Through this gene importance analysis, our model identified key genes that play a central role in the pathogenesis of TNBC. This approach provides a powerful tool for uncovering the underlying biological and molecular mechanisms driving TNBC, offering valuable insights into its pathophysiology and potential therapeutic targets.

## Discussion

TME is essential for cancer development, and a major challenge in current cancer research and clinical practice is the accurate, non-invasive analysis of its immune components, which is crucial for developing personalized therapeutic strategies. In addition, understanding TME, especially the immune and mesenchymal components, is important for predicting disease progression and treatment response. Traditional methods often rely on invasive biopsies, which limit their applicability and patient comfort. Our study addresses this issue by correlating MRI with TME, thereby eliminating the need for invasive biopsies.

To ensure the acquisition of sufficient single-cell data for robust correlation with MRI data, we developed a computational deconvolution approach to obtain the relevant cellular components of the tumor immune microenvironment from bulk transcriptomic data. Our deconvoluted results find that the TNBC group has a higher proportion of CD4^+^ T cells, macrophages, and endothelial cells, which is consistent with the current research^26–29^. More interestingly, our novel finding reveals an inverse relationship between the proportion of CAFs and the level of T-cell infiltration, elucidating the interplay between CAFs and tumor-infiltrating T cells, highlighting mechanisms that may influence immune evasion and therapeutic resistance. Both our experimental findings and biological analyses demonstrated the robustness of the deconvolution method in handling tumor heterogeneity, allowing researchers and clinicians to derive new insights without the need for additional invasive biopsies.

We integrated genomic and image data to construct a suite of radiogenomics models, identifying the association between images and immune microenvironments and subtypes. Our ablation study, which involved adding and removing genomic data, demonstrated that our models achieve deep integration and complementarity between gene expression and MRI characteristics, enabling the identification of genomic properties through medical images (as presented in Supplementary Fig. S8). We successfully identified robust immune microenvironment imaging markers, specifically the CAF markers. However, due to the significant imbalance in the data, we were unable to obtain a stable biomarker for TNBC. The CAF markers provided promising support for tumor noninvasive subtyping as well as immunotherapy response prediction. Different cell types exhibit distinct MRI signal characteristics^68,69^. CAFs are often associated with more prominent fibrotic structures within the tumor microenvironment, which might be more readily captured on MRI compared to the dispersed infiltration of immune cells, which could partly explain the superior predictive performance for CAFs over other immune cell types. More importantly, our method is a general unsupervised lesion annotation method based on CAF markers that incorporates immune microenvironment information.

To enhance the interpretability of TME and provide clinicians with actionable insights derived from imaging, we further investigated the association between radiomic features and TME and constructed an interpretable model to perform non-invasive TNBC detection. Among the significant features, SumAverage and Sphericity of the marker region show higher values in the TNBC group, while MaximumDiameter, Skewness, InterquartileRange, and Entropy of the marker region values show lower values in the TNBC group. The consistent distribution across multiple independent datasets indicated a strong association between these radiomic features and cancer subtypes or specific biological processes. Moreover, there are some studies to correlate the radiomic features with BI-RADS^45–47^, especially Kurtosis and Skewness^46^. Entropy measures the randomness or disorder of a texture. Higher entropy in malignant lesions correlates with a more disorganized tissue architecture, which may underpin descriptors such as “irregular margins” or “non-mass enhancement” in BI-RADS terms.

Finally, we analyzed the significant genes, identified through our deconvolution method, that contributed to TNBC and CAF by interpreting our radiogenomic models. In the top 10 important genes in the image-TNBC model, the 7 genes, including KRT8, KRT18, CCT6A, ACTB, ENO1, TUBB and SLC7A5, have already been confirmed as related to the TNBC subtype. CRABP2, 100A11 and LSR have not been verified. Feng et al. suggested that CRABP2 can suppress the invasion and metastasis of ER+ breast cancer and promote invasion and metastasis of ER- breast cancer^60^. S100A11 is a previously unexplored factor in macrophage-cancer crosstalk, predicts high macrophage density and poor outcomes in ER+ tumors^67^. Denise K. Reaves et al. showed that LSR may promote collective cell migration and that overexpression of LSR altered gene expression in pathways involved in transformation and tumorigenesis^53^. The interpretability of our framework advances marker gene discovery and clinical practice.

In conclusion, our DiRG modeling workflow offers a promising approach to non-invasive, precision cancer subtyping by correlating MRI with TME. The identification of CAF-associated imaging biomarkers further underscored the significance of our approach in terms of biological relevance and clinical potential. It can act as an effective tool for early cancer screening and is expected to change how cancers are diagnosed and treated through personalized medicine based on a deeper understanding of TME. In addition, the deconvolution method, deep radiogenomic models to identify imaging biomarkers, and the unsupervised lesion annotation method using CAF markers and immune microenvironment data are designed as general approaches. These methods are not limited to the specific context of this study and can be applied independently to other datasets or research contexts.

Collectively, this study establishes a comprehensive radiogenomic framework that bridges MRI phenotypes with tumor microenvironment (TME) biology, offering clinically interpretable biomarkers for precision oncology. Our work makes four key contributions:

1. We developed three generalizable computational tools–a tumor heterogeneity-aware deconvolution method for robust TME estimation from bulk transcriptomics, a suite of deep radiogenomic models for imaging-biomarker discovery, and an unsupervised lesion-annotation framework that integrates immune microenvironment information to enhance interpretability.
2. To our knowledge, this is the first study to identify robust cancer-associated fibroblast (CAF) markers from breast MRI. By extracting CAF-related radiomic features, we demonstrated that shape and texture features (e.g., Sphericity, Entropy) can serve as non-invasive surrogates for CAF abundance and TNBC subtyping.
3. We uncovered a significant inverse correlation between CAF proportion and T-cell infiltration levels, providing new evidence for the immunosuppressive role of CAFs and their potential as therapeutic targets.
4. The identified radiomic features exhibited consistent distribution patterns in TNBC patients–elevated SumAverage and Sphericity, and reduced MaximumDiameter, Skewness, InterquartileRange, and Entropy–suggesting their utility for non-invasive TNBC subtyping and risk stratification.

By integrating interpretable machine learning with multi-omics data, this work advances the precision oncology paradigm and offers tools for early screening, treatment monitoring, and personalized therapy. The DiRG workflow can be adapted to other cancer types and imaging modalities, underscoring its broad applicability.

## Methods

### Ethical approval declarations

The single-cell cohort, radiogenomics discovery cohort, radiogenomics validation cohort, and radiomics cohort collected from TCGA, TCIA, and GEO belong to public databases. The patients involved in the database have obtained ethical approval, and users can download relevant data for free for research and publish relevant articles. Our study is based on open-source data, so there are no ethical issues or other conflicts of interest. As all data are open-source and de-identified, the need for additional ethical approval and informed consent was waived.

The research of the clinical cohort as a retrospective study was approved by the Institutional Review Board of Sun Yat-Sen University Cancer Center (approval number: B2025-202-01) and undertaken according to the Declaration of Helsinki, with a waiver of informed consent requirement for retrospective data analysis. However, written informed consent was specifically obtained from patients who received the treatment and underwent imaging examinations as part of their clinical care.

### Cohorts and datasets

The study contained five cohorts: the single-cell cohort, the radiogenomics discovery cohort, the radiogenomics validation cohort, the radiomics cohort, and the clinical cohort, as shown in Fig. 1D.

We obtained the single-cell cohort from Wu. et al.^22^ and GSE136831^23^, a total of 84 samples with 337,200 cells. The Wu dataset contained 24,272 cells with 20 cell types from five breast cancer samples. GSE136831 contains 312,928 cells from 32 Idiopathic Pulmonary Fibrosis (IPF), 29 healthy controls and 18 chronic obstructive pulmonary disease (COPD) lungs. The single-cell data of the Wu dataset are downloaded from the single-cell portal (https://singlecell.broadinstitute.org/single_cell/study/SCP1039/). GSE136831 is downloaded from Gene Expression Omnibus (GEO, https://www.ncbi.nlm.nih.gov/geo/query/acc.cgi?acc=GSE136831). Based on single-cell data, we construct the pseudo-bulk data by summing all single-cell data in each cell type for each sample to validate the effectiveness of our deconvolution method.

We enrolled a radiogenomics cohort of 117 breast cancer patients from The Cancer Genome Atlas (TCGA), containing gene and image data. All 117 breast cancer patients were partitioned into a radiogenomics discovery cohort (*n* = 88) and a radiogenomics validation cohort (*n* = 29). In the TCGA-BRCA dataset, samples with matched imaging and genomics data were included in the radiogenomics cohort, obtaining 137 samples. Referring to TCIA (The Cancer Imaging Archive, available at: https://www.cancerimagingarchive.net/analysis-result/tcga-breast-radiogenomic/), we included only breast MRI studies acquired on GE 1.5 Tesla magnet strength scanners (GE Medical Systems, Milwaukee, Wisconsin, USA), yielding a total of 93 cases. Subsequently, we excluded cases that did not have images in the dynamic sequence or did not have gene expression analysis available through the TCGA Data Portal at the time of our review. Additionally, we excluded patients with normal subtypes. This process ultimately yielded a total of 88 patients. To validate the generalizability of the model, we established a radiogenomics validation cohort. Unlike the radiogenomics discovery cohort, this new cohort includes data that were not acquired using GE 1.5 Tesla magnet strength scanners, while maintaining all other exclusion criteria consistent. We obtained 29 patients in total. The sample IDs of the radiogenomics cohort are listed in Supplementary Table S12.

We also constructed a radiomics cohort derived from ISPY1 (*n* = 81) and Breast-MRI-NACT-Pilot (NACT-Pilot) (*n* = 39). The ISPY dataset provides expert annotations and radiomic features for the ISPY1/ACRIN 6657 trial data collection, which includes multicenter breast DCE-MRI scans^70^. For the ISPY dataset, in the matched Dynamic Contrast-Enhanced Magnetic Resonance Imaging (DCE-MRI) sample data, we randomly selected 81 cases as research subjects. For each patient, we selected image samples with a total pixel value of more than 1500 in their tumor label images to ensure the representativeness of the tumor data. The NACT-Pilot study was designed to investigate the use of serial DCE-MRI examinations during neoadjuvant chemotherapy for invasive breast cancer. This study recruited 68 patients diagnosed with stage II or III locally advanced breast cancer. We selected samples that contained tumor annotation labels and similarly included only those samples whose tumor label images had a total pixel value exceeding 1500. We obtained a total of 465 images from 39 patients. Selected samples are described in Supplementary Table S12.

In the clinical cohort, we enrolled 237 patients from Sun Yat-Sen University Cancer Center (SYSUCC), which contained 96 TNBC patients and 141 non-TNBC patients from October 2018 to December 2024.

In summary, there are 88 patients with 565 medical images in the radiogenomics discovery cohort and 29 patients with 188 images in the radiogenomics validation cohort. For the radiomics cohort, 81 patients with 1329 images in the ISPY1 dataset and 39 patients with 465 images in the NACT-Pilot dataset. There are 237 patients with 1614 images in the clinical cohort.

The radiogenomics discovery cohort, it contained four subtypes of breast cancer: luminal A (LumA), luminal B (LumB), HER2-enriched (HER2-E), and TNBC. We divided these subtypes into the TNBC group (*n* = 12) and the non-TNBC group (*n* = 76). The radiogenomics validation cohort (*n* = 29) contains 4 TNBC patients and 25 non-TNBC patients. In the radiomics cohort (*n* = 120), the ISPY (*n* = 81) contains 20 TNBC patients and 61 non-TNBC patients, and the NACT-Pilot (*n* = 39) contained 11 TNBC patients and 28 non-TNBC patients. The image data of TCGA-BRCA, ISPY1, and NACT-Pilot were obtained from TCIA. The clinical and transcriptomic data of TCGA-BRCA were procured from the GDC data portal (https://portal.gdc.cancer.gov). The cancer subtype of each patient in the radiogenomics discovery cohort and radiogenomics validation cohort was obtained from the research^71^. In the radiomics cohort, the subtype labels of the patients were derived from the clinical information of the images. The Estrogen receptor (ER), Progesterone receptor (PR), and Human epidermal growth factor 2 (HER2) information of the patients was obtained through the Overall Assessment section in the Standard Report files (SR) in TCIA, and the patients were subtyped by clinical criteria, which were referenced as shown in Table 5. The ER is the estrogen receptor, and excess estrogen can be a pathogen, carcinogenic to the breast epithelium. If the ER is positive, it can inhibit tumor growth by preventing the cancer cells from receiving estrogen signals, causing the tumor’s death. The PR is the progesterone receptor, and PR positivity is essentially similar in significance to ER positivity. HER2 is a proto-oncogene, and patients with HER2 overexpression have more aggressive tumors, higher malignancy, and faster progression; are more likely to recur and metastasize; and have a poorer prognosis.

**Table 5:** Relevant clinical criteria for determining subtypes of breast cancer

Subtype	HER2	ER	PR
HER-2 positive	Positive	Negative	Negative
HER-2 negative	Positive	Positive	Any
Triple Negative Breast Cancer	Negative	Negative	Negative
Luminal A	Negative	Positive	Positive
Luminal B	Negative	Positive	Negative

In the clinical cohort, based on surgical resection and biopsy, we obtained information on cancer subtypes. The original pathology reports were retrospectively reviewed. Histological reports were routinely issued by two board-certified pathologists. Tumor histology and immunohistochemical profiles, including estrogen receptor (ER), progesterone receptor (PR), and HER2 status, were obtained from final histopathological assessments of surgical tumor specimens or core needle biopsy samples. ER, PR and HER-2 status evaluations adhered to the College of American Pathologists Clinical Practice Guidelines^72^. Estrogen or progesterone receptor-positive tumors with over 1% staining were classified as hormone receptor-positive. Triple-negative (TN) status was defined as hormone receptor- and HER2-negative^73^. Lesions with equivocal HER2 status underwent additional verification through fluorescence in situ hybridization, with positivity defined by confirmed gene amplification.

### Imaging protocol

For the radiogenomics discovery and validation cohorts, the DCE-MRI protocol was described elsewhere^74^. The DCE-MRI data were acquired with the patients using a T1-weighted gradient echo sequence. The in-place resolution ranged from 0.53 to 0.85 mm, the slice thickness ranged from 2 to 3 mm, and the flip angle was 10°. For each patient, select 6 to 8 matching images containing tumors as input.

For the radiomics cohort, the DCE-MRI protocol was described in the research of TCIA (https://www.cancerimagingarchive.net/collection/breast-mri-nact-pilot/) and elsewhere^70^. The DCE-MRI of ISPY was acquired sagittally at 1.5-T using T1-weighted fat-suppressed sequences and a breast coil. Parameters included: TR less than or equal to 20 ms, TE equal to 4.5 ms, flip angle less than or equal to 45°, field of view, 160-180 mm, matrix 256 × 192, 64 slices, slice thickness less than or equal to 2.5 mm, in-plane resolution less than or equal to 1 mm. The DCE-MRI of NACT-Pilot was acquired with the patients placed in the prone position using a 1.5-T MRI (GE Healthcare, Milwaukee, Wis). T1-weighted fat-suppressed MRI was performed under the following parameters: TR, 8 ms; TE, 4.2 ms; matrix, 256 × 192 × 60; flip angle, 20°; field of view, 180–220 mm; in-plane resolution, 0.7–0.9 mm; and slice thickness, 2–2.4 mm.

For the clinical cohort, all MRI examinations at Sun Yat-sen University Cancer Center were conducted using 3 Tesla scanners (GE Healthcare, Milwaukee, Wisconsin, USA). A body coil served as the transmitter, and an 8-channel phased-array breast coil (GE Healthcare) was utilized for signal reception. Patients were positioned prone with breasts immobilized in a dedicated holder to minimize motion artifacts. The imaging protocol included T1-weighted imaging (T1WI), T2-weighted imaging (T2WI), and contrast-enhanced T1-weighted imaging (T1+C). Following the acquisition of T1WI and T2WI sequences, a gadolinium-based contrast agent, either gadoteric acid meglumine (Jiadixian, Hengrui Healthcare, China) or gadobenate dimeglumine (MultiHance, Bracco Healthcare, China), was administered intravenously via an MRI-compatible automatic power injector (Medrad Inc.) at a flow rate of 3 mL/s and a dose of 0.2 mL/kg body weight. A 20 mL saline flush (3 mL/s) was delivered to clear the intravenous line. Dynamic scan was initiated by pressing the high-pressure syringe button and the dynamic scan button simultaneously, followed by acquisition of axial T1+C images. Subsequently, sagittal fat-suppressed T1+C images were obtained with the following parameters: TR/TE is 7.1/1.9 ms, field of view (FOV) is 28 cm, matrix size is 256 × 256, flip angle is 12°, slice thickness is 3.0 mm, and acquisition time is 1 minute.

### Data processing and radiomic feature extraction

The original gene expression count data for BRCA were obtained from the TCGA dataset, which initially covered 60,660 genes. To improve the efficiency and relevance of the data analysis, we first pre-processed the data by removing genes with zero expression in more than 90% of all samples. The number of genes retained was finally reduced to 22,781. The 22,781 genes were employed as the genomic features of the sample and were input into the iBayesPrism model for deconvolution. The deconvoluted gene expression, comprising 12,966 genes, was used as input to the radiogenomics models.

The MRI data were converted and resized using Python. From TCIA, we obtained the DICOM files of each patient and converted them into readable files (JPG or PNG files) based on the Pydicom package V2.4.4, implemented in Python 3.8^75^. DCE-MRI data were resized to the same resolution and 224*224 size before feature extraction.

Based on the microenvironment or subtype annotations acquired with Grad-CAM, we performed radiomic feature extraction using the open-source Pyradiomics package V3.0.1, implemented in Python 3.10^76^. The image radiomic features were extracted according to Fan et al. ^5^. The final 36 image features were extracted as some of the features have been discarded, and the related descriptions of the 36 features are shown in Table 6. First-order statistics describe the distribution of voxel intensities within the image region defined by the mask using commonly employed basic metrics. In the Shape2D group of features, we included descriptors of the two-dimensional size and shape of the ROI. Gray Level Co-occurrence Matrix (GLCM) is a statistically based method for texture feature extraction that reflects the joint gray-level distribution and correlation of pixel pairs in an image.

**Table 6:** The description of the radiomic feature

Feature	Number of features	Feature description
First order	10	Mean, Standard Deviation, Kurtosis, Skewness, Maximum, Minimum, Median, Interquartile Range, Range, Entropy
Shape2D	10	MeshSurface, PixelSurface, Perimeter, PerimeterSurfaceRatio, Sphericity, SphericalDisproportion, MaximumDiameter, MajorAxisLength, MinorAxisLength, Elongation
GLCM	16	Joint Energy, Contrast, Correlation, Sum of Squares Variance, Sum Average, Joint Entropy, Difference Variance, Difference Entropy, Difference Average, Information Correlation 1, Information Correlation 2, Autocorrelation, Cluster Shade, Cluster Prominence, Maximum Probability, Inverse Difference

### TME inference module

We developed the deconvolution method based on the BayesPrism^15^ method and a customized reference matrix.

To adapt to tumor heterogeneity, we select BayesPrism as our deconvolution method. BayesPrism is a Bayesian model that predicts the cellular composition and gene expression of individual cell types from bulk RNA-seqs, with patient-derived scRNA-seq reference used as prior information. The reference matrix contained the cell states and cell types. Bayesprism adjusts the reference matrix to fit the bulk expression in each iteration, which makes it suitable for highly heterogeneous samples with complex expression patterns.

BayesPrism consists of four major steps:

1. Utilizing the single-cell reference matrix *φ* and the input bulk expression *X*_*n*_ of the *n*th bulk sample as the prior distribution and the sample information, respectively, the Gibbs sampling method was employed to infer the joint posterior distribution *P*(*μ*_*n*_, *U*_*n*_∣*φ*, *X*_*n*_) of the proportion of cell states *μ*_*n*_ and gene expression *U*_*n*_ of the *n*th bulk sample. The reference matrix *φ* is generated by summing the count matrices for each cell state of the input original reference matrix and then normalized by the total counts.
2. For each bulk sample *n*, BayesPrism estimates the gene expression matrix *U*_*n*_ and the proportion *μ*_*n*_, for each cell state, by marginalizing the joint posterior.
3. For each bulk sample *n*, BayesPrism estimates the gene expression matrix *Z*_*n*_ and the proportion ${\theta }_{{0}_{n}}$, for each cell type, by summing the posteriors over cell states (estimated in step 2) within each cell type.
4. BayesPrism can update the reference matrix *φ* to obtain a new reference matrix *ψ* by pooling the information across bulk samples from Z. BayesPrism will re-estimate the marginal posterior of the cell-type fraction for each of the bulk samples using the latest prior distribution parameterized by *ψ*, that is, $P({\theta }_{{f}_{n}}| \psi ,{X}_{n})$. In the description above, ${\theta }_{{0}_{n}}$ is the initial cell-type fractions of the *n*th sample, ${\theta }_{{f}_{n}}$ is the updated cell-type fractions of the *n*th sample.

Our main goal is to correlate medical images with TME; some rare cell types are unnecessary and could induce noise during deconvolution. We utilized scATOMIC^16^, a single-cell annotation tool of TMEs, to customize the reference matrix specifically for our cell types of interest.

The scATOMIC method is a modular annotation tool for malignant and non-malignant cells. Compared with other tools, scATOMIC has advantages in cancer environments as it can accurately identify TME-resident cells at high resolution, distinguish cancer and normal tissue cells, and determine tumor origin, which will enrich and facilitate a wide range of cancer research. The scATOMIC model was trained on more than 300,000 cancer, immune, and stromal cells, defining a pan-cancer reference across 19 common cancers. The scATOMIC method structured a pan-cancer TME cellular hierarchy where each parent node represents a group of related cells, and each terminal node represents a single-cell class of interest, as shown in Supplementary Fig. S1. In the hierarchical structure of scATOMIC, the cells are subdivided from top to bottom, with the cell type at the upper level (parent nodes) containing more information, e.g., stromal cells are divided into endothelial cells and fibroblast cells. Based on scATOMIC methods, we can obtain a pan-cancer reference matrix of 19 cancer cells and 20 non-cancer cells (blood cells and stromal cells).

To obtain a reliable reference matrix, we first constructed a customized pan-cancer reference matrix that contains 20 non-cancer cells and breast cancer cells to deconvolute the bulk data of the radiogenomics discovery cohort, as shown in Fig. 1C. With the deconvolution results of the customized reference cell types, we updated the reference matrix. We finally selected cellular components that accounted for a larger portion of the initial deconvolution and included subnode cell types that could no longer be subdivided within the scATOMIC architecture. Ultimately, this process allowed us to derive the gene expression profiles and proportions for eight key cell types: B cells, CD4^+^ T cells, CD8^+^ T cells, CAFs, fibroblasts, breast cancer, macrophages and endothelial cells.

### Compare with other deconvolution methods

We compared our method with reference-based methods (Bisque, CIBERSORT, quanTIseq, EPIC) and a gene signature-based enrichment method (xCell).

Bisque^21^ implements a regression-based approach that utilizes single-cell RNA-seq data to generate a reference expression profile and learn gene-specific bulk expression transformations to decompose bulk RNA-seq data robustly. We employed the 20 non-cancer cells from scATOMIC as a reference matrix, and performed the Bisque method using the “BisqueRNA” package in R.

CIBERSORT^12^ is an SVM regression-based method, which deconvolves the expression matrix of immune cell subtypes based on cell type reference expression profiles to estimate the abundance of immune cells. CIBERSORT provides expression data LM22 for 22 common immune infiltrating cells, including immune cells of different cell types and functional states. We employed the LM22 as a reference matrix and performed CIBERSORT using the “CIBERSORT” package in R.

The quanTIseq^24^ is used to quantify tumor immune status based on human RNA-seq data, quantifying the proportion of ten different immune cell types present in the reference sample and the proportion of other uncharacterized cells by deconvolution. The ten cell types include B cells, Macrophages M1, Macrophages M2, Monocytes, Neutrophils, NK cells, CD4+ T cells, CD8+ T cells, Dendritic cells and Other uncharacterized cells. We performed the quanTIseq method using the “immuneconv” package in R, and referenced the fixed reference matrix in quanTIseq.

The xCell^13^ is a signature-based method, and it can be used to infer 64 immune and stromal cell types. The xCell compendium of newly generated gene signatures for 64 cell types, spanning multiple adaptive and innate immunity cells, hematopoietic progenitors, epithelial cells, and extracellular matrix cells, was derived from thousands of expression profiles. The xCell method is a computational-based method that extracts expression signatures of 64 immune and stromal cells as cell signatures from bulk gene expression data. Then, the enrichment scores of the samples on each cell type signature were calculated using ssGSEA^77^. The enrichment scores for each cell type were converted to the corresponding cell type scores using the fitting equation. We performed the xCell method using the “immuneconv” package in R.

EPIC^25^ uses constrained least squares regression to incorporate non-negativity constraints into the deconvolution problem to estimate the proportions of immune and cancerous cells from a large amount of tumor gene expression data. EPIC analyses the proportion of infiltration of eight types of immune cells, including B cells, tumor-associated fibroblasts (CAFs), CD4^+^ T cells, CD8^+^ T cells, endothelial cells, macrophages and NK cells, based on the expression data. We performed the EPIC method using the “immuneconv” package in R.

### Models predicting TME and cancer subtypes

We utilized a deep learning module for extracting image features and a separate module for genomic feature extraction and significance analysis. After obtaining both the gene feature vectors and image feature vectors, we combined and fed them into a three-layer fully connected neural network to perform the final classification. This integrated approach is depicted in Supplementary Fig. S7(A). For imaging data, we employed a U-Net module with an encoder-decoder structure (encoder channels: [32, 64, 128, 256]; bottleneck: [256, 512]; decoder channels: [256, 128, 64, 32]). The extracted image features were processed through three fully connected (FC) layers (5120, 1024, 512). For genomic data, the SMLP module with three FC layers (10240, 1024, 512) was used. The resulting 1024-dimensional concatenated features (image features with gene features) were fed into a three-layer classifier (128, 32, 2). All models were trained and evaluated using a five-fold cross-validation strategy. To identify imaging regions associated with the TME, we applied Grad-CAM to generate attention maps. A threshold was applied to convert attention scores into discrete regions of interest. We systematically evaluated thresholds of [0.5, 0.6, 0.7] on the discovery cohort, finding that retaining the top 60% of attention scores yielded optimal performance, as shown in Supplementary Table S4. From the CAF-associated ROIs, we extracted 36 radiomic features. To build a non-invasive classifier for distinguishing TNBC from non-TNBC groups, we compared multiple machine learning models–Support Vector Machine (SVM), Random Forest (RF), and eXtreme Gradient Boosting (XGBoost)–across all datasets. XGBoost achieved the best performance. This model was trained with an 8:2 train-test split using its default initial parameters.

### Deep radiomic feature extraction

To extract different image features, we employed UNet^17^ to perform feature extraction. UNet is suitable for different types of image segmentation tasks, and it can be trained end-to-end from very few images. In this paper, the UNet model consists of four down-sampled blocks (encoder module) and four up-sampled blocks (decoder module); each block contains two convolutional layers, and one bottleneck layer to connect the up-sampled and down-sampled layers (Supplementary Fig. S7(B)).

The encoder module consists of multiple convolutional blocks, each of which includes convolutional layers (3*3 convolutional kernels), batch Normalization, and activation functions (ReLU), which allow the network to learn the low-level features of the image. In this study, each convolutional block contains two identical convolutional layers. The ReLU function is represented as *f*(*x*) = *max*(0, *x*). Following each convolutional block, a pooling layer (e.g., maximum pooling or average pooling) is added to reduce the size of the feature map while retaining important information. At the top of the encoder, a bottleneck layer exists that connects the encoder to the corresponding layer of the decoder and helps restore the lost spatial information in the decoder.

The decoder consists of multiple deconvolution blocks, each containing the deconvolution layer (also known as transposed convolution), batch normalization, and the activation function, which helps to learn high-level semantic information about the image. Following each deconvolution block, the upsampling layer is added to increase the size of the feature map, corresponding to the pooling layer in the encoder. UNet also introduces skip connections, where the feature maps obtained from each downsampling layer in the encoder are combined with the feature maps obtained from the corresponding decoder layer. The skip connections allow the model to retain more low-level, high-resolution information, which improves the model’s ability to capture localized information like details and edges.

Following the UNet module, three fully connected layers are employed for feature downsampling. A Tanh activation function is applied after each fully connected layer. Tanh function is represented as $\frac{{e}^{x}-{e}^{-x}}{{e}^{x}+{e}^{-x}}$. The resulting vector from these layers serves as the processed input image features for subsequent steps.

### Genomic feature extraction and significance analysis

We provide a module for gene feature extraction and significance analysis by utilizing the MLP with a pre-single connection layer (SMLP) architecture^18^. The module consists of a pre-single connected layer followed by three fully-connected modules (supplementary Fig. S7(C)). The first two fully-connected modules each include a fully-connected layer with Batch Normalization and ReLU activation functions. The final output layer is a fully-connected layer with a Sigmoid activation function. The Sigmoid function, represented as $\frac{1}{1+{e}^{-x}}$, normalizes the output of the neural network by mapping the output values to the range [0, 1].

The SMLP introduces a single-to-single connection layer with a ReLU activation function before the original MLP. This additional layer is designed to evaluate the importance of each dimensional feature in the input features of the MLP layer. In the pre-single connection layer, the parameters of all input layers are initially preset to 1. This setting constructs an equal starting point, meaning that all input features are considered to have the same importance during the initial stages of model training. As the model training process progresses, the parameters of the pre-single connection layer are updated and adjusted. Based on optimization techniques such as the back-propagation algorithm and gradient descent, the parameters of the pre-single connection layer can capture the contribution of different input features to the prediction results. Specifically, the parameters corresponding to those features that have a significant impact on the prediction results will be given a larger weight, while the features with a smaller contribution may be given a smaller weight, or even in some cases, the weight may be close to zero, thus realizing a dynamic assessment of the importance of the features.

The SMLP is a novel feature importance measure as well as a feature screening method for neural networks. The pre-single connection layer provides the MLP with an additional layer to capture the complex relationships between the input features and also enables fine-grained control of the different neuron inputs through a parameter update mechanism. This mechanism allows the model to focus more accurately on those features that are critical to the prediction results, thus improving the predictive performance and generalization of the model.

### Predict TME and cancer subtypes

Features from the two different sources (image and genome) are concatenated to form a combined feature vector. This integrated feature vector is then fed into a three-layer fully connected neural network to perform the final classification prediction. The entire model leverages the unique information from both image and genomic data, enhancing prediction accuracy and the generalization ability of the model by combining multi-modal feature integration with deep learning algorithms.

We categorized the TME cell proportions, obtained from the deconvolution step, into TME labels–low and high groups. These TME labels, along with subtype labels, are used to train the models to predict both TME categories and subtypes. The loss is calculated with the CrossEntropyLoss function as follows:

1 $$L=-\mathop{\sum }\limits_{x}p(x)logq(x),$$

where the probability distribution p is the desired output and the probability distribution *q* is the actual output.

Based on five-fold cross-validation, we divided the dataset into five parts, with four parts used for training the model and the remaining one part used for testing each time. This approach ensures that the model learns the overall distribution of the data while also testing its generalization ability on new data, thereby effectively reducing the risk of overfitting. The Stochastic Gradient Descent (SGD) algorithm with impulse is applied to train the network. Accordingly, we use a high momentum (0.9) such that a large number of the previously seen training samples determine the update in the current optimization step. For the whole model, we have employed PyTorch 2.2.0 as the implementation tool for the DL framework (https://pytorch.org/) and combined it with Python 3.10.1 (https://www.python.org/).

### Surrogate model for non-invasive subtyping

Utilizing interpretable surrogate models can directly classify breast cancer subtypes based on extracted radiomic features. We selected eXtreme Gradient Boosting (XGBoost) as our final model based on a systematic comparison with other machine learning models–Support Vector Machine (SVM) and Random Forest (RF). To train and test the SVM, RF and XGBoost on all datasets, the results are shown in Table 2 and Fig. 5E. Across all five validation datasets, XGBoost demonstrated superior or consistently competitive performance in key metrics, particularly in PR-AUC and sensitivity. This indicates its strong capability to handle potential class imbalance and deliver robust classification. XGBoost employs an additive modeling strategy, constructing a final strong learner by sequentially adding weak learners (decision trees). The prediction of XGBoost can be expressed as the weighted sum of multiple weak learners:

2 $$\widehat{y}=-\mathop{\sum }\limits_{i}^{k}{f}_{i}(x),$$

where *K* is the number of weak learners, and *f*_*i*_(*x*) is the prediction of the *i-th* weak learner for sample x. During training, each new weak learner is added to minimize the current model’s loss. XGBoost determines the structure and parameters of a new weak learner by computing the loss function of the gradient and the second-order derivative of the current model’s predictions. The model was adjusted along the direction of the steepest descent of the loss function, progressively reducing the discrepancy between the predicted and true values.

For class imbalance, it can improve the effectiveness of the model through three complementary strategies: data-level resampling (e.g., SMOTE), algorithm-level cost-sensitive learning (e.g., class weight adjustment), and post-hoc threshold optimization on the validation set. XGBoost has the same parameter adjustment strategies for unbalanced data. To handle class imbalance, we employed several XGBoost-specific strategies. First, we set the *scale*_*pos*_*weight* parameter to balance positive and negative class contributions, using the ratio of negative to positive samples in the training data. Second, to reduce overfitting and improve cross-cohort generalizability, we applied subsampling via “subsample” (fraction of training instances used per tree) and *colsample*_*bytree* (fraction of features used per tree). Moreover, we adjusted the learning rate (eta) to control the step size of each boosting iteration and used early stopping with a validation set to halt training when performance ceased to improve. These parameter choices collectively enhanced sensitivity while preserving high specificity across all cohorts.

### Significant MRI biomarker identification and lesion annotation

After training the model, visualizing the attentional areas of the model by Gradient-Weighted Class Activation Mapping (Grad-CAM), we thus obtained the image information most relevant to the subtypes and immune environment. Grad-CAM^19^ is a method for visualizing the prediction process of a deep network, using gradient information to generate heat maps showing the regions of interest of the network. This technique accurately identifies crucial features and regions in the decision-making process of DL models, providing a powerful tool for model interpretability.

The main concept of Grad-CAM is to multiply the gradient information of the convolutional neural network (CNN) with the weights of the global pooling layer to obtain the importance of each spatial location for a specific class. Convolutional layers naturally retain spatial information, which is lost in fully-connected layers. These important weights are used to generate class activation mappings that reveal the image regions the network focuses on while making predictions. The process to obtain the important feature map ${L}_{Grad-CAM}^{c}$ for the specific class *c* involves the following steps: (1) First, forward the input image through the CNN module to obtain the task-specific class scores *y*^*c*^ (before softmax). (2) For the feature layer A to be visualized, compute the gradient $\frac{\partial {y}^{c}}{\partial {A}^{k}}$ with class *c*, in which *k* means the *k*th channel. (3) Adaptive average pooling is performed on the gradient to obtain the significant weights for each feature map channel, as shown as follows,

3 $${\alpha }_{k}^{c}=\frac{1}{Z}\mathop{\sum }\limits_{i}\mathop{\sum }\limits_{j}\frac{\partial {y}^{c}}{\partial {A}_{ij}^{k}},$$

where the $\frac{1}{Z}{\sum }_{i}{\sum }_{j}$ indicates the global average pooling, and *Z* is the multiplication of width *i* and height *j*. (4) Afterward, the feature maps and weights are multiplied channel-by-channel to obtain a weighted feature map for each channel, and then those weighted feature maps are summed channel-by-channel to obtain a two-dimensional heat map representing the importance of each pixel for the target class. ReLU is performed on the heat map to suppress the uninteresting parts of the weights. The whole Grad-CAM feature map for class *c* is shown as follows:

4 $${L}_{Grad-CAM}^{c}=ReLU(\mathop{\sum }\limits_{k}{\alpha }_{k}^{c}{A}^{k}).$$

To obtain the weighting parameters from Grad-CAM, we map the weighting parameters to the original image to visualize the attention area. Similarly, image annotations can be obtained from the extraction of weighting parameters. Based on a threshold to extract the related annotation and filter noise. We systematically evaluated thresholds of 0.5, 0.6, and 0.7 (corresponding to the upper 50%, 60%, and 70% of attention areas) on the radiogenomic discovery cohort. As shown in Supplementary Table S4, the 0.6 threshold achieved the optimal balance, delivering the best predictive performance, notably in ROC-AUC and PR-AUC. The upper 60% portion of the attention score is extracted and converted into lesion annotations, and some results are shown in Figs. 4 and 5.

Based on the trained models with the radiogenomics discovery dataset, we can directly obtain the relevant markers with Grad-CAM by presetting a certain class label. The well-trained architecture facilitates unsupervised segmentation of subtype or microenvironment markers directly from image data, eliminating the need for additional genomic input.

## Supplementary information

Supplementary Information

## Data Availability

The primary data supporting the results of this study are presented in the paper and its supplementary information. Single-cell data used for the deconvolution module are available in a single portal (https://singlecell.broadinstitute.org/single_cell/study/SCP1039/) and GEO (https://www.ncbi.nlm.nih.gov/geo/query/acc.cgi?acc=GSE136831). Transcriptomic data for the radiogenomics cohort were obtained from TCGA-BRCA (https://portal.gdc.cancer.gov/projects/TCGA-BRCA). MRI data for the radiogenomics cohort and radiomics cohort were obtained by TCIA (https://www.cancerimagingarchive.net/). Clinical data could not be shared due to patient confidentiality obligations. This study is based on Python and R for the main modeling and analysis. Custom code for visualization and data processing is available in GitHub (https://github.com/aI-area/Noninvasive-TME).
