# Supplementary material for: Deep interpretable radiogenomic workflow deciphers tumor microenvironment from breast MRI and identifies clinician-interpretable biomarkers
Source: NPJ Precis Oncol. 2026 May 6;10:316. doi: 10.1038/s41698-026-01458-2 (PMC13473565; doi:10.1038/s41698-026-01458-2)
Supplement: Supplementary file 1 [file 41698_2026_1458_MOESM1_ESM.pdf]

Supplementary Materials for

Deep Interpretable Radiogenomic Workflow Deciphers Tumor Microenvironment from Breast MRI and Identifies Clinician-Interpretable Biomarkers

Huijun Li *et al*.

Corresponding author: Chon Lok Lei, chonloklei@um.edu.mo; Peng Wang, ethanpwang@um.edu.mo; Hongyan Wu, hy.wu@siat.ac.cn

The PDF file includes:

Supplementary Text

Figures S1 to S16

Tables S1 to S12

References

Deconvolution method shows effectiveness in tumor heterogeneity

We employed scATOMIC method to construct our customized reference matrix, the scATOMIC based on random forest models to construct a pan-cancer TME cellular hierarchy, as shown in Fig. S1. The parent node in Fig. S1 represents a group of related cells, and each terminal node represents a single-cell class of interest.

To evaluate the model's effectiveness, we construct the pseudo-bulk data based on the single-cell data from the Wu dataset and GSE136831. We summed the single-cell data for each sample to construct pseudo-bulk data for the sample. The original distribution of single-cell data as the ground truth. After deconvolution, we can obtain the cell type proportion for each sample and select all overlapping cell types between original data and deconvolution results to perform analysis. Then, based on Mean Square Error (MSE), Pearson correlation, and cosine similarity, we compared the correlation of the deconvolution results with the original proportion.

In the Wu dataset, we employed all 20 non-cancer cells from scATOMIC as a reference matrix to perform deconvolution. There are seven overlapping cell types: B cells, cancer-associated fibroblasts (CAFs), CD4+ T cells, CD8+ T cells, endothelial cells, natural killer cells, and plasmablast cells. The distribution of ground truth and deconvolution results in the Wu dataset is as shown in Fig. S2(A). The correlation of ground truth and deconvolution results as shown in Fig. S1, we can see that most of the deconvolution cell types have a high correlation with original data.

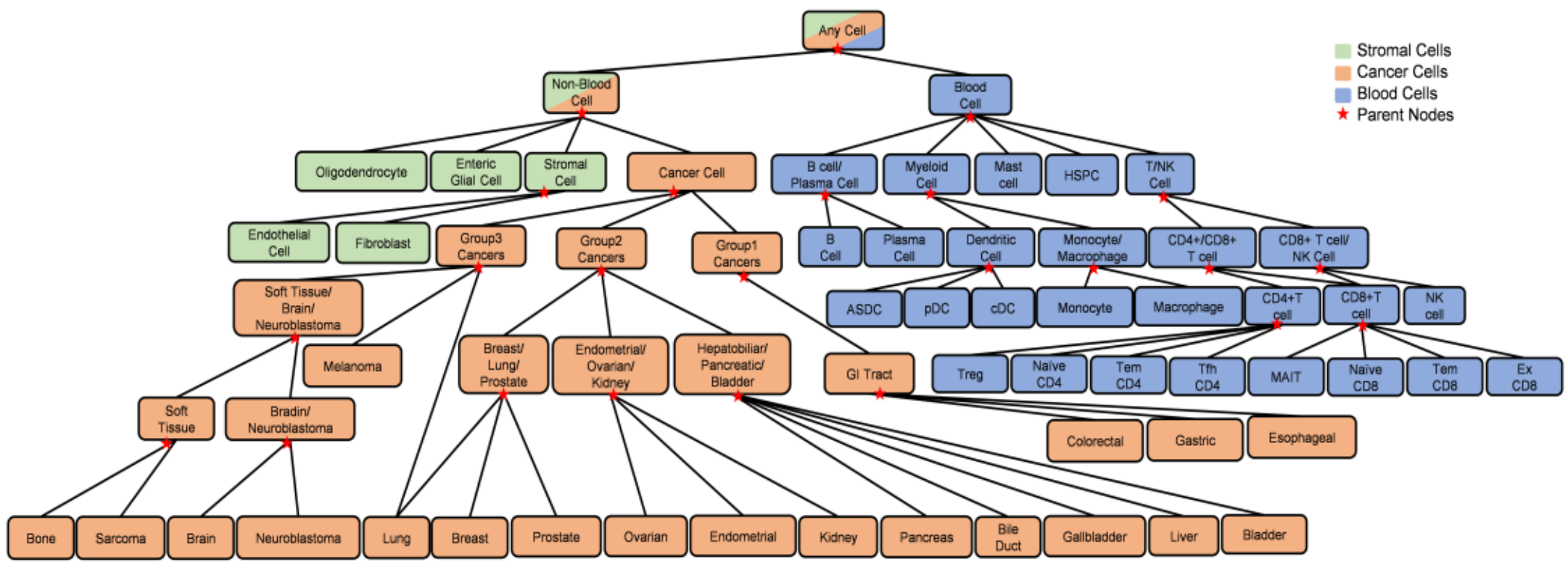

Fig. S1 Hierarchical structure of the pan-cancer tumor microenvironment.

In the same way, based on all 20 non-cancer cells from scATOMIC as a reference matrix, we deconvoluted the pseudo-bulk data of GSE136831. There are 12 cell types: B cell, dendritic cell, endothelial cell, fibroblast, macrophage, mast cell,

monocyte, myofibroblast, natural killer cell, plasmablast, smooth muscle cell, and T cell. The distributions of ground truth and deconvolution results in GSE136831 are shown in Fig. S2(B). The correlations of ground truth and deconvolution results of GSE136831 are shown in Table S1.

Table S1. The correlation of cell type fraction deconvoluted by our methods and ground truth in GSE136831.

	MSE	Pearson_Correlation	Cosine_Similarity
B cell	0.000887004	0.654996187	0.671188519
Dendritic	0.002525961	0.486816727	0.603237197
Endothelial Cells	0.003510885	0.862929914	0.594182924
Fibroblasts	0.000735085	0.500219645	0.562908043
Macrophage	0.10638135	0.547404807	0.907772721
Mast cell	0.000859179	0.002629611	0.088112036
Monocyte	0.010392363	0.3291497	0.617740066
Myofibroblasts	0.000741488	0.471917616	0.292984728
Natural killer cell	0.00060193	0.781700137	0.659218807
Plasmablast	2.76E-05	0.811382116	0.134226304
Smooth Muscle Cells	0.000214221	0.22157651	0.28986615
T cell	0.01113646	0.9243514	0.530935203

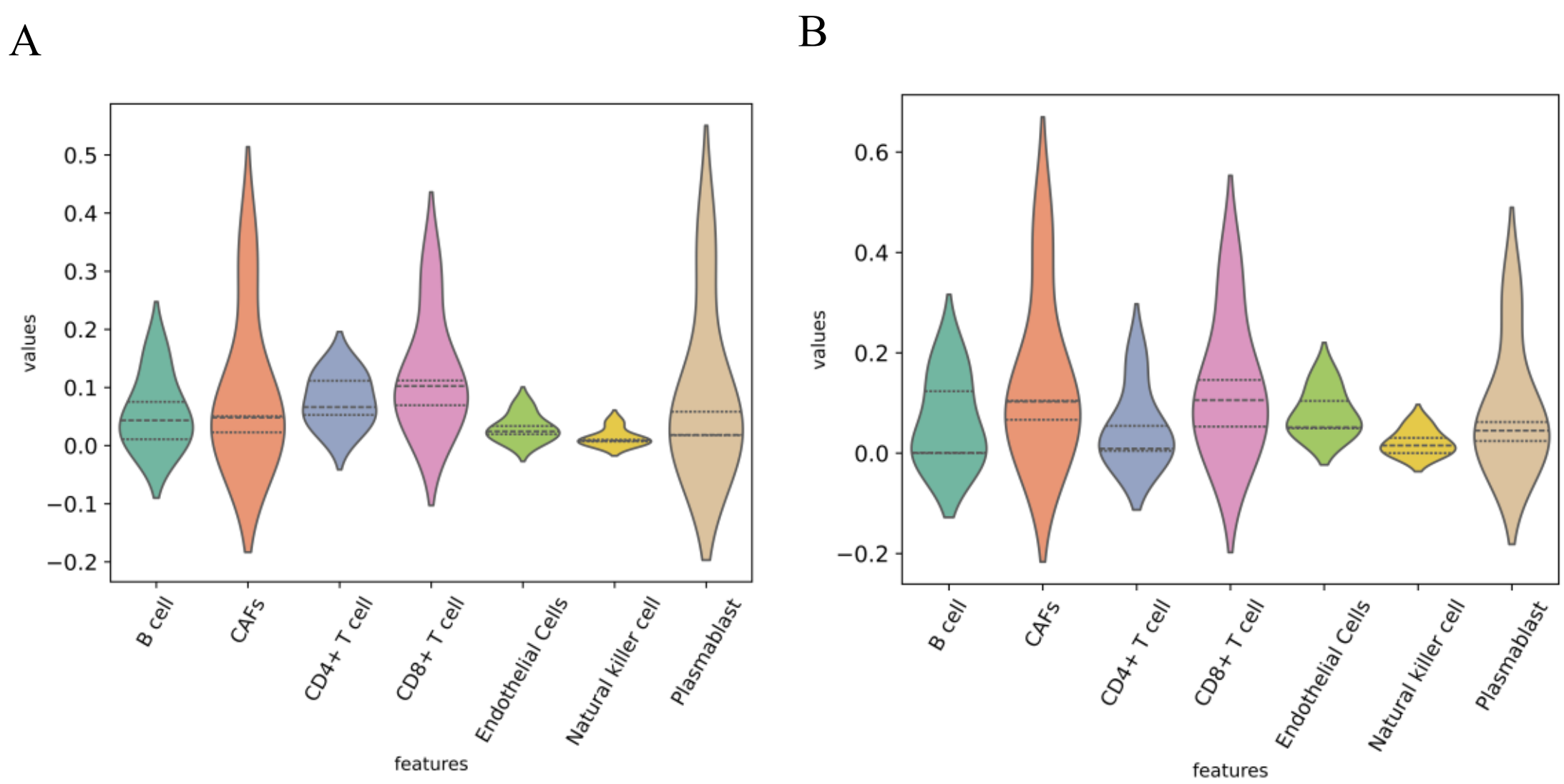

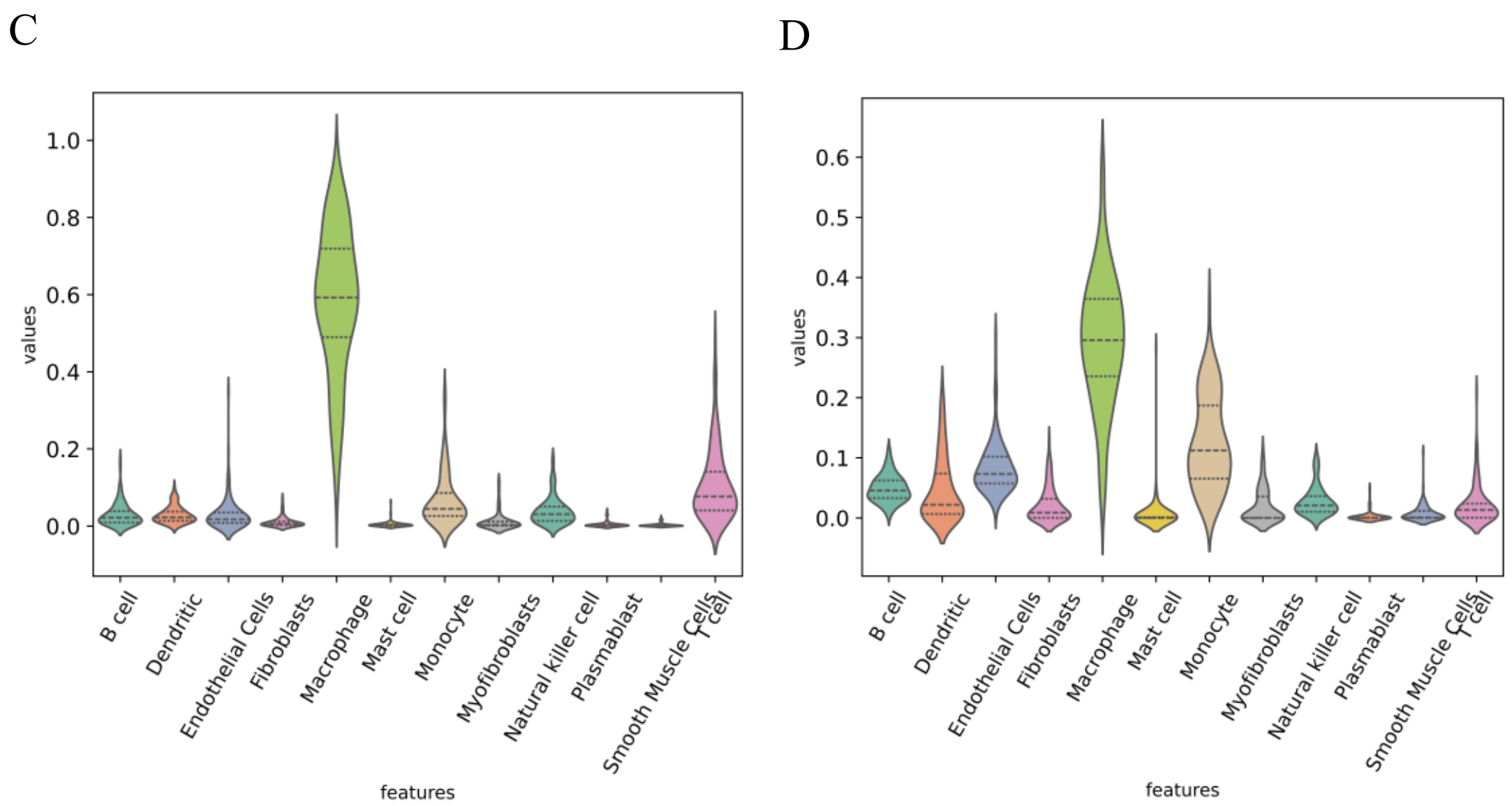

Fig. S2. The distribution of the cell type proportions of the original and our deconvolution method in two datasets. (A) Distribution of cell type fractions of ground truth in the Wu dataset. **(B)** Distribution of cell type fractions obtained through deconvolution of the Wu dataset using our method. **(C)** Distribution of cell type fractions of ground truth in GSE136831. **(D)** Distribution of cell type fractions obtained through deconvolution of the GSE136831 based on our method.

Compare with other deconvolution methods

We compare our deconvolution method with reference-based methods (Bisque, CIBERSORT, quanTIseq), marker-based methods (xCell), and computer-based methods (EPIC). The deconvolution results of these methods in the Wu dataset and GSE136831 are shown in Fig. S2, Fig. S3, and Fig. S4.

Compared with these methods, our method obtains the best results. As shown in Fig. S2, Fig. S3, and Fig. S4, relative to other methods, the distribution of the results of our deconvolution method is most similar to the ground truth. In the GSE136831 dataset, although our results perform poorly in some cell types, our method is still optimal in terms of overall distribution compared to other methods.

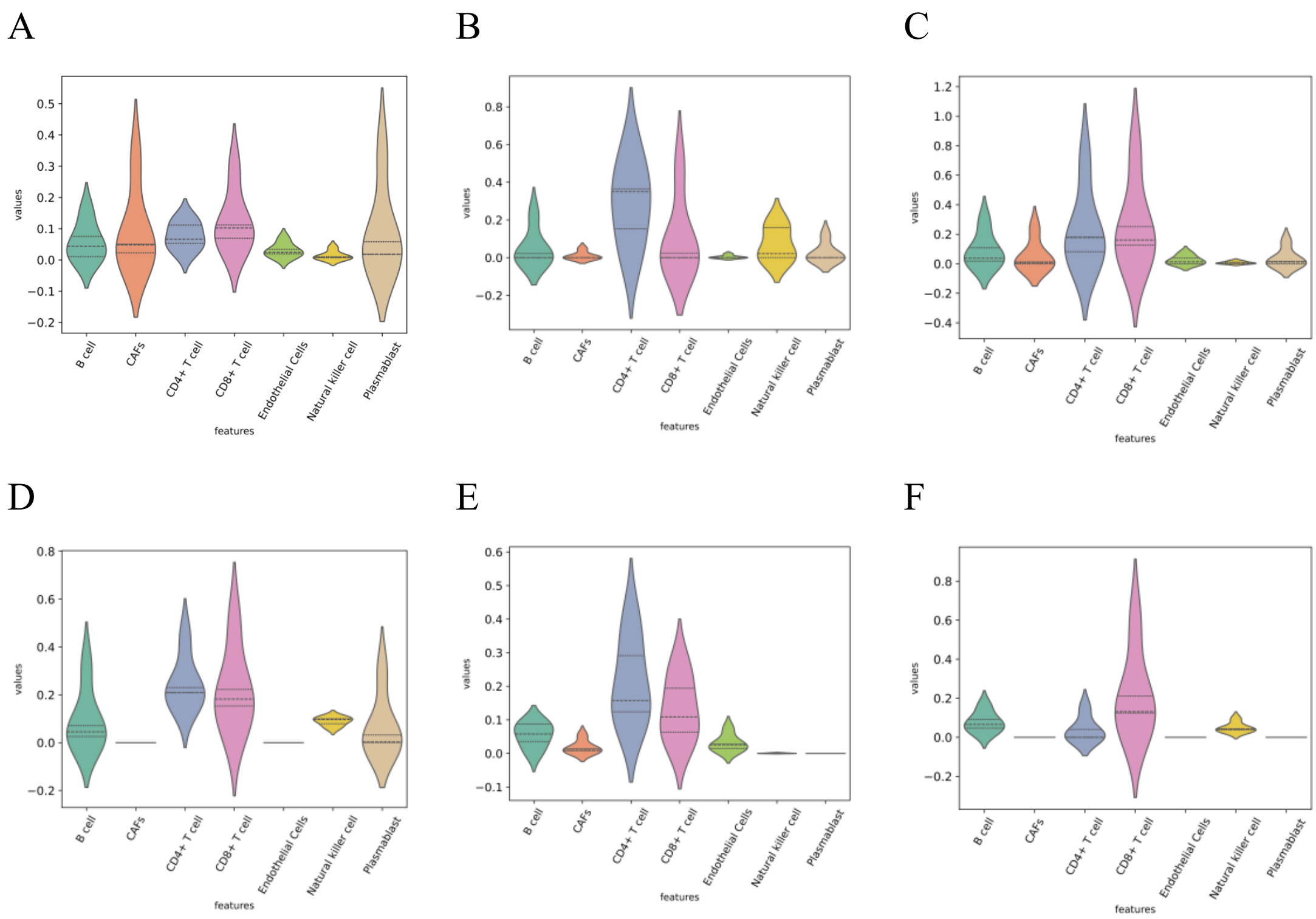

Fig. S3. The distribution of the seven cell type proportions of the compared methods in the Wu dataset. (A) Ground truth of the Wu dataset. **(B)** Bisque. **(C)** xCell. **(D)** CIBERSORT. **(E)** EPIC. **(F)** quantiseq.

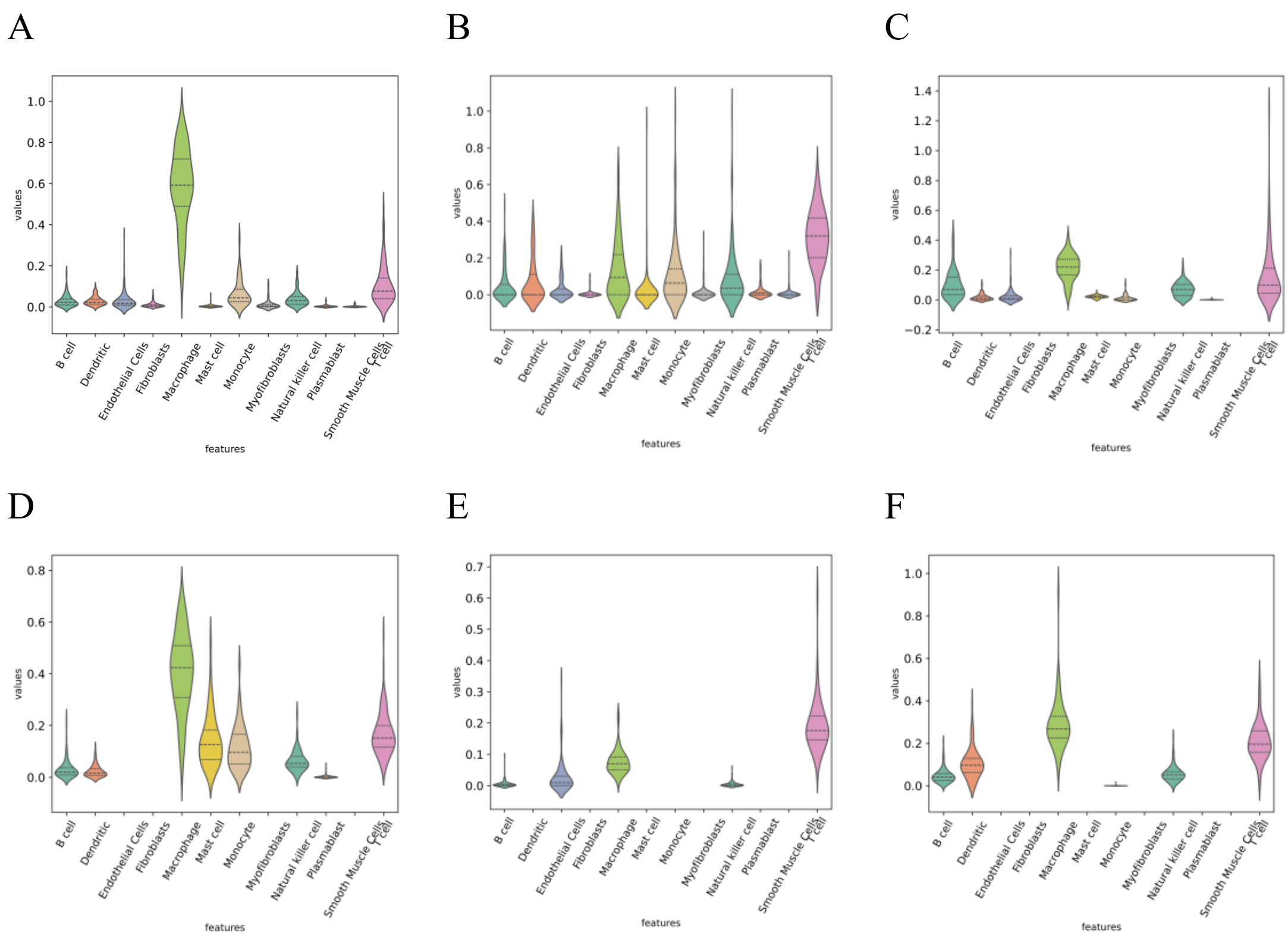

Fig. S4. The distribution of the twelve cell type proportions of compared methods in GSE136831. **(A)** Ground truth of GSE136831. **(B)** Bisque. **(C)** xCell. **(D)** CIBERSORT. **(E)** EPIC. **(F)** quantiseq.

Deconvoluted cell proportions reveal differences in the infiltrability of cancer subtypes

Except for the t-test, we also compared all of the eight cell types based on the Wilcoxon test, and four of eight were significant differences in the TNBC and non-TNBC groups, that is, CAF, CD4+ T cell, endothelial cell, and macrophage, as shown in Fig. S5. Breast cancer indicates the purity of breast cancer cells.

As shown in Fig. S5, the "***" indicates the p-value less than or equal to 0.001, the "**" indicates the p-value less than or equal to 0.01, "*" indicates the p-value less than 0.05, and "ns" indicates the results are not significant, with a p-value greater than 0.05. The reason fibroblasts are significant in the T-test and not significant in the Wilcox test is because the T-test is more sensitive to outliers relative to the Wilcox test, and the significance of fibroblasts is a result of some of the outliers.

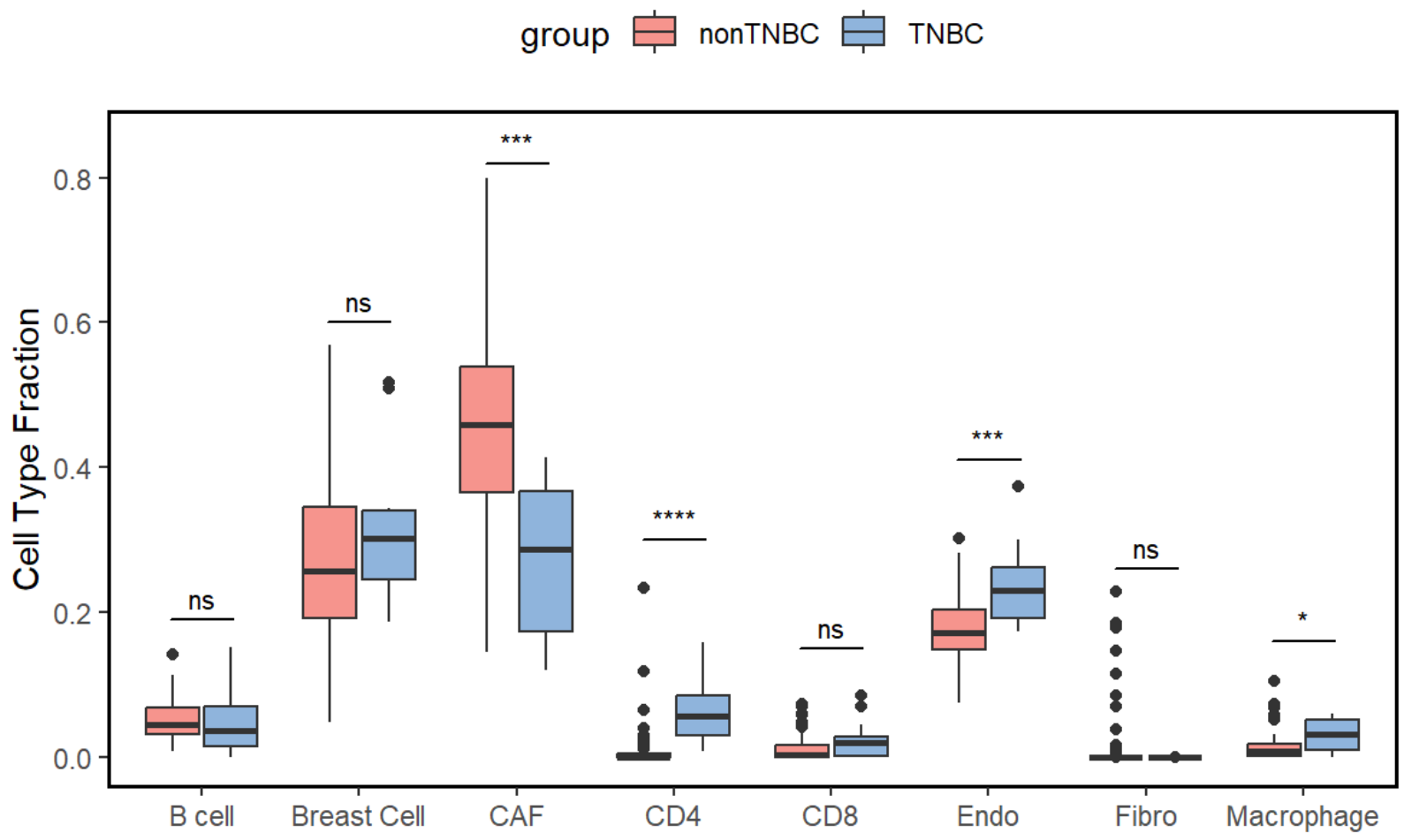

Fig. S5. The Wilcox test results of eight cell types in TNBC and non-TNBC groups.

Based on deconvolution method, we obtained the cell type fraction and expression for each sample in the radiogenomics validation cohort. Fig. S6 shows the

distribution of t-test and Wilcoxon test results of cell type fraction in TNBC and non-TNBC groups of the radiogenomics validation cohort; Fig. S6(A) is the t-test result, and Fig. S6(B) is the Wilcoxon test result. As shown in Fig. S6, in the radiogenomics validation cohort, the CAF and endothelial cells are significantly different in the TNBC and non-TNBC groups based on the t-test, and the CAF and CD4+ T cells are significantly different based on the Wilcoxon test. It can be observed in Fig. S6 that CAF has a higher distribution in the non-TNBC group, while CD4, endothelial, and macrophage have a higher distribution in the TNBC group, and the results are consistent with the radiogenomics discovery cohort. Due to the low data volume, no significant differences were demonstrated in some cell types.

A B

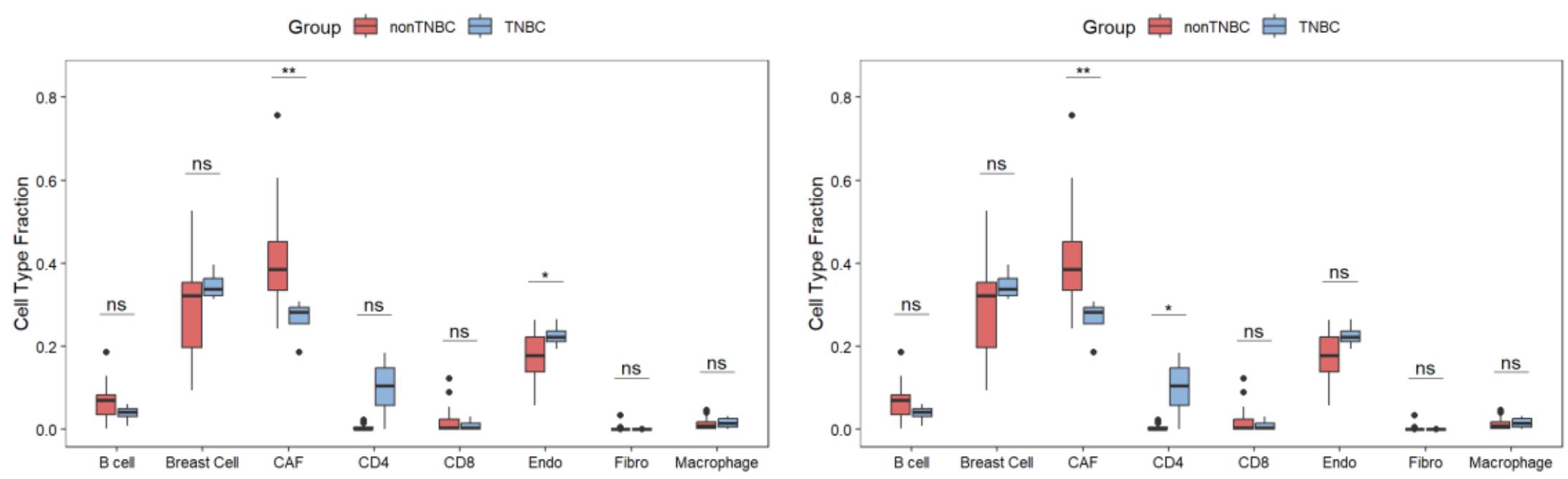

Fig. S6. Distribution and t-test, Wilcoxon test results of cell type fraction in the TNBC and non-TNBC groups of the radiogenomics validation cohort. **(A)** The distribution and t-test results of cell type fractions in the TNBC and non-TNBC groups. **(B)** The distribution and Wilcoxon test results of cell type fractions in the TNBC and non-TNBC groups.

DEG and GSEA reveal the biological function of deconvoluted breast cancer cell expression in TNBC and non-TNBC GSEA

In this study, we employed the deconvolution method to obtain breast cell-specific expression from breast cancer bulk gene expression data. Comparison of gene expression before and after deconvolution by GSEA and DEG showed that despite deconvolution, breast cell gene expression retained its key biological functions. It indicates that the deconvolution method is effective and does not affect the interpretation of biological significance.

Table 2 shows the top 10 DEG results and related description of the breast cancer gene expression after deconvolution. Table 3 shows the top 10 DEG results

and related description of the breast cancer gene expression before deconvolution. The logFC is the log (FoldChange). FoldChange is obtained by subtracting the average expression of the two groups entered. The p-value is calculated by the t-test, and Adj P Value, which means adjusted p-value, was obtained by correcting for the significance of the difference p-value (p-value). The description and reference in Table 2 and Table 3 show the related research of DEG in TNBC or breast cancer. The blanks in the table indicate that there are no relevant studies that were found. The DEG was performed by the *limma* package in R.

Table S2. The top 10 DEG results and related description of breast cancer gene expression after deconvolution.

Gene	logFC	P-Value	Adj P Value	Description	Reference
PSAT1	3367.396	1.62E-25	2.10E-21	Phosphoserine aminotransferase 1 (PSAT1) is a serine biosynthesis-related member of the aminotransferase family and is closely associated with worse prognosis in triple-negative breast cancer (TNBC).	1
FAM171A1	361.7493	2.10E-23	1.36E-19	Sanawar et al. identified FAM171A1, member (A1) of the family with sequence similarity 171, as an overexpressed candidate gene in TNBC cells and tumors as compared to estrogen receptor-	2

				alpha (ERα) positive breast cancer.	
KCNQ4	249.9031	3.04E-21	1.31E-17	Zhang et al. found that lncRNA BC069792 upregulates the protein expression of Potassium Voltage-Gated Channel Q4 (KCNQ4), inhibits the activities of JAK2 and p-AKT, and plays a role in inhibiting breast cancer growth.	3
ESYT3	134.1341	1.03E-20	3.33E-17	-	-
MTHFD1L	893.7359	4.19E-20	1.09E-16	Liu et al. revealed that 5'-tRFCys enhances the expression of downstream metastasis-promoting genes, Mthfd1l and Pafah1b1, by promoting the formation of RNA-binding protein Nucleolin oligomers, thereby enhancing the ability of breast cancer cells to resist programmed death during tumor metastasis.	4
CCNE1	676.8637	1.29E-16	2.40E-13	Zhao et al. proposed that CCNE1 amplification may confer resistance to	5

				chemotherapy and is associated with poor overall survival in TNBC.	
RGMA	14.58018	2.30E-16	3.73E-13	Bao et al. identified 4 hub genes (FOXC1, BCL11A, FAM171A1, and RGMA) that showed a highly positive correlation with the TNBC subtype.	6
PRTFDC1	90.6754	2.04E-19	4.41E-16	-	-
CDCA2	1212.211	3.29E-16	4.20E-13	Luo et al. showed that CDCA2 expression got increased in breast cancer tissues, which greatly enhanced the cell proliferation, migration, and invasion ability.	7
L3MBTL4	31.48405	2.81E-16	4.04E-13	Addou-Klouche et al. identified *L3MBTL4* as a potential tumor suppressor gene of chromosome arm 18p. This gene is targeted by deletion, breakage, and mutations and its mRNA is downregulated in breast tumors.	8

Table S3. The Top10 DEG results and related description of breast cancer gene expression before deconvolution.

Gene	logFC	P.Value	adj.P.Value	Description	Reference
PSAT1	3922.578947	5.34E-25	6.92E-21	Phosphoserine aminotransferase 1 (PSAT1) is a serine biosynthesis-related member of the aminotransferase family and is closely associated with worse prognosis in triple-negative breast cancer (TNBC).	1
FAM171A1	3683.074561	9.05E-19	3.91E-15	Sanawar et al. identified FAM171A1, member (A1) of the family with sequence similarity 171, as an overexpressed candidate gene in TNBC cells and tumors as compared to estrogen receptor-alpha (ERα) positive breast cancer.	2

KCNQ4	568.1315789	1.12E-19	7.28E-16	Zhang et al. found that lncRNA BC069792 upregulates the protein expression of Potassium Voltage-Gated Channel Q4 (KCNQ4), inhibits the activities of JAK2 and p-AKT, and plays a role in inhibiting breast cancer growth.	3
ESYT3	281.1798246	3.52E-17	1.14E-13	-	-
MTHFD1L	1612.916667	9.06E-17	1.68E-13	Liu et al. revealed that 5'-tRFCys enhances the expression of downstream metastasis-promoting genes, Mthfd1l and Pafah1b1, by promoting the formation of RNA-binding protein Nucleolin oligomers, thereby	4

				enhancing the ability of breast cancer cells to resist programmed death during tumor metastasis.	
CCNE1	778.0482456	8.39E-17	1.68E-13	Zhao et al. proposed that CCNE1 amplification may confer resistance to chemotherapy and is associated with poor overall survival in TNBC.	5
HMGA1	14023.27193	4.68E-16	6.74E-13	High Mobility Group A1 (HMGA1) is an architectural chromatin factor involved in the regulation of gene expression and a master regulator in Triple Negative Breast Cancer (TNBC).	9
CDCA2	1270.355263	4.08E-16	6.61E-13	Luo et al. showed that CDCA2 expression got	7

				increased in breast cancer tissues, which greatly enhanced the cell proliferation, migration, and invasion ability.	
BCL11A	2332.495614	8.95E-17	1.68E-13	Bao et al. identified 4 hub genes (FOXC1, BCL11A, FAM171A1, and RGMA) that showed a highly positive correlation with TNBC subtype.	6
TTLL4	3567.701754	9.80E-16	1.27E-12	Julia Arnold et al. show that Tubulin Tyrosine Ligase Like 4 (TTLL4) overexpression in breast cancer is associated with cerebral metastasis development.	10

Deep Radiogenomics Model correlate MRI with microenvironment and cancer subtypes

In our deep radiogenomics model, we combined gene data with images data to obtain the image-subtype and image-microenvironment relationship, the whole module structure as shown in Fig. S7. The Fig. S7(A) shows the overview of the model structure and Fig. S7(B) shows the UNet module structure. Fig. S7(C) shows the SMLP module structure, the details of these modules as described in the article.

A

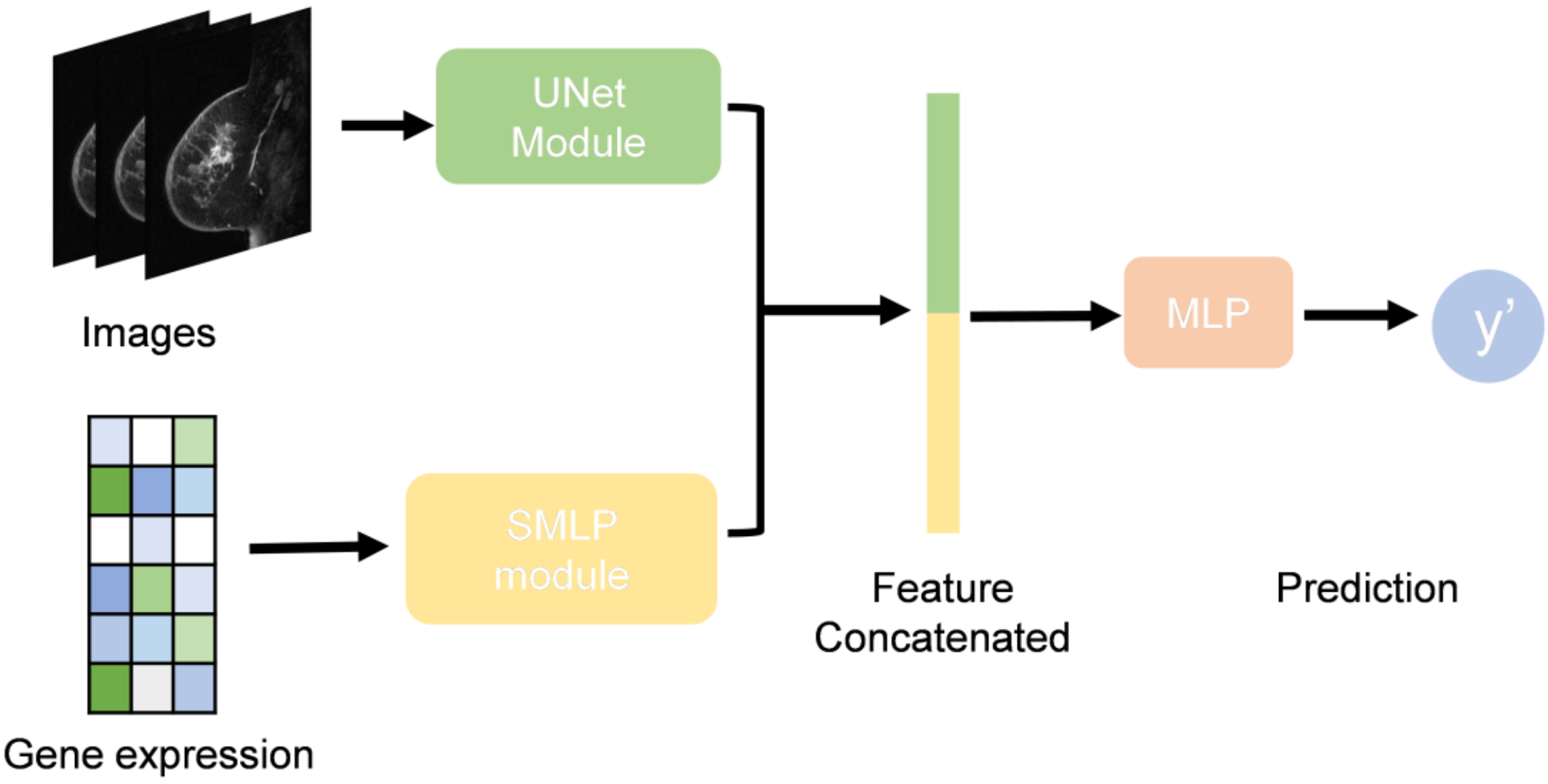

B

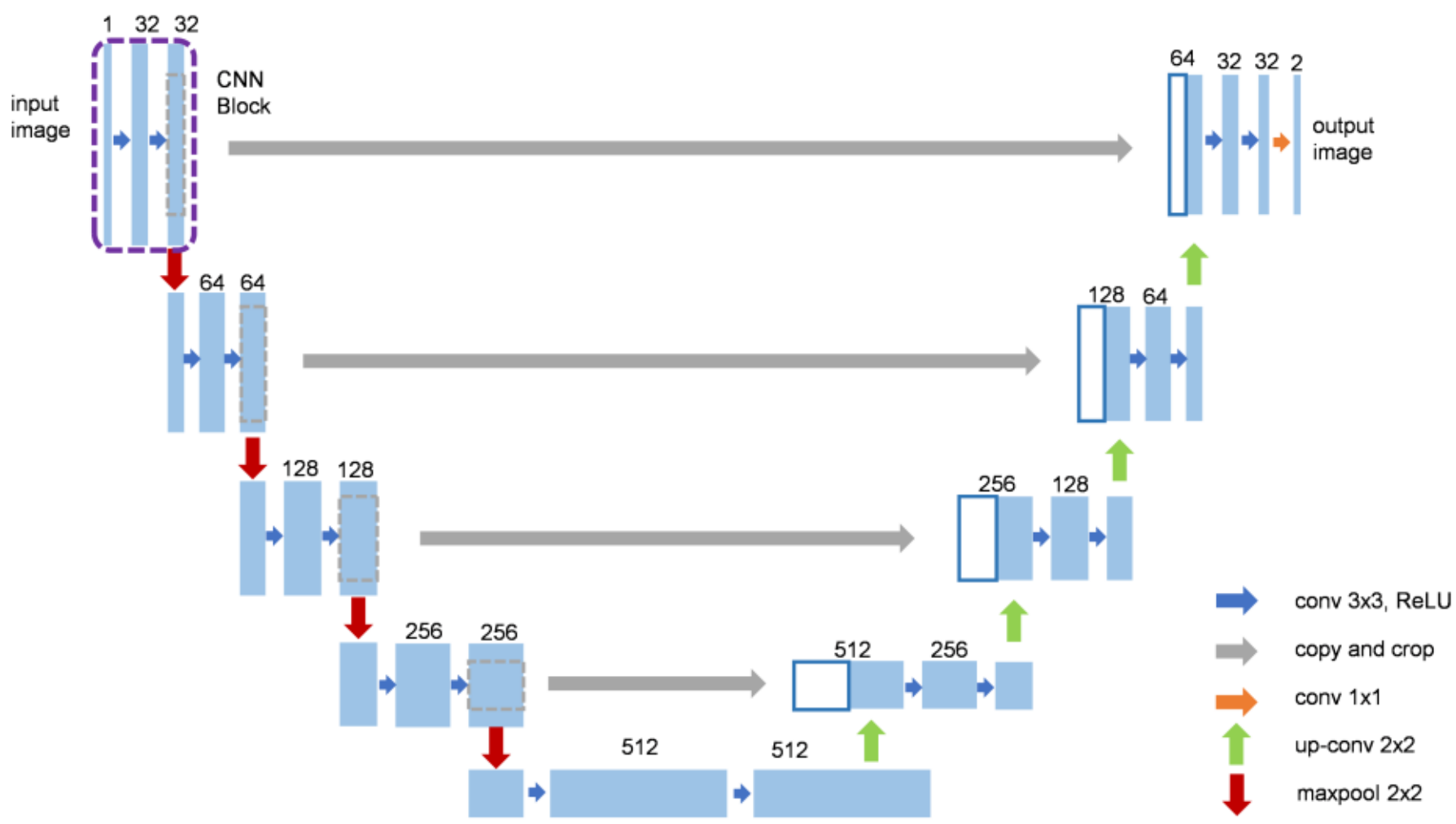

C

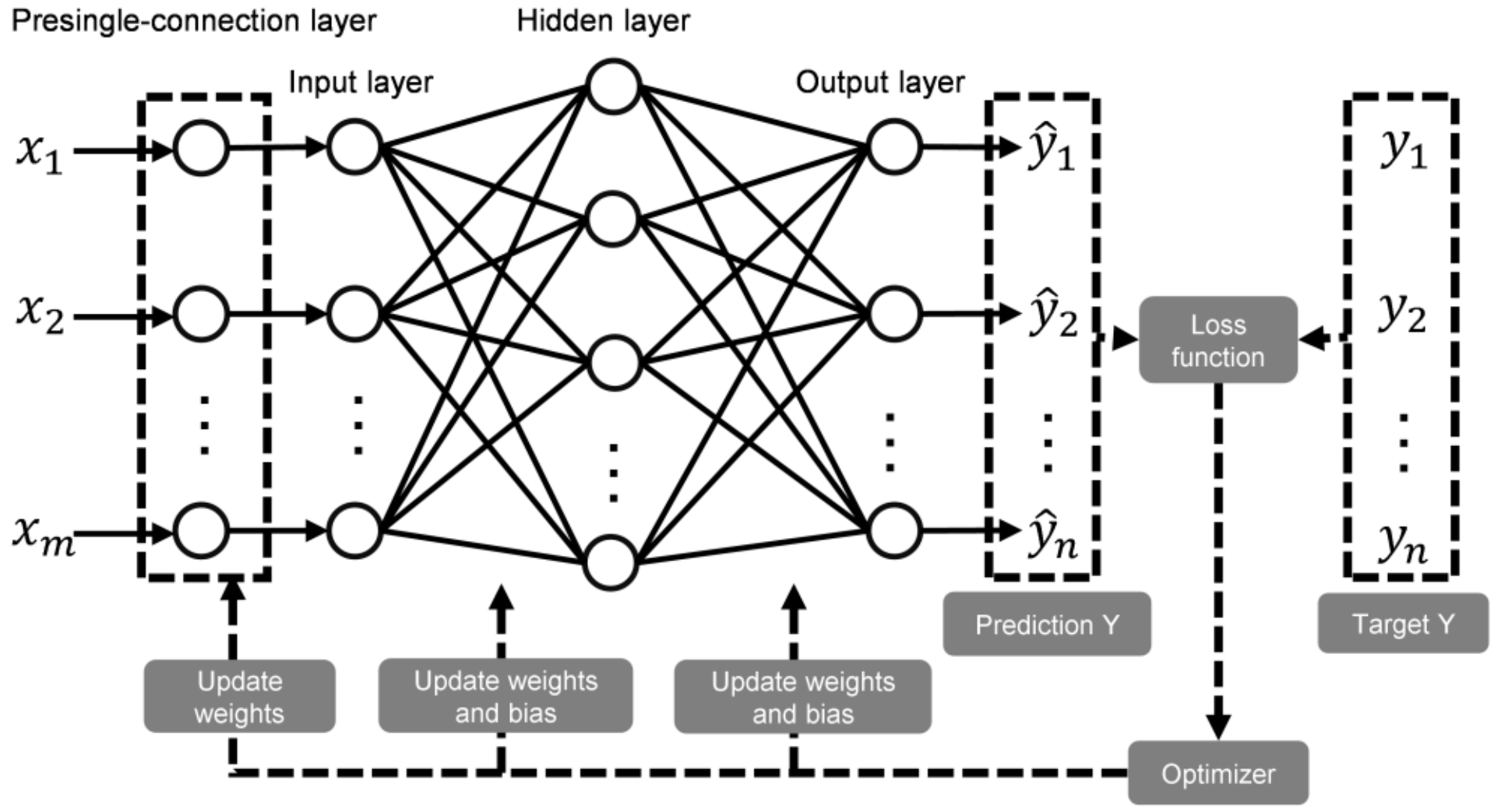

Fig. S7. Overview of the model structure. (A) The whole structure of the model, including the UNet module for image feature extraction and the SMLP module for gene feature extraction, combined the gene features and image features and input them into the MLP module for classification prediction. **(B)** Structure of UNet module. **(C)** Structure of SMLP module.

To validate the effective of the combine data, we construct the models that only utilized images data. The Fig. S8 shows the attention area that with and without gene data from image-microenvironment and image-subtype model of patient TCGA-AO-A03V. Fig. S8(A) shows the attention area from image-microenvironment and image-subtype model that without genomic data, while Fig. S8(B) shows the attention area that with genomic data.

As shown in Fig. S8, the area of attention is not obvious in the model without genomic data relative to the model with genomic data. The major reason is that the data dimension can be expanded by adding genomic data, which improves the model effect while avoiding model overfitting.

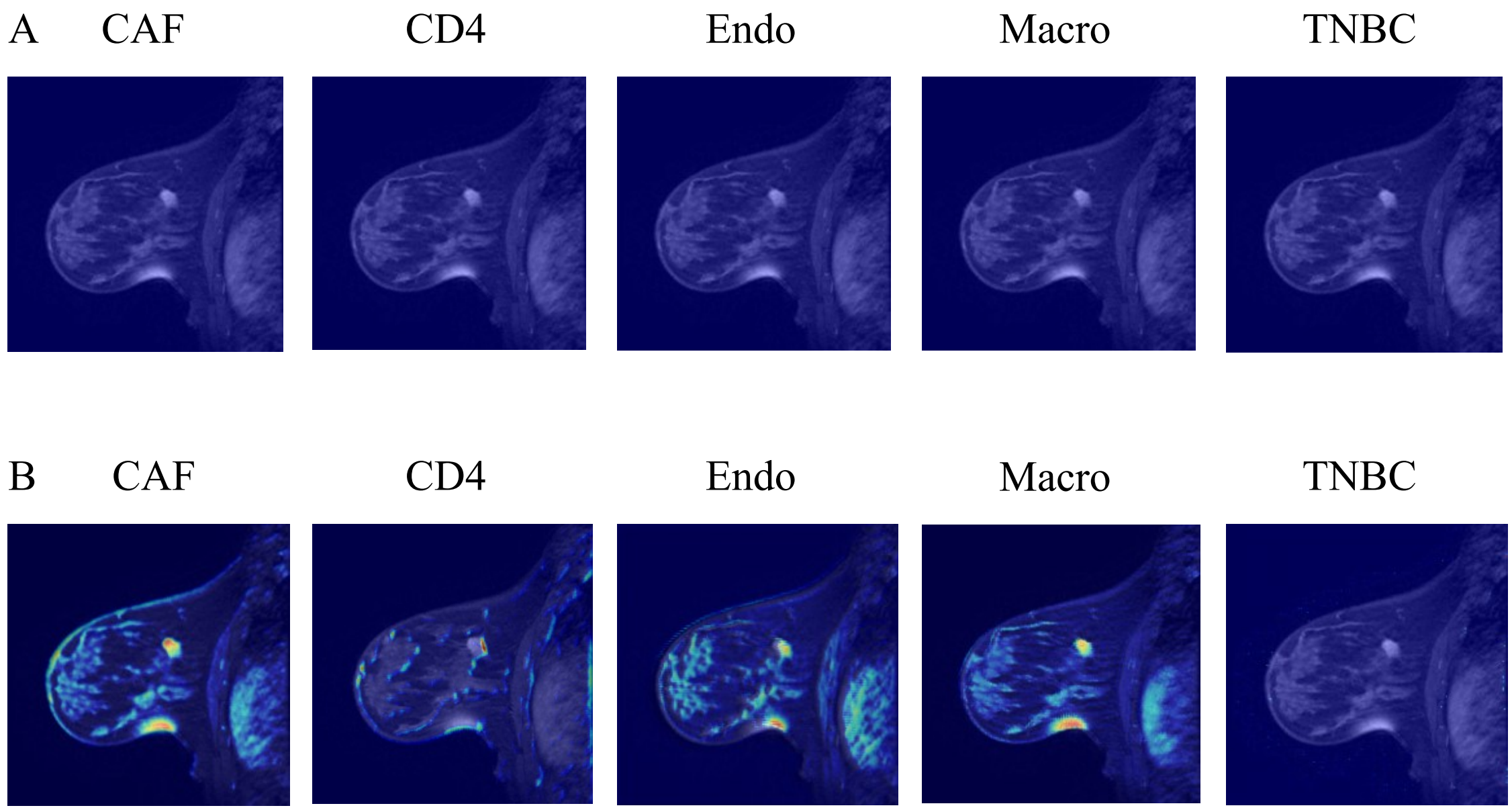

Fig. S8. The attention region of image-microenvironment model and image-subtype model with and without gene data. (A) From left to right, there are the attention region of image-CAF, image-CD4, image-Endo, image-Macro, image-TNBC model that trained with image data while without gene data. **(B)** From left to right, there are the attention region of image-CAF, image-CD4, image-Endo, image-Macro, image-TNBC model that was trained with image data and gene data.

Based on the image data of radiogenomic discovery cohort, five models were trained and tested with images as input features (image-CAF, image-CD4, image-Endo, image-Macro, image-TNBC), the microenvironmental HIGH and LOW and cancer subtypes as classification labels, respectively. The image feature extracted by U-Net module (Fig. S7 (B)) and three layers of MLP as a classification module, all the results of five image-only models are shown in Table S4. The accuracy of the image-TNBC model on the test set is 0.71 with an AUC of 0.50. The PR-AUC and Sensitivity and Specificity in the image-TNBC model are 0.60, 0.20, and 0.80. The accuracy of the image-TNBC, image-CAF, image-CD4 and image-Endo, image-Macro models without the genome was 0.71, 0.50 and 0.50, 0.49, and 0.52, respectively, while the accuracy of the image-TNBC, image-CAF, image-CD4 and image-CD8 models with the genome were all 1. The results show that the addition of genomic features solves the model overfitting problem and improves the model effect.

Table S4. The results of image-microenvironment and image-subtype model (image-only) in test set.

	**Accuracy**	**ROC-AUC**	**PR-AUC**	**Sensitivity**	**Specificity**
**Image-TNBC**	0.71	0.50	0.60	0.20	0.80
**Image-CAF**	0.50	0.50	0.60	0.20	0.80
**Image-CD4**	0.50	0.50	0.50	0.0	1.0
**Image-Endo**	0.49	0.50	1.0	1.0	0.0
**Image-Macro**	0.52	0.50	0.50	0.0	1.0

Radiomic features reveal the effectiveness of CAF-I-marker

Based on image-CAF model, we can obtain the CAF-I-marker, and create the CAF annotation to extract CAF-related radiomic features. For the radiomic feature, we extract 10 first-order features, 10 shape-2D features and 16 GLCM features. The descriptions of all these radiomic features are as shown in Table S5.

Table S5. The description of the 36 radiomic features.

Feature name	Feature type	Description
Entropy	firstorder	Entropy specifies the uncertainty/randomness in the image values. It measures the average amount of information required to encode the image values.
InterquartileRange	firstorder	Interquartile range = P75−P25; here P25 and P75 are the 25th and 75th percentiles of the gray level intensity within the ROI, respectively.
Kurtosis	firstorder	Kurtosis is a measure of the 'peakedness' of the distribution of values in the image ROI. A higher kurtosis

		implies that the mass of the distribution is concentrated towards the tail(s) rather than towards the mean. A lower kurtosis implies the reverse: that the mass of the distribution is concentrated towards a spike near the mean value.
Maximum	firstorder	The maximum gray level intensity within the ROI.
Mean	firstorder	The average gray level intensity within the ROI.
Median	firstorder	The median gray level intensity within the ROI.
Minimum	firstorder	The minimum gray level intensity within the ROI.
Range	firstorder	The range of gray values in the ROI.
Skewness	firstorder	Skewness measures the asymmetry of the distribution of values about the Mean value. Depending on where the tail is elongated and the mass of the distribution is concentrated, this value can be positive or negative.
StandardDeviation	firstorder	Standard Deviation measures the amount of variation or dispersion from the Mean Value.
Elongation	shape2D	Elongation shows the relationship between the two

		largest principal components (λmajor and λminor) in the ROI shape. For computational reasons, this feature is defined as the inverse of true elongation.
MajorAxisLength	shape2D	This feature yields the largest axis length of the ROI-enclosing ellipsoid and is calculated using the largest principal component λmajor.
MaximumDiameter	shape2D	Maximum diameter is defined as the largest pairwise Euclidean distance between tumor surface mesh vertices.
MeshSurface	shape2D	To calculate the surface area, first calculate the signed surface area of each triangle in the mesh. The total surface area is then obtained by taking the sum of all calculated sub-areas.
MinorAxisLength	shape2D	This feature yields the second-largest axis length of the ROI-enclosing ellipsoid and is calculated using the largest principal component λminor.
Perimeter	shape2D	To calculate the perimeter, first the perimeter of each line in the mesh circumference is calculated. The total perimeter is then obtained by taking the

		sum of all calculated sub-areas.
PerimeterSurfaceRatio	shape2D	PerimeterSurfaceRatio means Perimeter to Surface ratio, a lower value indicates a more compact (circle-like) shape. This feature is not dimensionless, and is therefore (partly) dependent on the surface area of the ROI.
PixelSurface	shape2D	The surface area of the ROI is approximated by multiplying the number of pixels in the ROI by the surface area of a single pixel. This is a less precise approximation of the surface area.
SphericalDisproportion	shape2D	Spherical Disproportion is the ratio of the perimeter of the tumor region to the perimeter of a circle with the same surface area as the tumor region, and by definition, the inverse of Sphericity.
Sphericity	shape2D	Sphericity is the ratio of the perimeter of the tumor region to the perimeter of a circle with the same surface area as the tumor region and therefore a measure of the roundness of the shape of the tumor region relative to a circle.

Autocorrelation	GLCM	Autocorrelation is a measure of the magnitude of the fineness and coarseness of texture.
ClusterProminence	GLCM	Cluster Prominence is a measure of the skewness and asymmetry of the GLCM. A higher value implies more asymmetry about the mean while a lower value indicates a peak near the mean value and less variation about the mean.
ClusterShade	GLCM	Cluster Shade is a measure of the skewness and uniformity of the GLCM. A higher cluster shade implies greater asymmetry about the mean.
Contrast	GLCM	Contrast is a measure of the local intensity variation, favoring values away from the diagonal. A larger value correlates with a greater disparity in intensity values among neighboring voxels.
Correlation	GLCM	Correlation is a value between 0 (uncorrelated) and 1 (perfectly correlated) showing the linear dependency of gray level values to their respective voxels in the GLCM.
DifferenceAverage	GLCM	Difference Average measures the relationship between

		occurrences of pairs with similar intensity values and occurrences of pairs with differing intensity values.
DifferenceEntropy	GLCM	Difference Entropy is a measure of the randomness/variability in neighborhood intensity value differences.
DifferenceVariance	GLCM	Difference Variance is a measure of heterogeneity that places higher weights on differing intensity level pairs that deviate more from the mean.
Imc1	GLCM	IMC1 assesses the correlation between the probability distributions of i and j (quantifying the complexity of the texture), using mutual information.
Imc2	GLCM	IMC2 also assesses the correlation between the probability distributions of i and j (quantifying the complexity of the texture).
InverseVariance	GLCM	Inversevariance=$\sum_{k=1}^{N_g-1}\frac{p_{x-y}(k)}{k^2}$. Note that k=0 is skipped, as this would result in a division by 0.
JointEnergy	GLCM	Energy is a measure of homogeneous patterns in the image. A greater Energy

		implies that there are more instances of intensity value pairs in the image that neighbor each other at higher frequencies.
JointEntropy	GLCM	Joint entropy is a measure of the randomness/variability in neighborhood intensity values.
MaximumProbability	GLCM	Maximum Probability is occurrences of the most predominant pair of neighboring intensity values.
SumAverage	GLCM	Sum Average measures the relationship between occurrences of pairs with lower intensity values and occurrences of pairs with higher intensity values.
SumSquares	GLCM	Sum of Squares or Variance is a measure of the distribution of neighboring intensity level pairs about the mean intensity level in the GLCM.

Based on t-test, we obtained the significant features in all 36 CAF-related radiomic features in TNBC and non-TNBC groups in radiogenomics discovery cohort. In CAF-related radiomic features, there are eight significant features calculated by t-test, the details as shown in Table S6.

Table S6. The eight significant features in CAF-related radiomic features.

**First order feature**	**P-value**	**Shape-2D feature**	**P-value**	**GLCM feature**	**P-value**
StandardDeviation	3.43E-05	MajorAxisLength	0.001403	JointEntropy	0.049934
Kurtosis	0.021549			DifferenceVariance	0.033185
Minimum	0.000909			MaximumProbability	0.023439
InterquartileRange	9.99E-05				

Moreover, we obtained the significant features by t-test in all 36 CAF-related radiomic features in CAF-high and -low groups in radiogenomics discovery cohort. As shown in Table S7, there are 20 significant features in CAF-high and -low groups.

Table S7. The significant features of CAF-related radiomic features in CAF-high and -low groups.

**First order feature**	**P-value**	**Shape-2D feature**	**P-value**	**GLCM feature**	**P-value**
Mean	0.022395	MeshSurface	0.00011	SumSquares	0.007119
StandardDeviation	0.000987	PixelSurface	4.83E-05	SumAverage	0.004454
Entropy	0.00327	Perimeter	3.65E-05	Imc1	0.003659
Minimum	0.002198	Sphericity	6.79E-05	Imc2	0.018632

Maximum	0.002183	SphericalDisproportion	1.93E-05	Autocorrelation	0.003121
Median	0.013399	MaximumDiameter	5.36E-06		
Range	8.15E-05	MajorAxisLength	0.000104		
		MinorAxisLength	0.029713		

In addition to the CAF-marker, we also extracted the Endo-I marker and obtained Endo-related radiomic features. Based on t-test, we analyzed the feature difference in TNBC and non-TNBC groups of Endo-related radiomic features; there are six significant difference features as shown in Table S8.

Table S8. The six significant features in Endo-related radiomic features

First order feature	P-value	Shape-2D feature	P-value
StandardDeviation	9.52E-16	MinorAxisLength	0.012615
Skewness	0.017977	Elongation	0.000508
Maximum	0.035354		
Median	0.008219		

The distributions of CAF-related first-order and shape2D, GLCM features of radiogenomics discovery cohort are shown in Fig. S9. Fig. S9(A) is the distribution of first-order features of CAF-related radiomic features, Fig. S9(B) is shape2D features and Fig. S9(C) is GLCM features. As shown in Fig. S9(A), for the first-order features, the TNBC group has a higher distribution in Mean, Minimum, Median, Kurtosis than the non-TNBC group, and a lower distribution in StandarDeviation, Maximum, InterquartileRange, Range, Skewness and Entropy than the non-TNBC group. As shown in Fig. S9(B), for the shape-2D features, the TNBC group has a higher distribution in Elongation, Sphericity than the non-TNBC group, and a lower distribution in MajorAxisLength, MaximumDiameter, MeshSurface, MinorAxisLength, Perimeter, PerimeterSurfaceRatio, PixelSurface, and SphericalDisproportion than the non-TNBC group. As shown in Fig. S9(C), for the GLCM features, the TNBC group has a higher distribution in Autocorrelation,

ClusterShade, Contrast, DifferenceAverage, DifferenceEntropy, DifferenceVariance, Imc1, InverseVariance, JointEntropy, SumAverage, SumSquares, and a lower distribution in ClusterProminence, Correlation, Imc2, JointEnergy, MaximumProbability than the non-TNBC group.

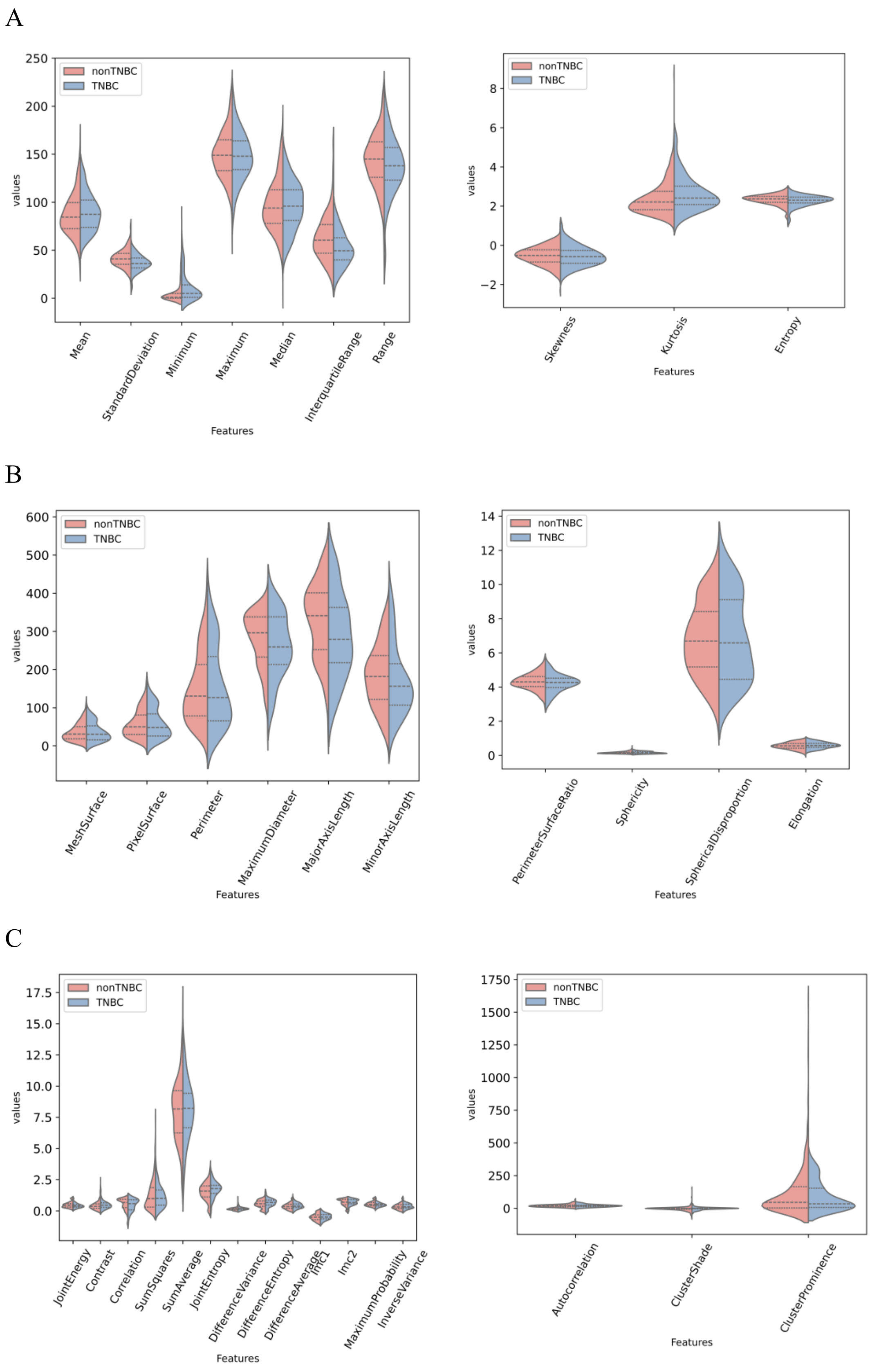

Fig. S9. The distribution of CAF-related radiomic features in TNBC and non-TNBC groups of radiogenomics discovery cohort. (A) The distribution of 10 fristorder features in TNBC and non-TNBC groups. **(B)** The distribution of 10

shape2D features in TNBC and non-TNBC groups. **(C)** The distribution of 16 GLCM features in TNBC and non-TNBC groups.

For Endo-I-marker, we extracted the Endo-related radiomic features, as shown in Fig. S10. The Fig. S10(A) is the distribution of first-order features in TNBC and non-TNBC groups of radiogenomics discovery cohort, and the Fig. S10(B) is the distribution of shape2D features, the Fig. S10(C) is the distribution of GLCM features.

As shown in Fig. S10(A), for the first-order features, the TNBC group has a higher distribution in StandarDeviation, Mean, Minimum, Median, Maximum, Skewness, Entropy than the non-TNBC group, and a lower distribution in Kurtosis, InterquartileRange, Range than the non-TNBC group. As shown in Fig. S10(B), for the shape-2D features, the TNBC group has a higher distribution in MeshSurface, Elongation, Sphericity than the non-TNBC group, and a lower distribution in MajorAxisLength, MaximumDiameter, MinorAxisLength, Perimeter, PerimeterSurfaceRatio, PixelSurface, and SphericalDisproportion than the non-TNBC group. As shown in Fig. S10(C), for the GLCM features, the TNBC group has a higher distribution in Autocorrelation, ClusterShade, Contrast, DifferenceAverage, DifferenceEntropy, DifferenceVariance, Imc2, InverseVariance, JointEntropy, SumAverage, SumSquares, and a lower distribution in ClusterProminence, Correlation, Imc1, JointEnergy, MaximumProbability than the non-TNBC group. Comparing the CAF-related radiomic features with the Endo-related radiomic features, they have the same distribution for 28 out of all 36 features, indicating that the TNBC group has some significant properties in microenvironment-related radiomic features.

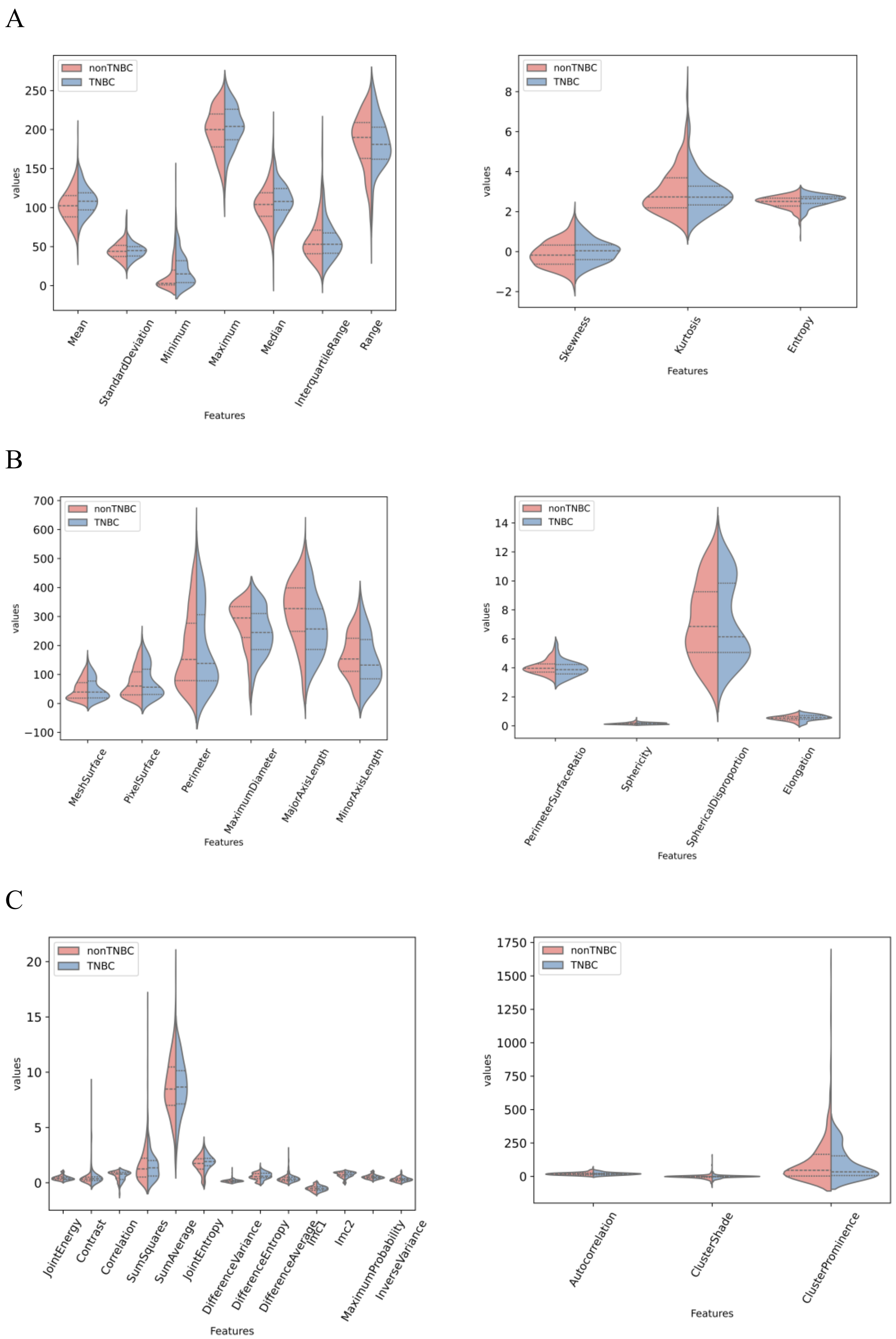

Fig. S10. The distribution of Endo-related radiomic features in TNBC and non-TNBC groups of radiogenomics discovery cohort. **(A)** The distribution of 10 fristorder features in TNBC and non-TNBC groups. **(B)** The distribution of 10

shape2D features in TNBC and non-TNBC groups. **(C)** The distribution of 16 GLCM features in TNBC and non-TNBC groups.

Although Endo-I-marker obtained clearer attention areas in the tumor environment, it did not perform well on the radiogenomics validation cohort, as shown in Fig. 4 in the article, so we used the better-performing CAF-I-marker for the subsequent analyses. For the image-only dataset, we obtained the CAF-I-marker based on CAF-low group, since it has been proved that CAF-low group has a significant relationship with the TNBC. Based on CAF-I-markers, we extracted the CAF-related radiomic features for radiogenomics validation cohort, the radiomics validation cohort and clinical cohort, as shown in Figs. S11, S12 and S13, and S14. Fig. S11 shows the distribution of CAF-related radiomic features in TNBC and non-TNBC groups in radiogenomics validation cohort. Fig. S11(A) is the first-order features and Fig. S11(B) is Shape2D features, Fig. S11(C) is the GLCM features. As shown in Fig. S11, the TNBC group has a higher distribution in Kurtosis, Mean, Autocorrelation, Imc1, SumAverage, SumSquares and Sphericity, a lower distribution in Entropy, InterquartileRange, Range, Skewness, StandardDeviation, Imc2, MaximumDiameter, MeshSurface, Perimeter, PerimeterSurfaceRatio than non-TNBC groups, the same as the radiogenomics discovery cohort.

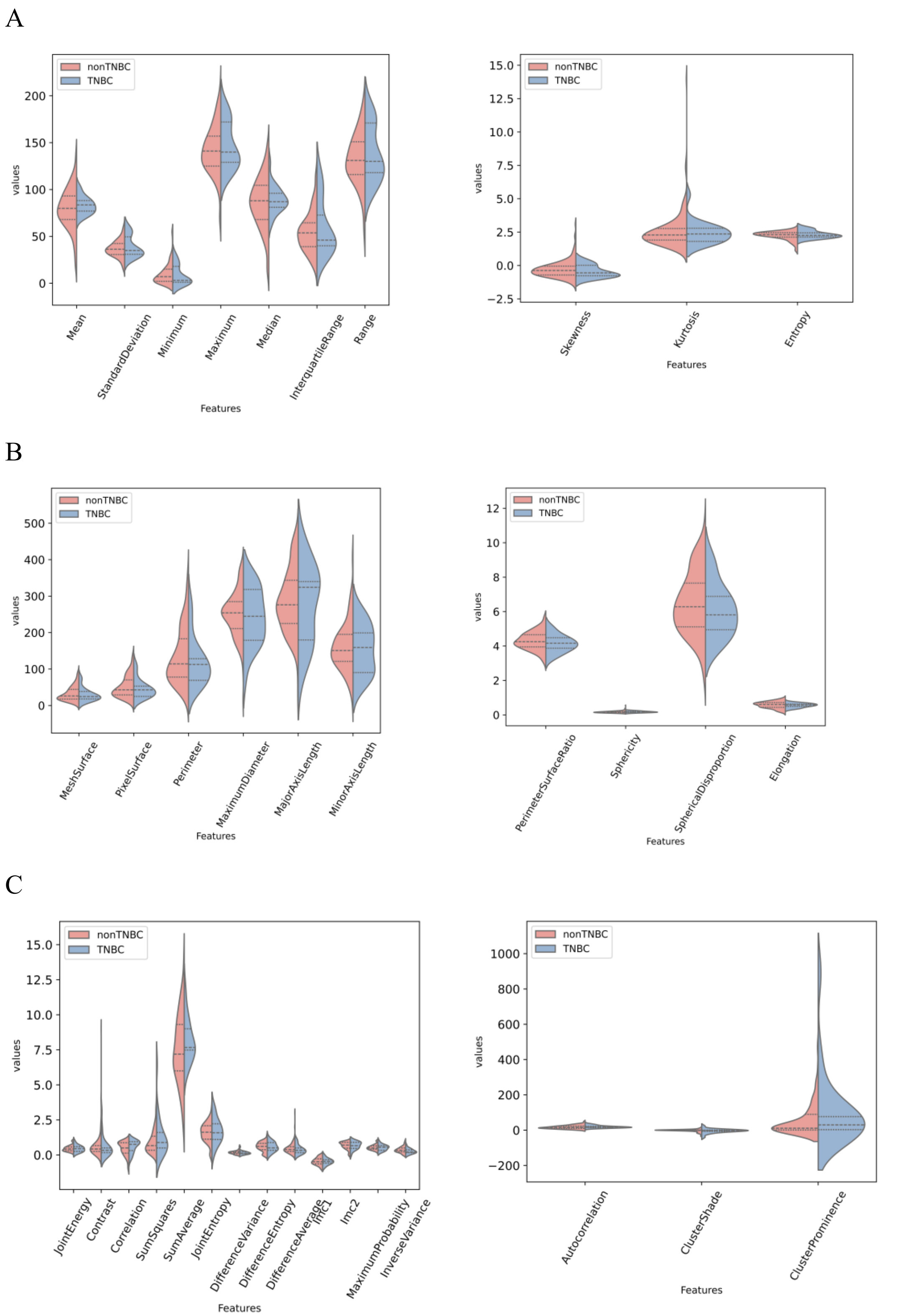

Fig. S11. The distribution of CAF-related radiomic features for TNBC and non-TNBC groups in radiogenomics validation cohort. (A) The distribution of 10 first-order features in TNBC and non-TNBC groups. **(B)** The distribution of 10

shape2D features in TNBC and non-TNBC groups. **(C)** The distribution of 16 GLCM features in TNBC and non-TNBC groups.

Fig. S12 shows the distribution of CAF-related radiomic features in TNBC and non-TNBC groups in radiomics cohort of ISPY. Fig. S12(A) is the first order features and Fig. S12(B) is Shape2D features, Fig. S12(C) is the GLCM features. As shown in Fig. S12, the TNBC group has a higher distribution in Kurtosis, Maximum, Mean, Median, Autocorrelation, ClusterShade, lmc1, Sphericity and SumAverage, a lower distribution in Entropy, InterquartileRange, Skewness, ClusterProminence, Imc2, MajorAxisLength, MaximumDiameter, MeshSurface, MinorAxisLength, Perimeter, PixelSurface than non-TNBC group, the same as the distribution of TNBC radiomic features in radiogenomics discovery cohort.

A

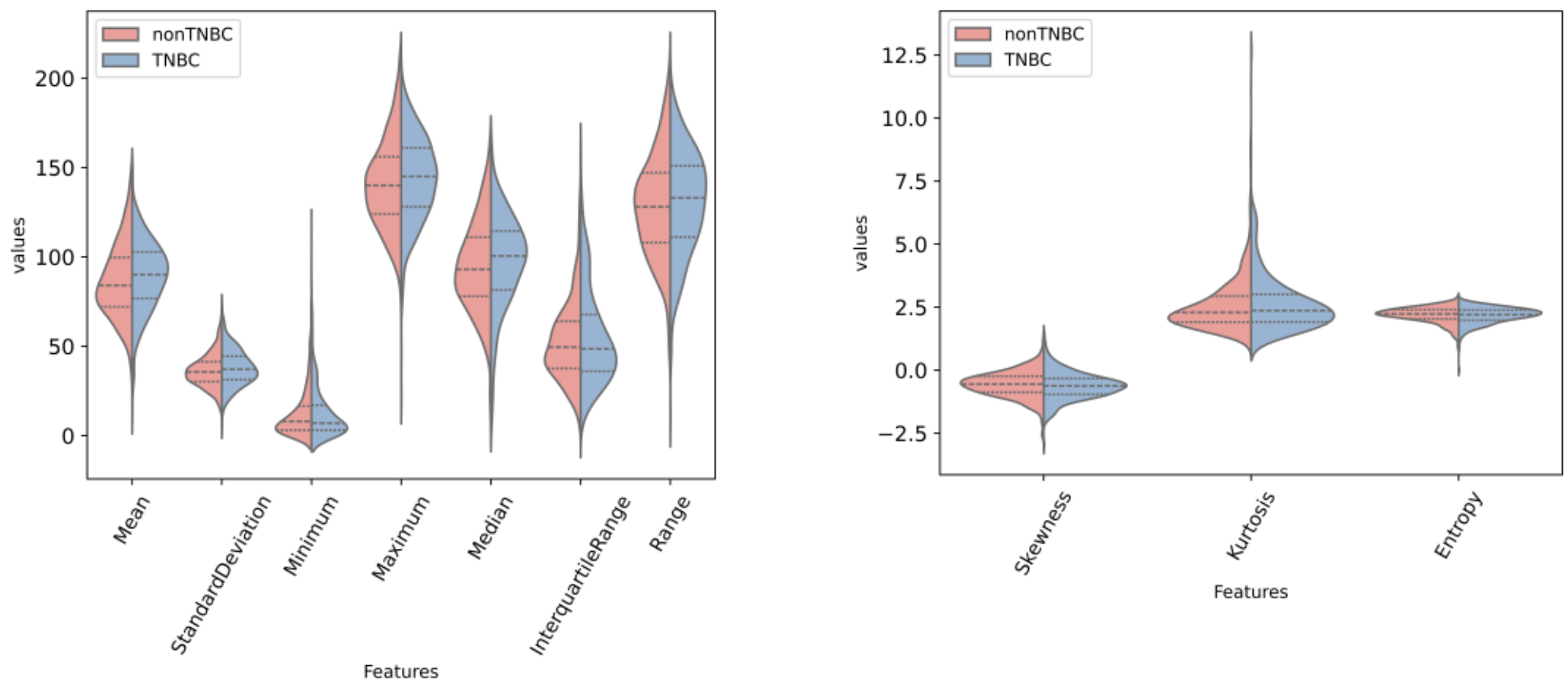

B

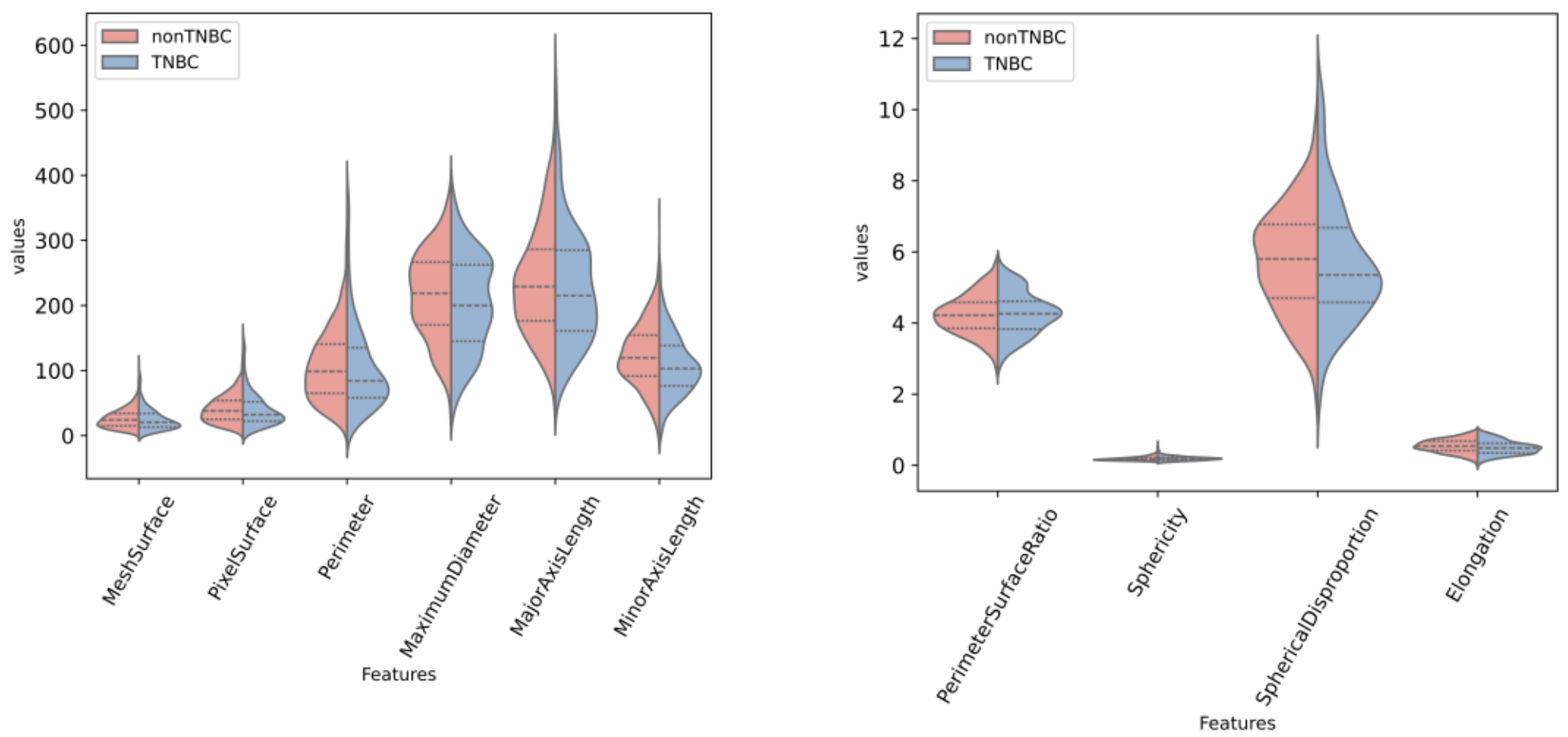

C

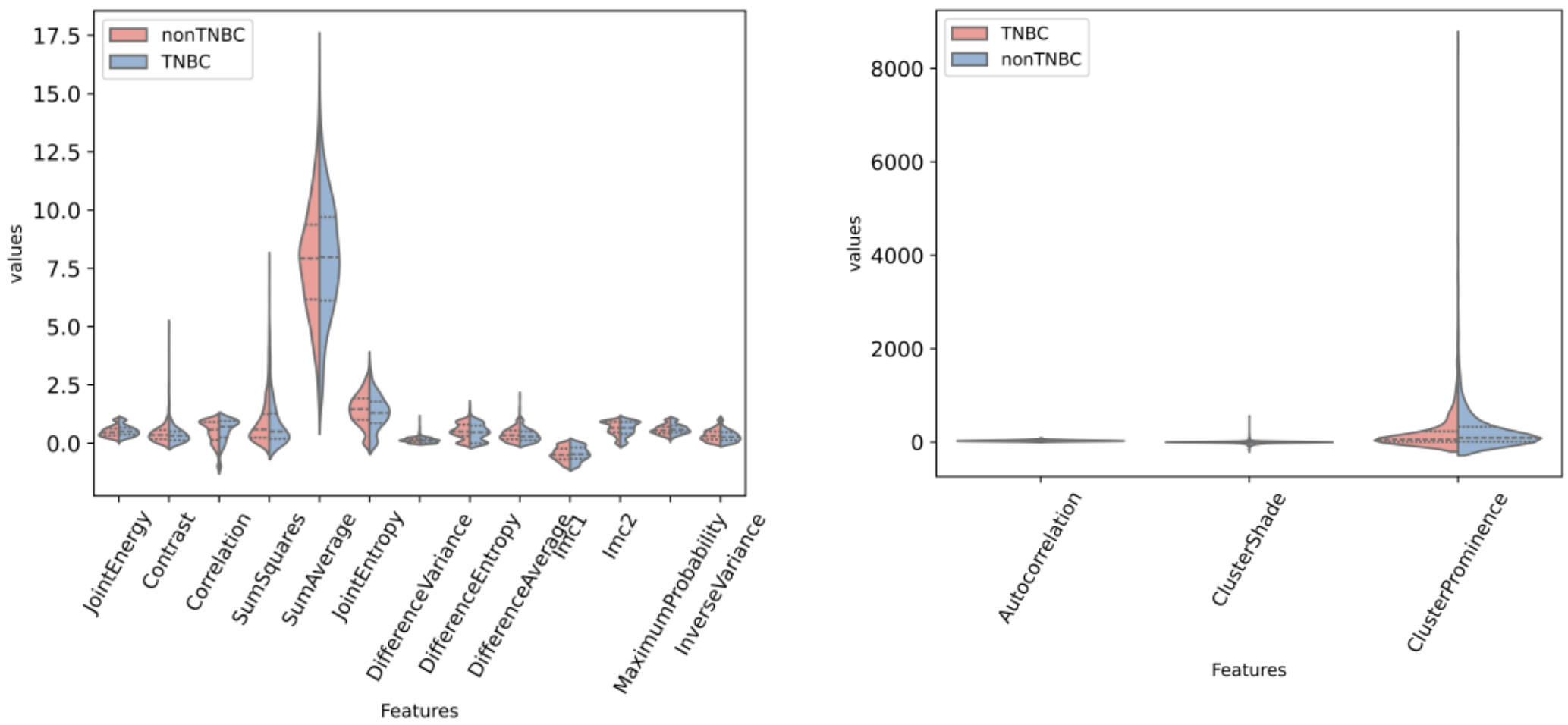

Fig. S12. The distribution of CAF-related radiomic features in radiomics cohort of ISPY. (A) The distribution of 10 fristorder features in TNBC and non-TNBC groups. **(B)** The distribution of 10 shape2D features in TNBC and non-TNBC groups. **(C)** The distribution of 16 GLCM features in TNBC and non-TNBC groups.

Fig. S13 shows the distribution of CAF-related radiomic features in TNBC and non-TNBC groups in radiomics cohort of NACT-Pilot. Fig. S13(A) is the first order features and Fig. S13(B) is Shape2D features, Fig. S13(C) is the GLCM features.

In the same way, based on CAF annotation to extract CAF-related radiomic features, then divided into TNBC and non-TNBC groups. As shown in Fig. S13, the TNBC group has a higher distribution in Kurtosis, Maximum, Mean, Median, Autocorrelation, Contrast, DifferenceAverage, DifferenceEntropy, DifferenceVariance, JointEntropy, SumAverage, SumSquares, Elongation and SphericalDisproportion, a lower distribution in Skewness, Correlation, JointEnergy, MaximumProbability than non-TNBC group, the same as the distribution of TNBC radiomic features in radiogenomics discovery cohort.

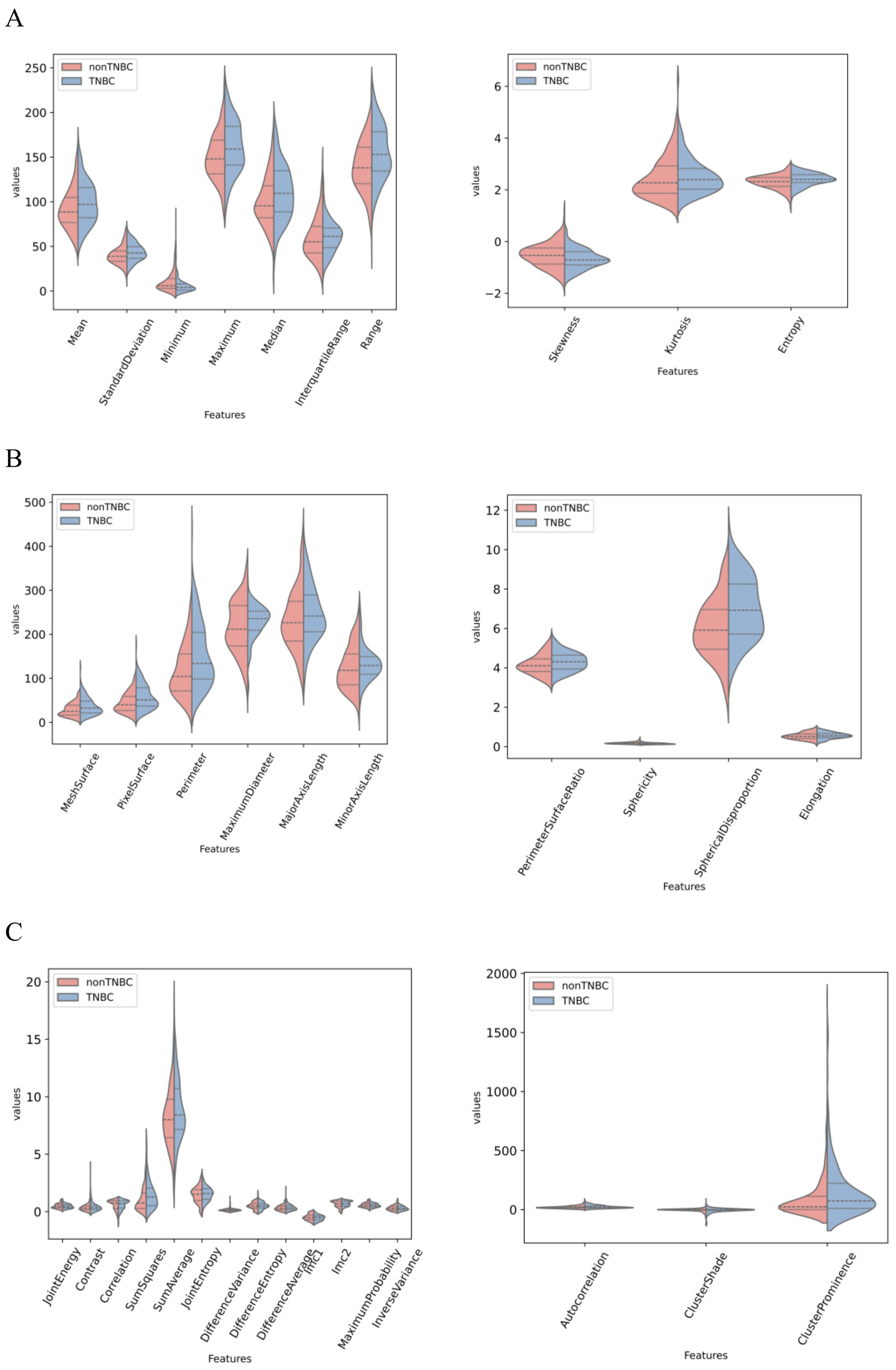

Fig. S13. The distribution of CAF-related radiomic features in radiomics cohort of NACT-Pilot. (A) The distribution of 10 first-order features in TNBC and non-TNBC groups. **(B)** The distribution of 10 shape2D features in TNBC and non-

TNBC groups. **(C)** The distribution of 16 GLCM features in TNBC and non-TNBC groups.

Fig. S14 shows the distribution of CAF-related radiomic features in TNBC and non-TNBC groups in clinical cohort. Fig. S14(A) is the first-order features and Fig. S14(B) is Shape2D features, Fig. S14(C) is the GLCM features.

As shown in Fig. S14, the TNBC group has a higher distribution in Kurtosis, Mean, Median, ClusterShade, Contrast, DifferenceAverage, DifferenceEntropy, DifferenceVariance, Imc1, InverseVariance and Sphericity, a lower distribution in Entropy, InterquartileRange, Range, Skewness, StandardDeviation, ClusterProminence, Imc2, MaximumProbability, MajorAxisLength, MaximumDiameter, MeshSurface, MinorAxisLength, Perimeter, PixelSurface than non-TNBC group, the same as the distribution of TNBC radiomic features in radiogenomics discovery cohort.

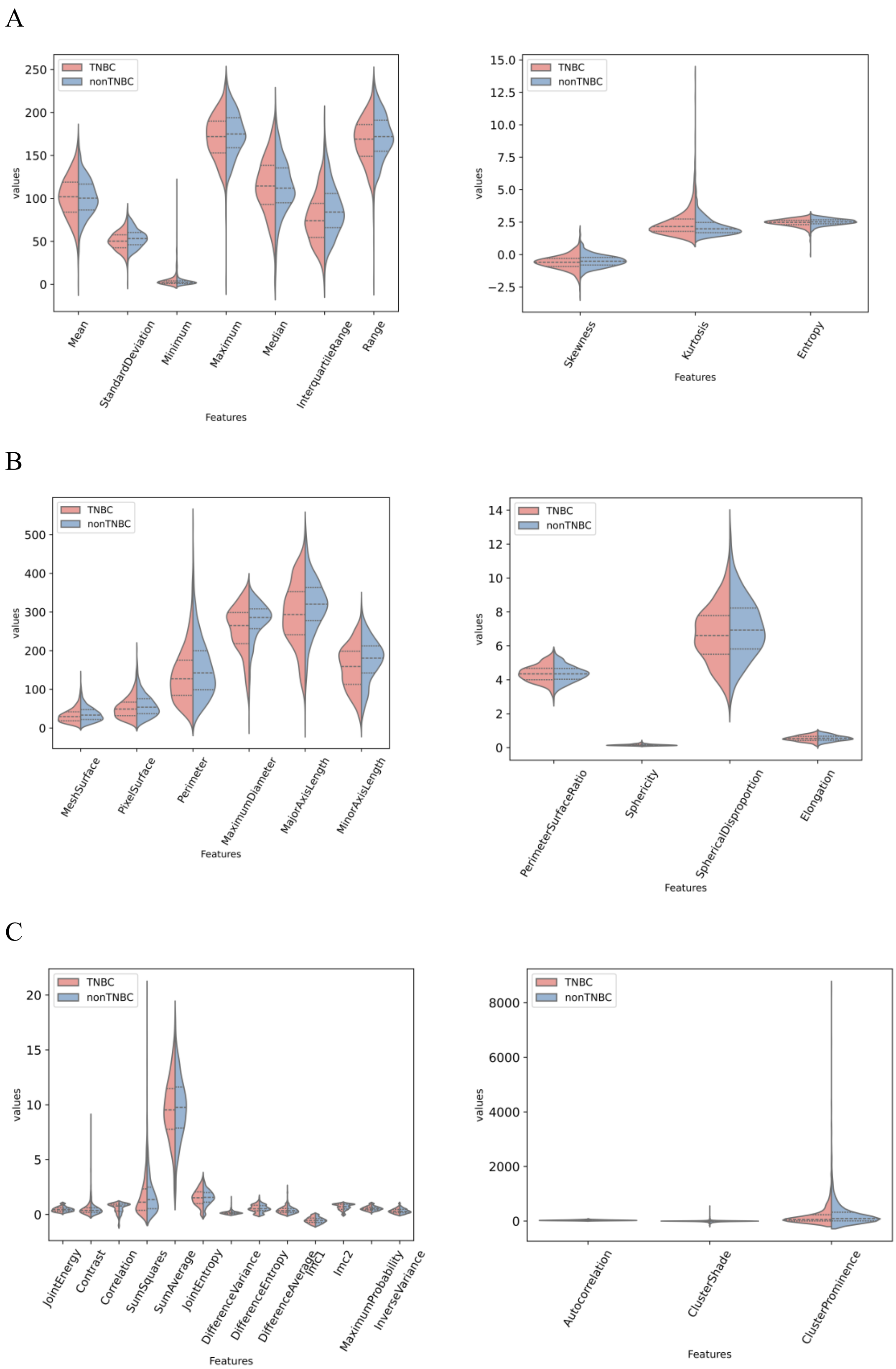

Fig. S14. The distribution of CAF-related radiomic features of TNBC and non-TNBC groups in clinical cohort. (A) The distribution of 10 first-order features in TNBC and non-TNBC groups. **(B)** The distribution of 10 shape2D features in

TNBC and non-TNBC groups. **(C)** The distribution of 16 GLCM features in TNBC and non-TNBC groups.

In all these datasets, the TNBC group has a high distribution in Kurtosis and Mean, a lower distribution in Skewness than non-TNBC group, indicating that these features can serve as an important feature metric to guide the classification of TNBC and non-TNBC groups.

Important gene features resulted in marker genes and made model explainable

Based on SMLP module, we can obtain the important gene features in image-CAF and image-TNBC models. As shown in Tables S9 and S10, the top 10 important genes identified by our model and their p-values and logFC (logarithmic fold change) in differentially expressed gene (DEG) analysis are demonstrated. As shown in Table S9 and Table S10, ERBB3 and PRLR are significantly downregulated in TNBC group, while TPM4, CCT6A, ENO1, S100A11, LSR, TUBB, SLC7A5 are significantly up regulate (adjusted p-value < 0.05).

The results show that some of the identified important genes exhibit significant differences between TNBC and non-TNBC groups, which strongly demonstrates that our model does have the ability to identify key genes and is expected to provide new clues to unravel some unknown biological functions. And the descriptions of these important genes in image-TNBC model and image-CAF model are as shown in Table 1 (in the article) and Table S11, respectively. In image-CAF and image-TNBC models, there is one overlapping gene, CRABP2, which is related to breast cancer. And some of genes in these two models (KRT8, KRT18, CCT6A, ACTB, ENO1, TUBB, SLC7A5, MUCL1, PRLR, IGF1R, RPN2, ERBB3) are related to TNBC subtype in current research as shown in description in Table 1 and Table S11. The rest of the genes are also associated with breast cancer.

Table S9. Top 10 important genes and the related information in image-TNBC model.

Gene	logFC	P.Value	adj.P.Value
KRT8	-43551.69877	0.02228059	0.094162361
CCT6A	20485.26044	0.002818039	0.019922948
KRT18	-29075.67904	0.000101134	0.001384691
CRABP2	9740.615754	0.03737795	0.135906476
ACTB	35907.0024	0.011791667	0.059416334
ENO1	28547.38093	7.74E-10	7.43E-08
S100A11	25451.27156	6.27E-10	6.40E-08
LSR	6106.146842	0.0024696	0.017884455
TUBB	15648.63477	4.56E-06	0.000105098
SLC7A5	14300.12295	6.79E-12	1.44E-09

Table S10. Top 10 important genes and the related information in image-CAF model.

Gene	logFC	P.Value	adj.P.Value
MUCL1	-6334.614211	0.604357726	0.806133551
CTSD	-2433.998947	0.650326605	0.836098628
PRLR	-14657.72933	6.48E-06	0.000140957
BMPR1B	-5773.085333	0.088463117	0.247783398
IGF1R	-4394.496386	0.335379747	0.580733681
ERBB3	-11117.89014	0.000714507	0.00673767
CPB1	-3051.608404	0.272569456	0.513163569
CRABP2	9740.615754	0.03737795	0.135906476
TPM4	6792.903509	0.001883467	0.014536327
RPN2	-1785.921842	0.830450226	0.942059534

Table S11. Top 10 important genes and their descriptions in image-CAF model.

Gene name	Description	Reference
MUCL1	Mucin-like 1 (MUCL1) is identified as a breast-specific gene. S J Conley et al. shows that MUCL1 expression is highest in HER2-amplified breast tumors and inhibiting HER2 activity in tumor cells resulted in a decreased MUCL1 expression.	11
CTSD	Cathepsin D (CTSD) is a lysosomal protease and a marker of poor prognosis in breast cancer.	12
PRLR	The PRLR encodes a receptor for the anterior pituitary hormone, prolactin, and belongs to the type I cytokine receptor family. Behnaz Motamedi et al. proposed that Prolactin receptor (PRLR) is a possible prognostic marker in TNBC; it was associated with recurrence, overall survival and the stage of TNBC patients.	13

BMPR1B	The BMPR1B encodes a member of the bone morphogenetic protein (BMP) receptor family of transmembrane serine/threonine kinases. Allison et al. proved that overexpression of CYP2J2 in MDA-MB-468-derived breast cancer cells activates BMPR1B expression that may contribute to increased migration.	14
IGF1R	IGF1R (Insulin-Like Growth Factor 1 Receptor) is a Protein Coding gene. Hortog et al. suggested that IGF-1R expression has been associated with a shorter survival of TNBC patients with respect to estrogen receptor- (ER)-positive breast tumor patients.	15
ERBB3	The ERBB3 encodes a member of the epidermal growth factor receptor (EGFR) family of receptor tyrosine kinases. Tao et al. proposed that antagonism of EGFR and ERBB3 (HER3) enhances the response to inhibitors of the PI3K-Akt	16

	pathway in triple-negative breast cancer.	
CPB1	CPB1 (Carboxypeptidase B1) is a protein-coding gene. Pavel Bouchal et al. employed iTRAQ-2DLC-MS/MS proteomics to 24 lymph node-positive and 24 lymph node-negative grade 1 luminal A primary breast tumors and found that Increased levels of CPB1 were associated specifically with lymph node positive grade 1 tumors.	17
CRABP2	Cellular retinoic acid binding protein 2 (CRABP2) belongs to fatty acid binding protein (FABP) family which binds with all-trans retinoic acid (RA). Feng et al. suggested that CRABP2 can suppress invasion and metastasis of ER^+ breast cancer and promote invasion and metastasis of ER^- breast cancer by regulating the stability of Lats1 in vitro and in vivo.	18
TPM4	TPM4 (Tropomyosin 4) is a protein-coding gene. Based on proteomic analyses and immunohistochemistry,	19

RPN2	Maria Kabbage et al. revealed an increased expression of tropomyosin-4 in infiltrating ductal breast carcinomas (IDCAs) tumors. Ribophorin II (RPN2), part of the N-oligosaccharyltransferase complex, is highly expressed in breast cancer stem cells and is associated with tumor metastasis. Makiko Ono et al. examined immunohistochemically the expression levels of RPN2 and p53 in primary breast cancer tissues surgically resected from 218 patients and confirmed that the incidence of RPN2 expression was higher in the triple-negative breast cancer.	[20]

Interpretable Surrogate model revealed the radiomic features associated with TNBC

Based on the XGBoost to obtain the weight of important features. The top 10 features that play an important role in all models are extracted, and 12 of all features are found in at least two models. Out of these 12 features 7 features have the same distribution in most of the models.

As shown in Fig. S15, the SumAverage and Sphericity of marker region show higher values in the TNBC group, and Skewness has a lower distribution in TNBC group than non-TNBC in all cohorts.

The MaximumDiameter, InterquartileRange, and Entropy have lower distribution in TNBC than non-TNBC group in almost all datasets (4 out of 5). These features showed a consistent distribution in all the datasets, suggesting a remarkably correlation between these features and TNBC, demonstrating that CAF-I-marker successfully revealed radiomic features associated with TNBC, which provided important clues and rationale for us to further explore the common pathogenic mechanisms behind these features.

A

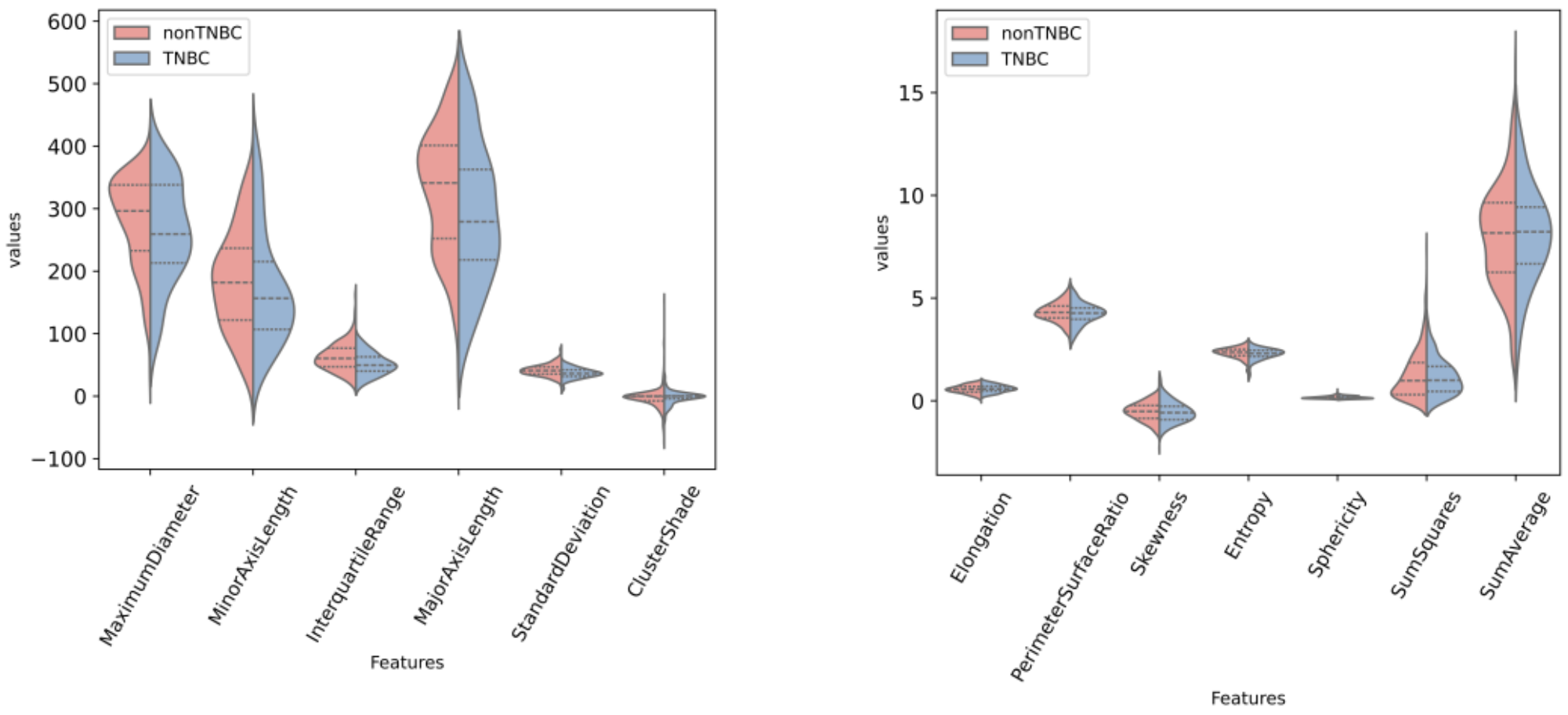
nonTNBC
TNBC
values
MaximumDiameter
MinorAxisLength
InterquartileRange
MajorAxisLength
StandardDeviation
ClusterShade
Features
nonTNBC
TNBC
values
Elongation
PerimeterSurfaceRatio
Skewness
Entropy
Sphericity
SumSquares
SumAverage
Features

B

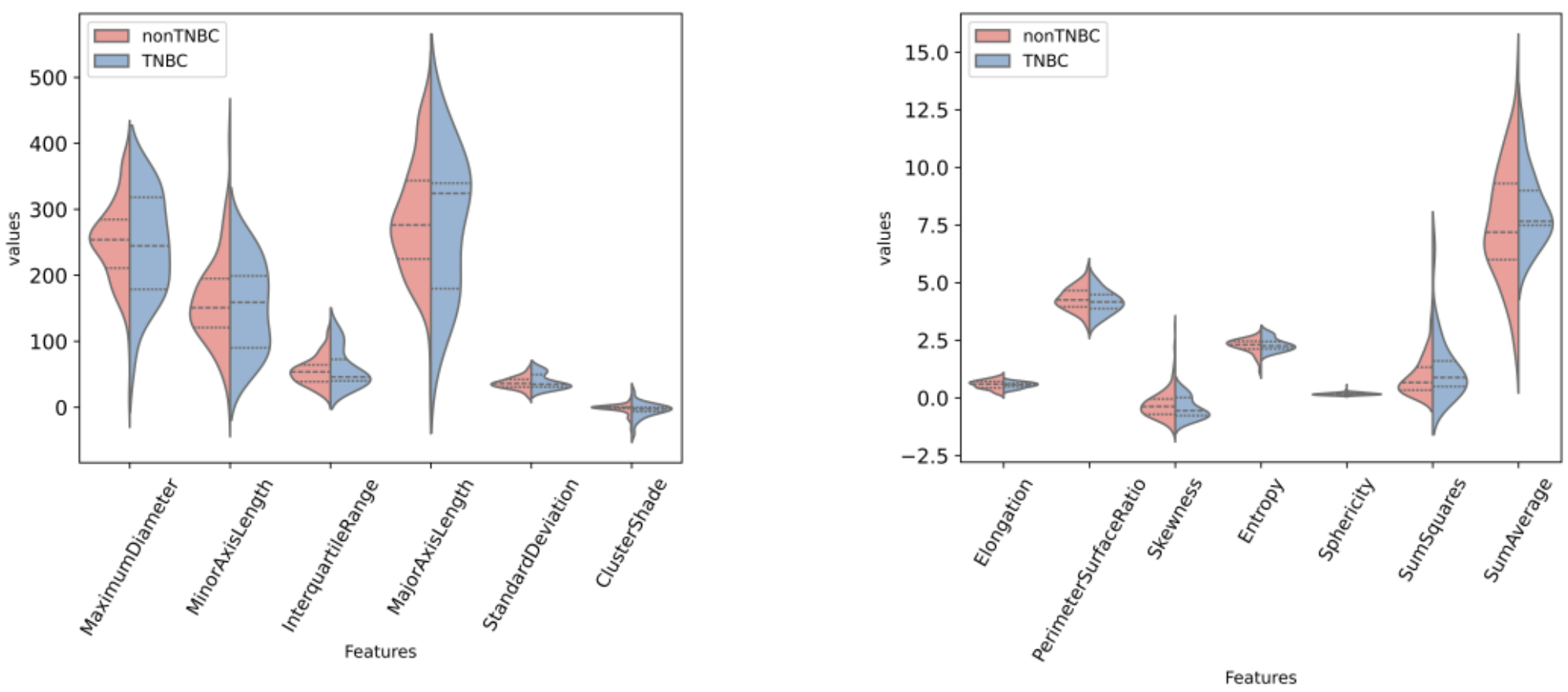
nonTNBC
TNBC
values
MaximumDiameter
MinorAxisLength
InterquartileRange
MajorAxisLength
StandardDeviation
ClusterShade
Features
nonTNBC
TNBC
values
Elongation
PerimeterSurfaceRatio
Skewness
Entropy
Sphericity
SumSquares
SumAverage
Features

C

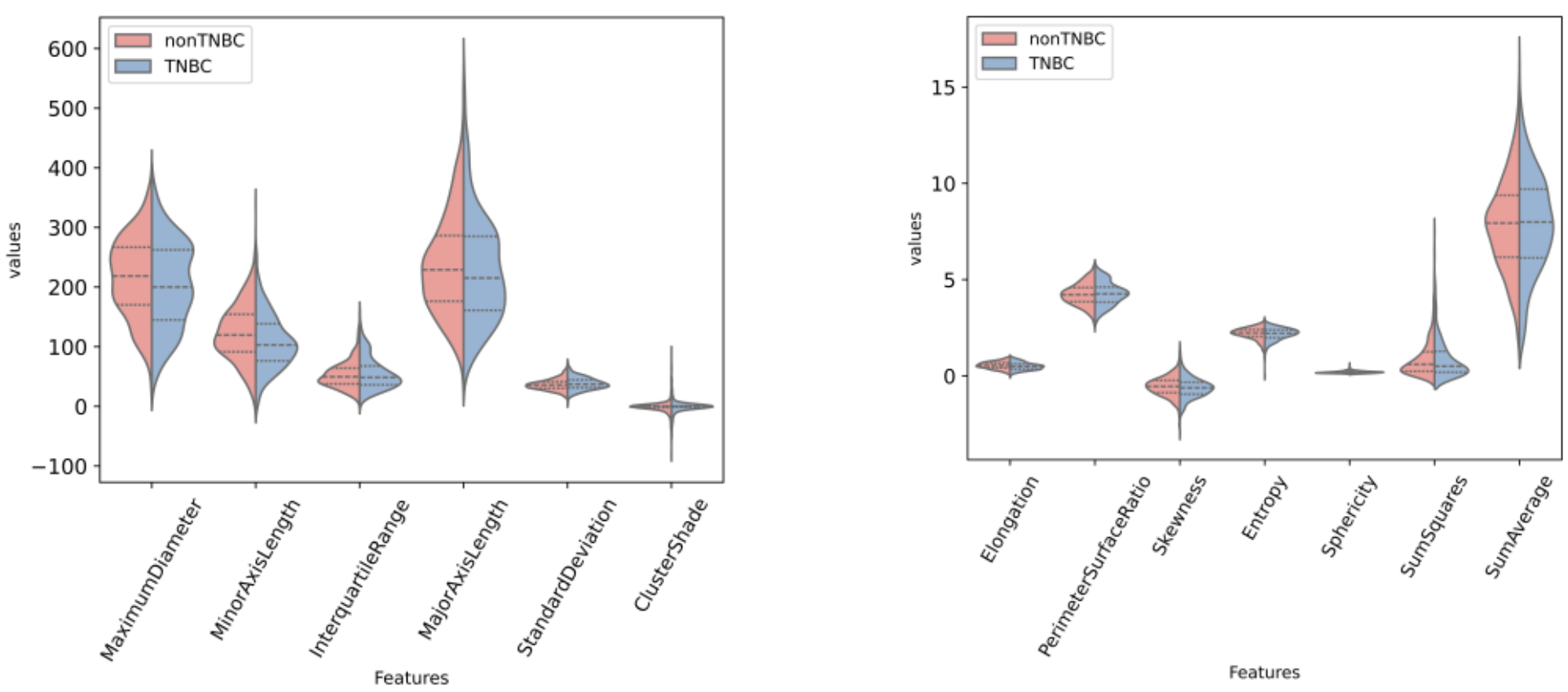
nonTNBC
TNBC
values
MaximumDiameter
MinorAxisLength
InterquartileRange
MajorAxisLength
StandardDeviation
ClusterShade
Features
nonTNBC
TNBC
values
Elongation
PerimeterSurfaceRatio
Skewness
Entropy
Sphericity
SumSquares
SumAverage
Features

D

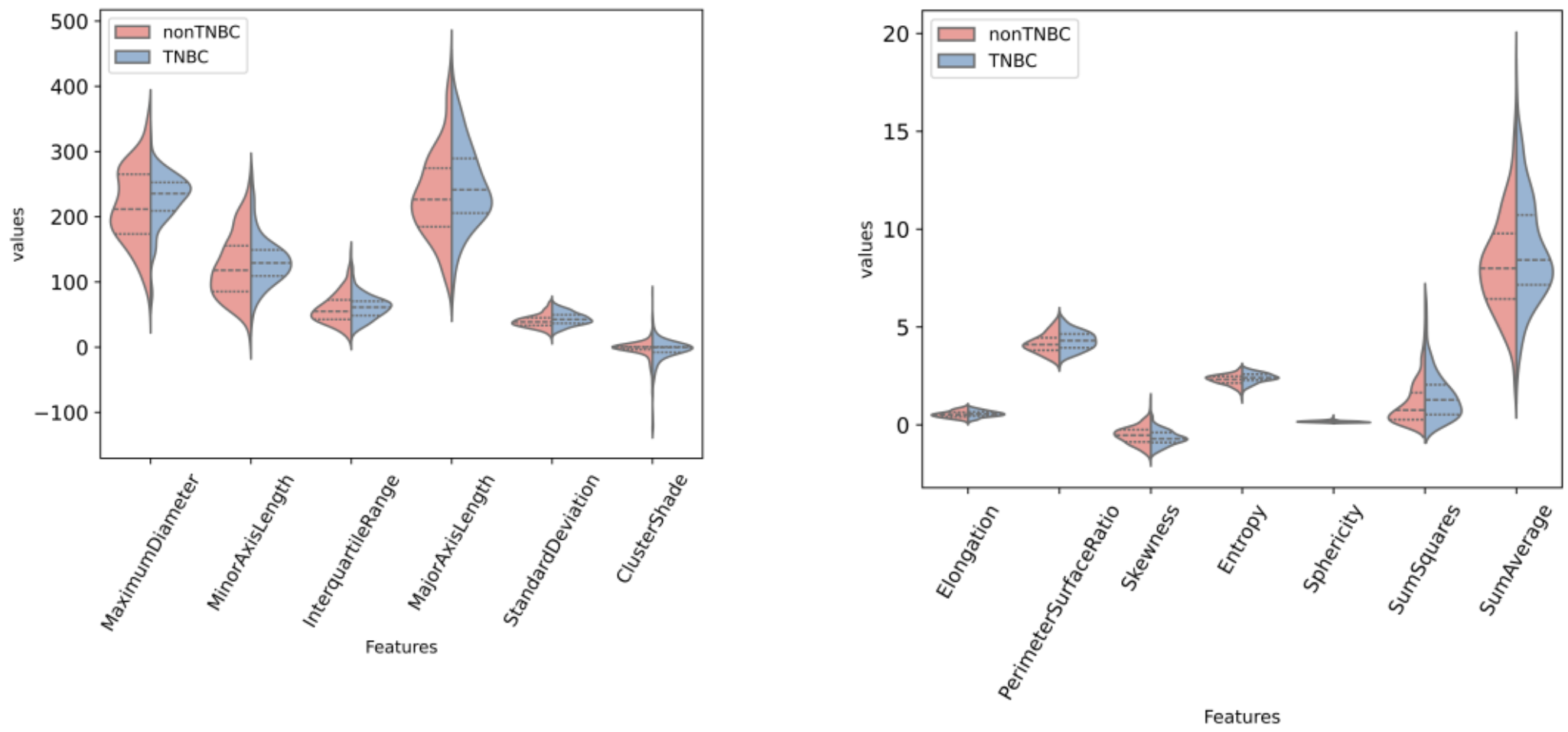

E

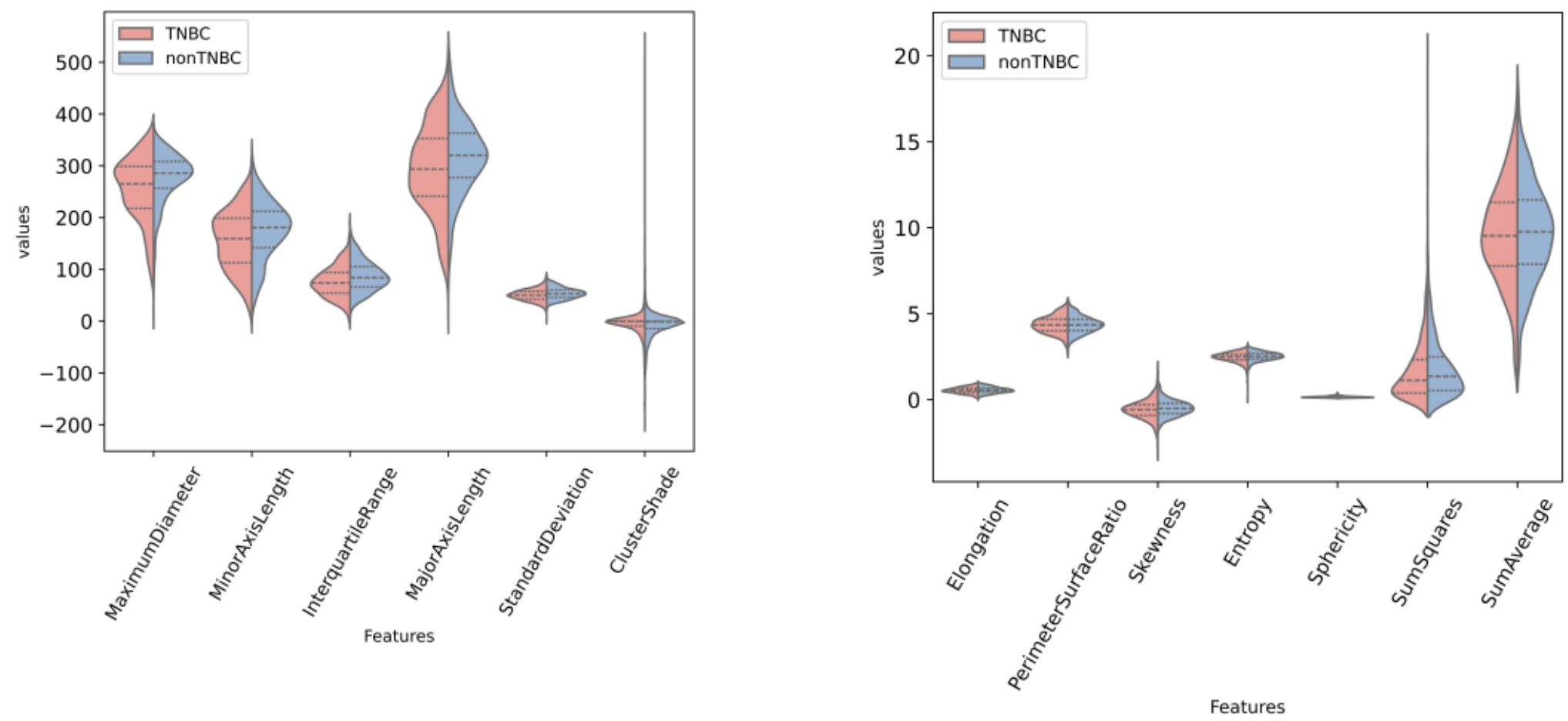

Fig. S15. The distribution of 7 important radiomic features in TNBC and non-TNBC groups in all datasets. **(A)** Radiogenomics discovery cohort. **(B)** Radiogenomics validation cohort. **(C)** ISPY of radiomics cohort. **(D)** NACT-Pilot of radiomics cohort. **(E)** Clinical cohort.

Samples enrolled in all datasets

The patient IDs included in radiogenomic cohort and radiomic cohort are shown in Table S12. The data of clinical cohort cannot be shared owing to patient-confidentiality obligations.

Table S12. The patient ID of radiogenomic cohort and radiomic cohort.

Dataset	Patient ID
Radiogenomics discovery cohort	TCGA-AO-A03M, TCGA-AO-A03V, TCGA-AO-A0J9, TCGA-AO-A0JI, TCGA-AO-A0JM, TCGA-AO-A12D, TCGA-AO-A12E, TCGA-AO-A12F, TCGA-AO-A12G, TCGA-AR-A1AN, TCGA-AR-A1AQ, TCGA-AR-A1AX, TCGA-AR-A24S, TCGA-AR-A24X, TCGA-BH-A0AW, TCGA-BH-A0AZ, TCGA-BH-A0B1, TCGA-BH-A0B3, TCGA-BH-A0B5, TCGA-BH-A0BG, TCGA-BH-A0BJ, TCGA-BH-A0BM, TCGA-BH-A0BQ, TCGA-BH-A0BT, TCGA-BH-A0C0, TCGA-BH-A0DE, TCGA-BH-A0DG, TCGA-BH-A0DH, TCGA-BH-A0DI, TCGA-BH-A0DK, TCGA-BH-A0DT, TCGA-BH-A0DV, TCGA-BH-A0DX, TCGA-BH-A0DZ, TCGA-BH-A0E0, TCGA-BH-A0E1, TCGA-BH-A0E2, TCGA-BH-A0E9, TCGA-BH-A0EI, TCGA-BH-A0GY, TCGA-BH-A0GZ, TCGA-BH-A0H3, TCGA-BH-A0H6, TCGA-BH-A0H7, TCGA-BH-A0H9, TCGA-BH-A0HA, TCGA-BH-A0HI, TCGA-BH-A0HX, TCGA-BH-A0HY, TCGA-BH-A0RX, TCGA-BH-A0W3, TCGA-BH-A0W5, TCGA-BH-A18F, TCGA-BH-A18H, TCGA-BH-A18I, TCGA-BH-A201, TCGA-BH-A202, TCGA-BH-A28Q, TCGA-E2-A109, TCGA-E2-A10F, TCGA-E2-A14P, TCGA-E2-A14S, TCGA-E2-A14T, TCGA-E2-A14V, TCGA-E2-A14Y, TCGA-E2-A150, TCGA-E2-A159, TCGA-E2-A15A, TCGA-E2-A15E, TCGA-E2-A15F, TCGA-E2-A15H, TCGA-E2-A15I, TCGA-E2-A15J, TCGA-E2-A15K, TCGA-E2-A15L, TCGA-E2-A15M, TCGA-E2-A1B1, TCGA-E2-A1B5, TCGA-E2-A1B6, TCGA-E2-A1IE, TCGA-E2-A1IG, TCGA-E2-A1II, TCGA-E2-A1IJ, TCGA-E2-A1IK, TCGA-E2-A1IN, TCGA-E2-A1L7, TCGA-E2-A1L9, TCGA-E2-A1LG
Radiogenomics validation cohort	TCGA-E2-A14Q, TCGA-EW-A1PA, TCGA-EW-A1P4, TCGA-EW-A1P3, TCGA-AO-A0J8, TCGA-EW-A2FS, TCGA-AR-A24R, TCGA-AO-A0JF, TCGA-AR-A254, TCGA-EW-A1IY, TCGA-EW-A1OY, TCGA-OL-A5DA, TCGA-AO-A0JB, TCGA-AR-A24U, TCGA-E2-A107, TCGA-OL-A5D8, TCGA-EW-A1P0, TCGA-AR-A24M,

	TCGA-AO-A129, TCGA-EW-A3E8, TCGA-AR-A24W, TCGA-EW-A1IZ, TCGA-EW-A1OZ, TCGA-EW-A2FR, TCGA-E2-A14X, TCGA-EW-A1J6, TCGA-E2-A105, TCGA-E2-A14N, TCGA-E2-A152, TCGA-BH-A0H5
ISPY of radiomic cohort	ISPY1_1002, ISPY1_1003, ISPY1_1004, ISPY1_1005, ISPY1_1007, ISPY1_1008, ISPY1_1009, ISPY1_1010, ISPY1_1011, ISPY1_1012, ISPY1_1013, ISPY1_1015, ISPY1_1016, ISPY1_1017, ISPY1_1021, ISPY1_1022, ISPY1_1025, ISPY1_1027, ISPY1_1029, ISPY1_1030, ISPY1_1031, ISPY1_1032, ISPY1_1034, ISPY1_1035, ISPY1_1036, ISPY1_1037, ISPY1_1038, ISPY1_1040, ISPY1_1041, ISPY1_1042, ISPY1_1043, ISPY1_1045, ISPY1_1046, ISPY1_1047, ISPY1_1051, ISPY1_1053, ISPY1_1054, ISPY1_1055, ISPY1_1056, ISPY1_1057, ISPY1_1058, ISPY1_1061, ISPY1_1062, ISPY1_1063, ISPY1_1064, ISPY1_1066, ISPY1_1069, ISPY1_1070, ISPY1_1071, ISPY1_1073, ISPY1_1075, ISPY1_1077, ISPY1_1078, ISPY1_1082, ISPY1_1083, ISPY1_1086, ISPY1_1087, ISPY1_1088, ISPY1_1090, ISPY1_1091, ISPY1_1092, ISPY1_1094, ISPY1_1095, ISPY1_1096, ISPY1_1097, ISPY1_1099, ISPY1_1100, ISPY1_1101, ISPY1_1102, ISPY1_1107, ISPY1_1110, ISPY1_1111, ISPY1_1112, ISPY1_1113, ISPY1_1114, ISPY1_1115, ISPY1_1116, ISPY1_1117, ISPY1_1121, ISPY1_1122, ISPY1_1123
NACT-Pilot of radiomic cohort	UCSF-BR-01, UCSF-BR-02, UCSF-BR-03, UCSF-BR-04, UCSF-BR-06, UCSF-BR-08, UCSF-BR-09, UCSF-BR-11, UCSF-BR-14, UCSF-BR-15, UCSF-BR-18, UCSF-BR-22, UCSF-BR-23, UCSF-BR-26, UCSF-BR-27, UCSF-BR-29, UCSF-BR-32, UCSF-BR-34, UCSF-BR-36, UCSF-BR-37, UCSF-BR-39, UCSF-BR-41, UCSF-BR-43, UCSF-BR-44, UCSF-BR-45, UCSF-BR-47, UCSF-BR-48, UCSF-BR-49, UCSF-BR-50, UCSF-BR-52, UCSF-BR-54, UCSF-BR-58, UCSF-BR-59, UCSF-BR-60, UCSF-BR-62, UCSF-BR-63, UCSF-BR-66, UCSF-BR-67, UCSF-BR-68

References

1. Wang, H. et al. miRNA-195-5p/PSAT1 feedback loop in human triple-negative breast cancer cells. *Genes* **45**, 39-47 (2023).
2. Sanawar, R., Mohan Dan, V., Santhoshkumar, T.R., Kumar, R. & Pillai, M.R. Estrogen receptor-α regulation of microRNA-590 targets FAM171A1—a modifier of breast cancer invasiveness. *Oncogenesis* **8**, 5 (2019).
3. Zhang, Y. et al. LncRNA-BC069792 suppresses tumor progression by targeting KCNQ4 in breast cancer. *Mol Cancer* **22**, 41 (2023).
4. Liu, X. et al. A pro-metastatic tRNA fragment drives Nucleolin oligomerization and stabilization of its bound metabolic mRNAs. *Molecular cell* **82**, 2604-2617.e2608 (2022).
5. Zhao, Z.-M. et al. CCNE1 amplification is associated with poor prognosis in patients with triple negative breast cancer. *BMC Cancer* **19**, 96 (2019).
6. Bao, C. et al. Exploring specific prognostic biomarkers in triple-negative breast cancer. *Cell death & disease* **10**, 807 (2019).
7. Luo, Y., Hou, Z., Feng, G., Xiong, D. & Chen, J. CDCA2 promotes breast cancer progression by downregulating KISS1 expression. *European Journal of Gynaecological Oncology* **45**, 127-134 (2024).
8. Addou-Klouche, L. et al. Loss, mutation and deregulation of L3MBTL4 in breast cancers. *Molecular Cancer* **9**, 213 (2010).
9. Sgubin, M. et al. HMGA1 positively regulates the microtubule-destabilizing protein stathmin promoting motility in TNBC cells and decreasing tumour sensitivity to paclitaxel. *Cell death & disease* **13**, 429 (2022).
10. Arnold, J. et al. Tubulin Tyrosine Ligase Like 4 (TTLL4) overexpression in breast cancer cells is associated with brain metastasis and alters exosome biogenesis. *Journal of Experimental & Clinical Cancer Research* **39**, 205 (2020).
11. Conley, S.J. et al. HER2 drives Mucin-like 1 to control proliferation in breast cancer cells. *Oncogene* (2016).
12. Ketterer, S. et al. Cathepsin D deficiency in mammary epithelium transiently stalls breast cancer by interference with mTORC1 signaling. *Nat. Commun.* **11**, 5133 (2020).
13. Motamedi, B. et al. Prolactin receptor expression as a novel prognostic biomarker for triple negative breast cancer patients. *Annals of Diagnostic Pathology* **46**, 151507 (2020).
14. Allison, S.E. et al. Activation of the pro-migratory bone morphogenetic protein receptor 1B gene in human MDA-MB-468 triple-negative breast cancer cells that over-express CYP2J2. *The International Journal of Biochemistry & Cell Biology* **80**, 173-178 (2016).
15. Hartog, H. et al. Divergent effects of insulin-like growth factor-1 receptor expression on prognosis of estrogen receptor positive versus triple negative invasive ductal breast carcinoma. *Breast cancer research and treatment* **129**, 725-736 (2011).
16. Tao, J.J. et al. Antagonism of EGFR and HER3 enhances the response to inhibitors of the PI3K-Akt pathway in triple-negative breast cancer. *Science signaling* **7**, ra29 (2014).
17. Bouchal, P. et al. Combined Proteomics and Transcriptomics Identifies Carboxypeptidase B1 and Nuclear Factor κB (NF-κB) Associated Proteins as Putative Biomarkers of Metastasis in Low Grade Breast Cancer. *Molecular & Cellular Proteomics* **14**, 1814-1830 (2015).
18. Feng, X. et al. CRABP2 regulates invasion and metastasis of breast cancer through hippo pathway dependent on ER status. *Journal of Experimental & Clinical Cancer Research* **38**, 361 (2019).

19. Kabbage, M. et al. Tropomyosin-4 correlates with higher SBR grades and tubular differentiation in infiltrating ductal breast carcinomas: an immunohistochemical and proteomics-based study. *Tumor Biology* **34**, 3593-3602 (2013).
20. Ono, M. et al. The expression and clinical significance of ribophorin II (RPN2) in human breast cancer. *Pathology international* **65**, 301-308 (2015).
